# The gene repertoire of the main cysteine protease of *Trypanosoma cruzi*, cruzipain, reveals four sub-types with distinct active sites

**DOI:** 10.1038/s41598-021-97490-2

**Published:** 2021-09-14

**Authors:** Viviane Corrêa Santos, Antonio Edson Rocha Oliveira, Augusto César Broilo Campos, João Luís Reis-Cunha, Daniella Castanheira Bartholomeu, Santuza Maria Ribeiro Teixeira, Ana Paula C. A. Lima, Rafaela Salgado Ferreira

**Affiliations:** 1 Departamento de Bioquímica e Imunologia, Universidade Federal de Minas Gerais, Belo Horizonte, MG Brazil; 2 Departamento de Análises Clínicas e Toxicológicas, Faculdade de Ciências Farmacêuticas, Universidade de São Paulo, São Paulo, Brazil; 3 Departamento de Parasitologia, Universidade Federal de Minas Gerais, Belo Horizonte, MG Brazil; 4 Departamento de Medicina Veterinária Preventiva, Escola de Veterinária, Universidade Federal de Minas Gerais, Belo Horizonte, MG Brazil; 5 Instituto de Biofísica Carlos Chagas Filho, Universidade Federal do Rio de Janeiro, Rio de Janeiro, RJ Brazil

**Keywords:** Computational biology and bioinformatics, Proteases

## Abstract

Cruzipains are the main papain-like cysteine proteases of *Trypanosoma cruzi*, the protozoan parasite that causes Chagas disease. Encoded by a multigenic family, previous studies have estimated the presence of dozens of copies spread over multiple chromosomes in different parasite strains. Here, we describe the complete gene repertoire of cruzipain in three parasite strains, their genomic organization, and expression pattern throughout the parasite life cycle. Furthermore, we have analyzed primary sequence variations among distinct family members as well as structural differences between the main groups of cruzipains. Based on phylogenetic inferences and residue positions crucial for enzyme function and specificity, we propose the classification of cruzipains into two families (I and II), whose genes are distributed in two or three separate clusters in the parasite genome, according with the strain. Family I comprises nearly identical copies to the previously characterized cruzipain 1/cruzain, whereas Family II encompasses three structurally distinct sub-types, named cruzipain 2, cruzipain 3, and cruzipain 4. RNA-seq data derived from the CL Brener strain indicates that Family I genes are mainly expressed by epimastigotes, whereas trypomastigotes mainly express Family II genes. Significant differences in the active sites among the enzyme sub-types were also identified, which may play a role in their substrate selectivity and impact their inhibition by small molecules.

## Introduction

*Trypanosoma cruzi*, the causative agent of Chagas disease, is a digenetic, intracellular parasite transmitted by triatomine insects to mammalian hosts such as humans, marsupials, and rodents. This parasite has three life cycle stages: trypomastigotes and amastigotes are found in the mammalian hosts, while epimastigotes proliferate in the insect vector^1^. Cruzipain is a papain-like cysteine protease of *T. cruzi*, expressed in all life cycle stages of the parasite^2^ and is involved in metacyclogenesis, host cell invasion, and modulation of the host cell response^3–9^. Based on its biological importance, as well as studies with inhibitors, cruzipain has been validated as a target for drug design in Chagas disease. The term ‘cruzipain’ refers to the native enzyme^10,11^, whereas its recombinant form lacking the C-terminal extension was named ‘cruzain’^2^. Several classes of cruzain inhibitors have been described, these include vinyl sulfones, tetrafluorophenoxymethyl ketones, benzimidazoles, oxyguanidine derivatives, and peptidyl nitroalkenes^12^. These compounds have shown efficacy against *T. cruzi *in vitro as well as in animal models of infection^13,14^. Among them, the vinyl sulfone K11777 has progressed to late preclinical trial stages^15^.

Although much of the literature refers only to cruzipain or cruzain, giving the impression that there is a single enzyme, different cruzipain sub-types have been described. The ‘classic’ and most studied cruzipain, first named in 1990^10,11^, is now referred to as cruzipain 1 (czp1), but a second sub-type, described in 1994, was named cruzipain 2 (czp2)^16^. Czp1 and czp2 differ in their behavior with different substrates and inhibitors. Czp2 is less sensitive to E-64 and has different substrate specificity, particularly in its S2 subsite^17,18^. Differences in sensitivity to cystatin C have also been described, indicating lower efficiency of this mammalian inhibitor against czp2^17^, as well as reduced sensitivity to modulation by heparan sulfate^19^. Western blot analyses with protein extracts from the *T. cruzi* Dm28c strain have shown that epimastigotes express mainly czp1, while the trypomastigote forms preferably express czp2^17^. Furthermore, polymorphisms within the czp1 and czp2 sequences of a given *T. cruzi* strain and among different strains have been described, being less frequent or absent in czp1 copies depending on the strain evaluated^16,20^. Although initial genome studies have shown that polymorphic genes are organized in tandem in the genomes of different parasite strains, the total number of copies, organization, and genome location of the cruzipain genes remain unclear^2,21^. Depending on the strain, two to four cruzipain clusters were proposed to exist in one or two chromosomes, with each cluster containing ten to one hundred and thirty gene copies^21^. Although complete genome sequences from different parasite strains have been described since 2005, because of its repetitive nature, which resulted in collapsed repetitive regions during genome assembly, a precise evaluation of the exact copy numbers and organization of several multigene families have not yet been determined. Synteny analysis revealed a region on chromosome 6 of the CL Brener strain containing a cluster of cruzipain or orthologous genes, with the same organization found in several *T. cruzi* strains as well as in the related species, *T. dionisii* and *T. brucei*^20^.

Recently, additional sequencing data generated with the PacBio and Nanopore platforms, which provide long sequencing reads, allowed the precise assembly of regions containing tandem repeats in different *T. cruzi* strains^22–24^. Here, we investigated the copy number and genomic organization of cruzipain genes in CL Brener, Dm28c, and YC6 strains based on this new genomic information. We identified all cruzipain sequences present in the genomes of these three strains and showed that they can be grouped into four sub-types according to signatures found in their active site architecture. Additionally, available RNA-seq data allowed us to determine the transcriptional profiles for each group of cruzipain genes throughout the different stages of the parasite life cycle. We also evaluated the impact of residue substitutions observed in different cruzipain sub-types, employing comparative modeling to predict their consequences on active site structure and drug design.

## Results

### Sequence divergence and genomic organization of cruzipain genes in different *T. cruzi* strains

**Figure 1:**
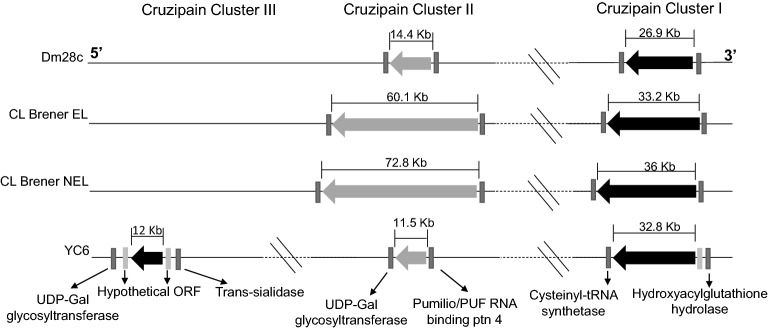
Cruzipain genes from Dm28c (DTU-TcI), CL Brener (DTU-TcVI), and YC6 (DTU-TcII) *T. cruzi* strains are organized in two or three clusters. Organization of cruzipain gene copies in the *T. cruzi* genome. The clusters are displayed in the 5′ to 3′ direction according to the genome localization. Each cluster is represented as a horizontal arrow indicating the translation direction. Dm28c: Dm28c strain; CL Brener EL: CL Brener strain Esmeraldo-like haplotype; CL Brener NEL: CL Brener strain non-Esmeraldo-like haplotype, YC6: Y strain. The flanking genes are shown as rectangles with the functional ORFs in dark gray and the hypothetical ORFs in light gray. The clusters are located in chromosome 6 of the CL Brener and chromosome 35 of the YC6 strain. The chromosome has not been assigned in Dm28c.

Genomic analyses of *T. cruzi* CL Brener (DTU-TcVI), Dm28c (DTU-TcI), and YC6 (DTU-TcII) strains based on PacBio and Nanopore sequencing, together with the current assembly of the CL Brener strain available at TriTrypDB.org, which was based on Sanger sequencing, revealed two regions in the same chromosome containing cruzipain gene clusters in the genomes of all three parasite strains. For the CL Brener strain, which has a hybrid genome, we show the location of the two clusters in both haplotypes that were separately assembled (Esmeraldo-like, EL and non-Esmeraldo-like, NEL) (Fig. 1). Although high synteny levels with flanking genes were observed, significant copy number variation within each cluster between each genome was also detected, as shown in Fig. 1. It is noteworthy that copy number variation was also observed when the two CL Brener haplotypes were compared. According to the assembly available in TriTrypDB, the two clusters are located on chromosome 6 of the CL Brener strain and on chromosome 35 of the YC6 strain (Fig. 1). The number of gene copies in each cluster is depicted in Table 1.

**Table 1:** Number of cruzipain copies per cluster in Dm28c, CL Brener, and YC6 genomes.

Strain/haplotype	Cluster I		Cluster II		Cluster III	
	Complete copies	Truncated copies	Complete copies	Truncated copies	Complete copies	Truncated copies
Dm28c	14	1	7	3	–	–
CL Brener EL	5	5	2	3	–	–
CL Brener NEL	10	8	3	8	–	–
YC6	14	4	6	2	6	1

In the genomes of all three strains, the first region or cluster (Cluster I) is flanked by the genes encoding “cysteinyl-tRNA synthetase” and “hydroxyacylglutathione hydrolase” . In the Dm28c genome, Cluster I contains fourteen nearly identical complete cruzipain copies in tandem (> 99% protein sequence identity) and one truncated copy of the gene (Table 1). In CL Brener, Cluster I contains five tandem complete gene copies in the EL haplotype and ten copies in the NEL haplotype. In the EL haplotype, there are five truncated genes, while there are eight in the NEL haplotype. As observed for Dm28c, in both CL Brener haplotypes, all cruzipain sequences in Cluster I have > 99% predicted protein sequence identity (Supplementary Table S1). Cluster I in the YC6 genome contains fourteen complete copies and four truncated cruzipain copies. The complete copies are almost identical, with > 99% protein sequence identity (Supplementary Table S1).

In all three strains, the second cluster (Cluster II) is flanked by the “UDP-Gal glycosyltransferase” gene and by the “pumilio/PUF RNA binding Protein 4” gene . In Dm28c, Cluster II contains nine copies in tandem, of which seven encode complete cruzipain sequences sharing > 91% protein identity, and < 89% protein identity with the sequences of Cluster I, while the remaining two copies are of truncated genes (Fig. 1, Supplementary Table S1). In Dm28c, a tenth truncated single gene copy is located further down from Cluster II. In the CL Brener EL haplotype, Cluster II has five copies of cruzipain, whereas in the CL Brener NEL haplotype, there are eleven copies. Remarkably, only two and three copies in the EL and NEL haplotypes, respectively, encode full-length protein, and therefore, the CL Brener potentially encodes five functional cruzipains in Cluster II (Table 1, Supplementary Fig. 1). In YC6, Cluster II has six complete cruzipain copies with > 99% protein sequence identity, as well as two truncated copies.

Notably, the YC6 strain has a third cluster containing six complete cruzipain copies, flanked by two “Hypothetical ORFs”, a “UDP-Gal glycosyltransferase” gene and ‘trans-sialidase” gene, which is located to the left of the other two aforementioned clusters. Sequences in Cluster III share > 96% protein sequence identity. This cluster also contains one truncated copy that is more similar to cruzipain sequences found in Cluster II (Fig. 1, Table 1 and Supplementary Table S1).

The analysis of the truncated cruzipain gene sequences from the three strains revealed a great variety of frameshifts that led to truncations in different domains of the protein (Supplementary Fig. 1). Full-length cruzipain is constituted of a signal peptide (pre-region), a prodomain that is required for protein folding and is released from the zymogen upon enzyme maturation/activation, the central/catalytic domain that is responsible for enzymatic activity and a C-terminal extension^2^ (Supplementary Fig. 1 and Fig. 3A). In each strain, the truncation in one gene copy located in Cluster I localizes nearly at the end of the central (catalytic) domain (Val213), which could potentially result in the expression of a functional enzyme lacking the C-terminal extension. All other truncated gene copies, in the three parasite strains, lack significant portions of the gene sequence and the resulting products should not have protease activity. However, the great majority of truncated cruzipain genes, at least so in Dm28c and YC6 strains, retain intact the signal peptide and the prodomain, which could result in the production of proteins without enzymatic activity, but retaining a functional prodomain, that acts as an inhibitor of cruzipain, as described^25^.

The intergenic regions between cruzipain copies are highly similar within a cluster, sharing between 95 and 100% nucleotide identity within the tandem repeats of Cluster I when the three strains are compared (Supplementary Table 2, Supplementary Fig. 2). Likewise, intergenic regions from YC6 strain Cluster III are very similar to Cluster I sequences. The intergenic regions within the tandem copies of Cluster II of the three strains are also highly similar, sharing > 93% identity. Noteworthy, the intergenic regions of Cluster II are 62 nucleotides shorter than those of Cluster I, due to a deletion in the middle of the intergenic sequence. Interestingly, the deletion in Cluster II is observed at the same location in all three parasite strains (Supplementary Fig. S2).

### Cruzipain sequences can be grouped into two families and four sub-types

To evaluate the variability within cruzipain sequences, we performed an alignment of the predicted protein sequences for all full-length copies (sequences with the prodomain, central domain, and C-terminal extension) (Supplementary Table S1), as well as alignments of each domain separately (Supplementary Tables S3-S5). This analysis revealed that, while the regions corresponding to the preprodomain (corresponding to the signal peptide and prodomain, residues -122 to -1) and the C-terminal extension (residues 216 to 345) are virtually identical among them (> 93% and > 90% sequence identity, respectively), the central domain (residues 1 to 215) displays a higher degree of divergence (74–100% identity) (Supplementary Fig. S3 and Supplementary Tables S3-S5). As described previously, the C-terminal extension is not required for enzymatic activity, while the central domain possess all the residues required for proteolytic activity^2^.

**Figure 2:**
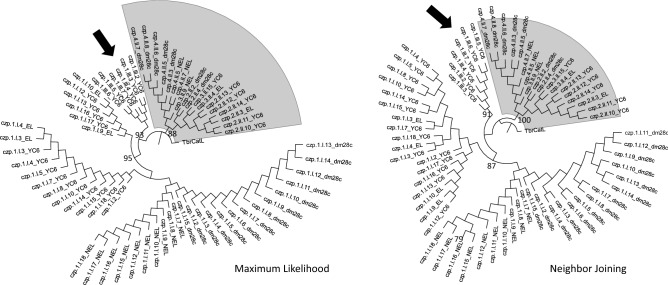
Phylogenetic analysis supports the division of cruzipain sequences into two families. The MEGA-X program was used to construct Maximum Likelihood and Neighbor Joining trees based on aligned amino acid sequences of the central domains of 67 genes encoding cruzipains in Dm28c, CL Brener, and YC6 *T. cruzi* strains. The numbers at the nodes are the bootstrap values, which were calculated based on 1000 replicates. Cruzipain sequences from Family II are highlighted in gray, and the arrows indicate the cruzipain sequences from Cluster III of the YC6 strain. The sequence of *Tbr*CatL (a.k.a. rhodesain, GenBank: CAC67416.1), the papain-like cysteine protease from *Trypanosoma brucei*, was used as root.

Phylogenetic analyses based solely on the central domain of all protein sequences indicated that cruzipain genes could be grouped into two major families that correlate with their distribution in each cluster (Fig. 2). Sequences from Cluster I are highly similar (> 97% identity) to cruzipain 1/cruzain^2^, with the CL Brener EL sequences grouping with YC6 sequences, while the CL Brener NEL sequences grouped with those of Dm28c. Sequences from Cluster II are more similar to the cruzipain 2 isoform previously described^16^, with the same grouping pattern being observed. Based on these analyses, we propose the classification of cruzipain sequences into two families: Family I, comprising the sequences from gene Cluster I, and Family II, comprising the sequences from gene Cluster II. Interestingly, in the YC6 strain, the third cluster contains copies of Family I and one truncated copy of Family II (indicated by the arrows in Fig. 2). These sequences from Cluster III group with the other Cluster I sequences.

The czp1 central domain sequences present in the different alleles of CL Brener, EL and NEL, share > 98% protein sequence identity. When czp1 sequences were compared between strains, a high level of conservation was maintained, i.e., 97% identity between czp1 from Dm28 and CL Brener, and 93% identity between Dm28 and YC6 strain (Supplementary Table S5).

**Figure 3:**
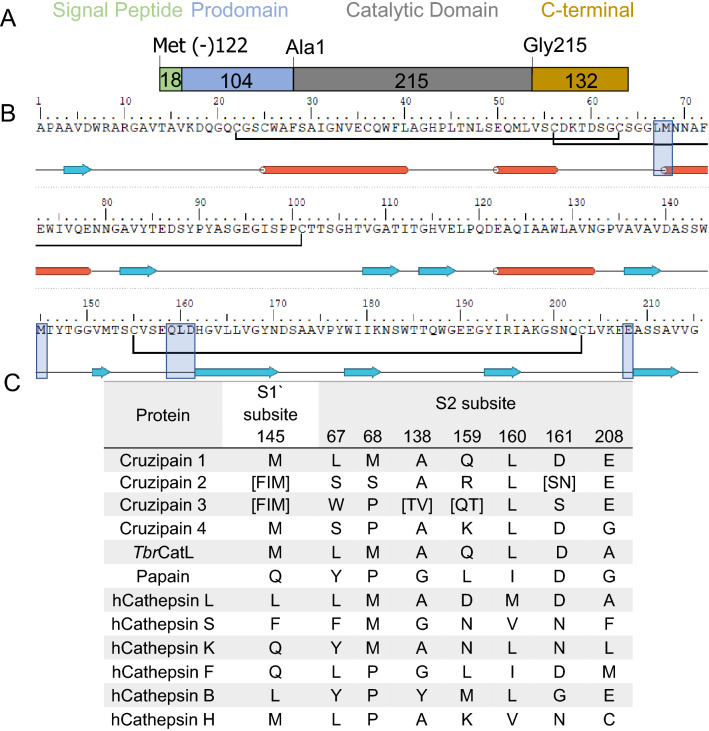
Cruzipain proteins can be divided into four sub-types based on structural motifs. (**A**) Schematic representation of the different domains within cruzipain. (**B**) Cruzipain 1/cruzain catalytic domain sequence with secondary structure assignment (PDB ID: 3KKU). Blue arrows indicate sheets and red cylinders indicate helices. Highlighted in blue boxes are the motifs used to define the different cruzipain sub-types. (**C**) Motifs employed to classify cruzipain sequences into sub-types and residues found in each motif for cruzipains, *Tbr*CatL (GenBank: CAC67416.1), and non-parasitic papain-like cysteine proteases – papain (UniProtKB: P00784), from *Carica papaya*, and human cathepsins retrieved from GenBank under the accession numbers: hCatL NP_001244901.1, hCatS AAC37592.1, hCatK NP_000387.1, hCatF AAF13146.1, hCatB NP_680093.1, hCatH AAL23961.1). Figure generated with Maestro 11.4, Schrödinger, LLC, New York, NY.

In contrast to the high sequence identity observed within Family I sequences, greater divergence was found among the cruzipain sequences of Family II. The sequence that was previously described as the czp2 isoform was identified in Cluster II of Dm28c^16,17^, as well as sequences that were further divergent from czp1 and czp2. Considering this high level of diversity, we asked if cruzipain sequences of Family II could be further divided into sub-types according to the substitutions of residues in the catalytic domain. Alignment of cruzipain DNA sequences of both families from Dm28c identified twenty-three SNPs (data not shown) and twelve positions with substitutions of two or more sequential amino acid residues in the catalytic domain (Supplementary Fig. S3). A larger number of amino acid substitutions was identified in the catalytic domain of cruzipains belonging to Family II. Furthermore, we could easily group cruzipain copies of the different strains according to the residues displayed at sequence divergence spots. Figure 3 shows a comparison of these sequences with a particular emphasis on motifs that are important for enzyme activity as previously defined for papain-like cysteine proteases and based on structural data of cruzain co-crystallized with ligands^26–28^.

We analyzed further the amino acid substitutions in the central/catalytic domain (Ala1 to Gly215) among cruzipain sequences (Fig. 3A,B). Based on their structural importance, we initially considered loops containing residues 67–70 and 158–162, located at the interfaces between protease subsites S2/S3 and S2/S1’ (Fig. 3C). We refined the positions within these motifs that could be considered in the definition of sub-type signatures, based on residues that frequently interact with cruzain inhibitors in crystallographic complexes (further details later described below and in Table 2). Considering frequent protein–ligand interactions, the following positions were determined to be signatures for cruzipain sub-types: 67, 68, 138, and 208, located in the S2 pocket; 145, in the S1’ region; and 159 and 161 in the interface between S2/S1’ sites. Importantly, residue 208 is crucial in conferring dual cathepsin-L and cathepsin B-like specificity to czp1^29,30^, while non-conservative substitutions in residues 68–70 were previously hypothesized as determinants for the different S2-specificity of cruzipain 2^17,18^. Based on the comparison of these positions, we proposed the division of cruzipain Family II into three sub-types: cruzipain 2, cruzipain 3, and cruzipain 4 (Fig. 3C). These signatures are found in multiple *T. cruzi* strains, namely CL Brener, Dm28c, YC6, and TCC (Supplementary Figures S3 and S4). It is worth mentioning that the modifications are consistently observed in the same regions, in all the sequences, with each sub-type having its conserved pattern. Since there are few random modifications, we usually observed the same modification in the same sub-type of all analyzed strains.

**Table 2:** Amino acids substitutions in the active site of cruzipains and frequency of interactions of each position with cruzain ligands.

Sub-type/Archetype^a^	Residue												
	S3		S2						S1	S1′			S2/S1′
*Cruzipain 1*	S61	N70	L67	M68	N69	A138	G163	E208	S64	S142	M145	D161	Q159
*Cruzipain 2*	F	K	S	S	L	A	A	E	G	T	M	N	R
*Cruzipain 3* (czp.3.II.4_EL)	F	K	W	P	L	T	A	E	G	T	I	S	T
*Cruzipain 3* (czp.3.II.9_NEL)	S	V	W	P	L	A	A	E	G	S	I	S	Q
*Cruzipain 4*	S	V	S	P	L	A	A	G	G	S	M	D	K
Frequency of interaction with crystallographic ligands (%)^b^	0.0	0.0	79.2	70.8	0.0	83.3	0.0	41.7	3.0	0.0	29.2	50.0	4.2

^a^We modeled one or two isoforms from each cruzipain sub-type, corresponding to sequences from czp.2.II.6_EL, czp.3.II.4_EL, czp.3.II.9_NEL and czp.4.II.7_NEL.

^b^Intermolecular interactions were analyzed with nAPOLI using ligands co-crystallized with cruzain.

**Figure 4:**
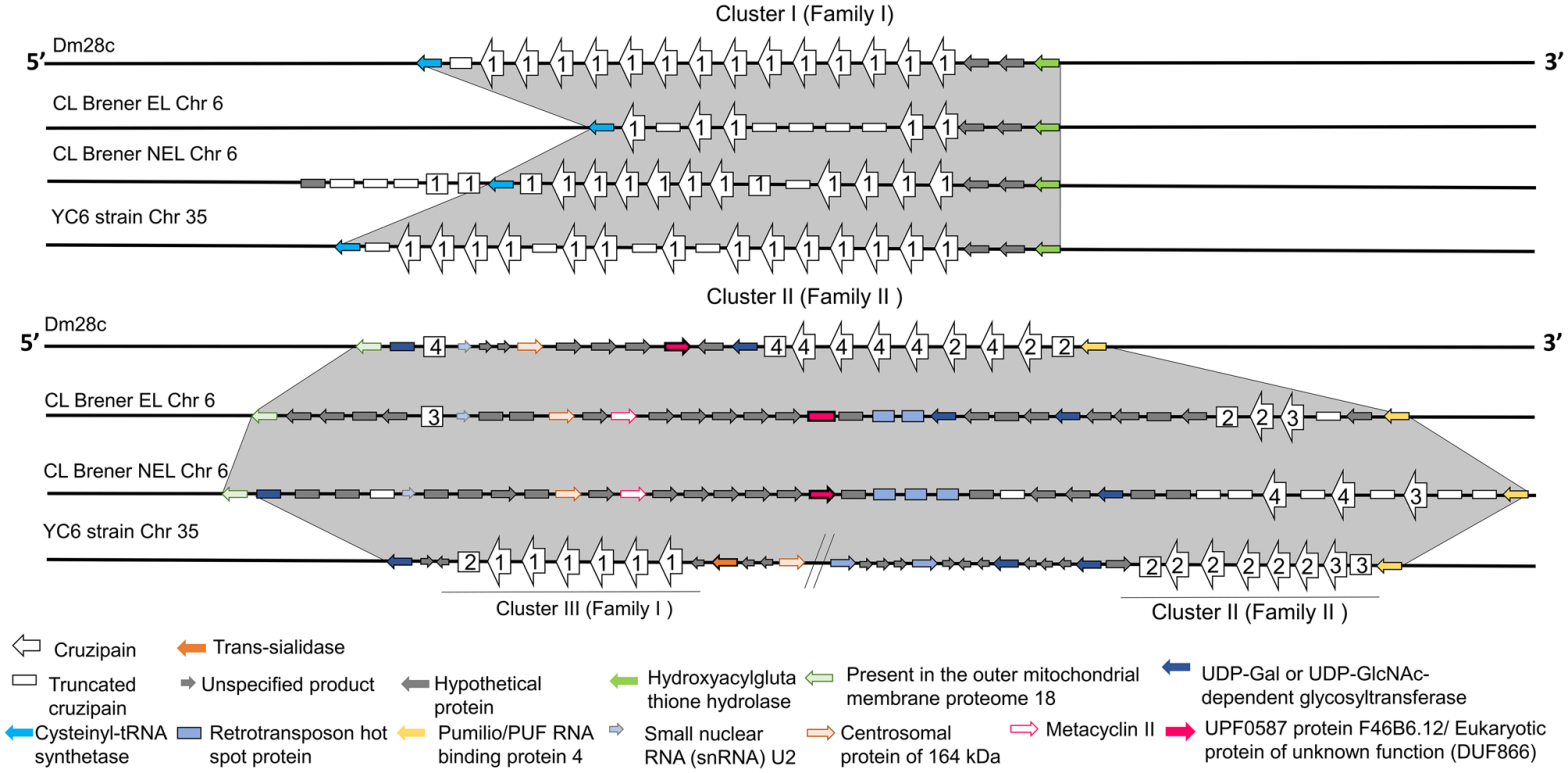
Representation of the syntenic cruzipain clusters in *T. cruzi* chromosomes. The homologous chromosomes of the Dm28c and YC6 strains are identical and represented once, while for the hybrid CL Brener strain, the two alleles corresponding to the Esmeraldo-like (EL) and non-Esmeraldo-like (NEL) haplotypes are represented separately. In each cluster, cruzipain copies are shown as white arrows, numbered according to the sub-type to which they belong, while truncated cruzipain copies are shown as white rectangles. The cruzipain clusters of the YC6 strain are found on chromosome 35 (Chr 35), and on chromosome 6 (Chr 6) for CL Brener. Genes are represented from 5’ to 3’ (genomic), and the arrows point in the predicted transcription direction. The genomic sequences were manually curated to search for full length and truncated copies that are represented here and might not match current annotation of cruzipain genes in TriTrypDB.

The distribution of the gene copies belonging to each proposed sub-type in the clusters of the three strains analyzed is depicted in Fig. 4. Cluster I/Family I is flanked by the same genes in Dm28c, YC6, and both CL Brener haplotypes. In contrast, Cluster II/Family II is more heterogeneous among the strains. While Dm28c and YC6 strains show a cluster with all cruzipain copies in tandem and one truncated copy further ahead in Dm28c, both alleles of CL Brener show a larger number of truncated copies, as well as single truncated copies spread nearby in the chromosome (white rectangles in Fig. 4). A detailed description of truncated copies is depicted in Supplementary Fig. 1). We could not classify the sub-types of most of the truncated copies in CL Brener because the truncations occurred before the regions containing the signature residues.

Part of the heterogeneity found in Family II is due to the fact that some sub-types are missing in each strain/haplotype. Dm28c contains interspaced czp2 and czp4; CL Brener EL, czp2 and czp3; CL Brener NEL, czp3 and czp4; YC6, czp2 and czp3; and TCC contains czp3 and czp4 (data not shown, sequences available at TriTrypDB). The sequences of the different sub-types within Family II share between 79 and 100% identity in the catalytic domain (Supplementary Table S5), and the third cluster, present in the YC6 strain, is hybrid, formed mainly by czp1 and a truncated copy of czp2.

**Figure 5:**
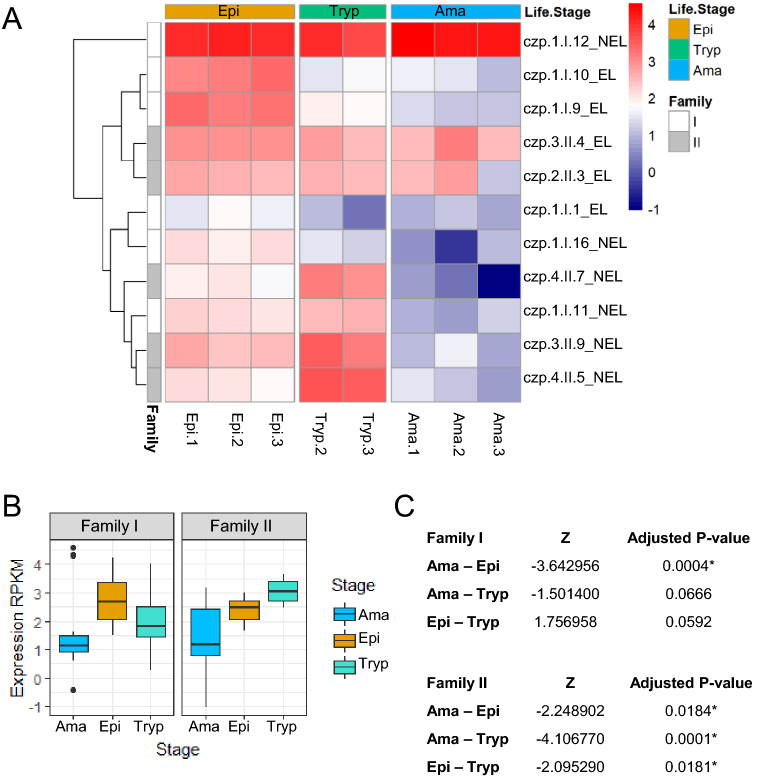
Expression profile of cruzipains in CL Brener. (**A**) Heatmap representing the expression values (log2RPKM) of 11 sequences coding cruzipains belonging to the two clusters in different stages of the life cycle of *T. cruzi* (Epi, epimastigotes; Tryp, trypomastigotes; Ama, amastigotes) in CL Brener Esmeraldo-like (EL) and non-Esmeraldo-like (NEL) haplotypes. Rows represent each sequence, and columns represent three library replicates derived from epimastigotes and intracellular amastigotes and two library replicates derived from trypomastigotes. The expression values are represented in a color grade scale, where red represents higher expression levels, and navy blue represents lower expression levels. The sequences were clustered according to their expression values. The *T. cruzi* life stages and cruzipain families are represented in different colors in the x and y-axis, respectively. (**B**) Boxplot representing the log2RPKM expression in Family I and Family II cruzipains, in the amastigote (Ama), epimastigote (Epi), and trypomastigote (Tryp) stages. (**C**) Z test statistics and adjusted *P* values (Benjamini-Hochberg) for each pair of comparisons of the Family I and Family II cruzipain gene expressions. *P values < 0.05.

In agreement with the differences regarding the encoded protein domains and the genomic organization, analyses of RNA-seq data showed that distinct expression patterns appear when Family members I and II are compared. Using the CL Brener RNA-seq data previously described by Belew et al.^50^ and Tavares et al.^51^, which evaluated global RNA levels present in epimastigotes, tissue culture-derived trypomastigotes, and intracellular amastigotes, we compared the transcription profiles of all cruzipain genes throughout the parasite life cycle. As shown in Fig. 5, the RNA-seq analysis corroborates previous studies^2,17^ showing increased expression of cruzipain belonging to Family I in epimastigotes, whereas expression of most copies belonging to Family II are up-regulated in trypomastigotes. In amastigotes, most cruzipain copies are expressed at lower levels, when compared with epimastigotes and trypomatigotes, with the exception of three copies: czp.2.II.3_EL and czp.3.II.4_EL from Family II; and czp1.I.12 from Family I. The mRNA levels of the cruzipain gene belonging to Family I (czp 1.I.12) was found to be highly expressed in all stages, being potentially the most expressed cruzipain in CL Brener *T. cruzi* strain.

### Differences in the active site of cruzipain sub-types

To investigate the structural differences among cruzipain sub-types, we modeled the catalytic domain of cruzipains from CL Brener, including at least one representative sequence from each sub-type. Due to the complete conservation of the active site within Family I and the existence of cruzain crystal structures corresponding to those sequences, only Family II representatives were modeled.

All modeled sequences showed total coverage and high identity (76% to 81%) to the cruzain template (PDB 1ME3). After energy minimization, validation of the models with QMEAN and ERRAT servers indicated their high quality, with no steric clashes or residues in the forbidden region of the Ramachandran plot (Supplementary Fig. S5, Supplementary Table S6). To generate models compatible with ligand binding, we modeled the proteins in the presence of the hydroxymethyl ketone inhibitor located in the PDB template. Additionally, to better evaluate the impact of residue differences on ligand binding, we determined the frequency of ligand interactions with each cruzain residue, considering all crystal structures available.

**Figure 6:**
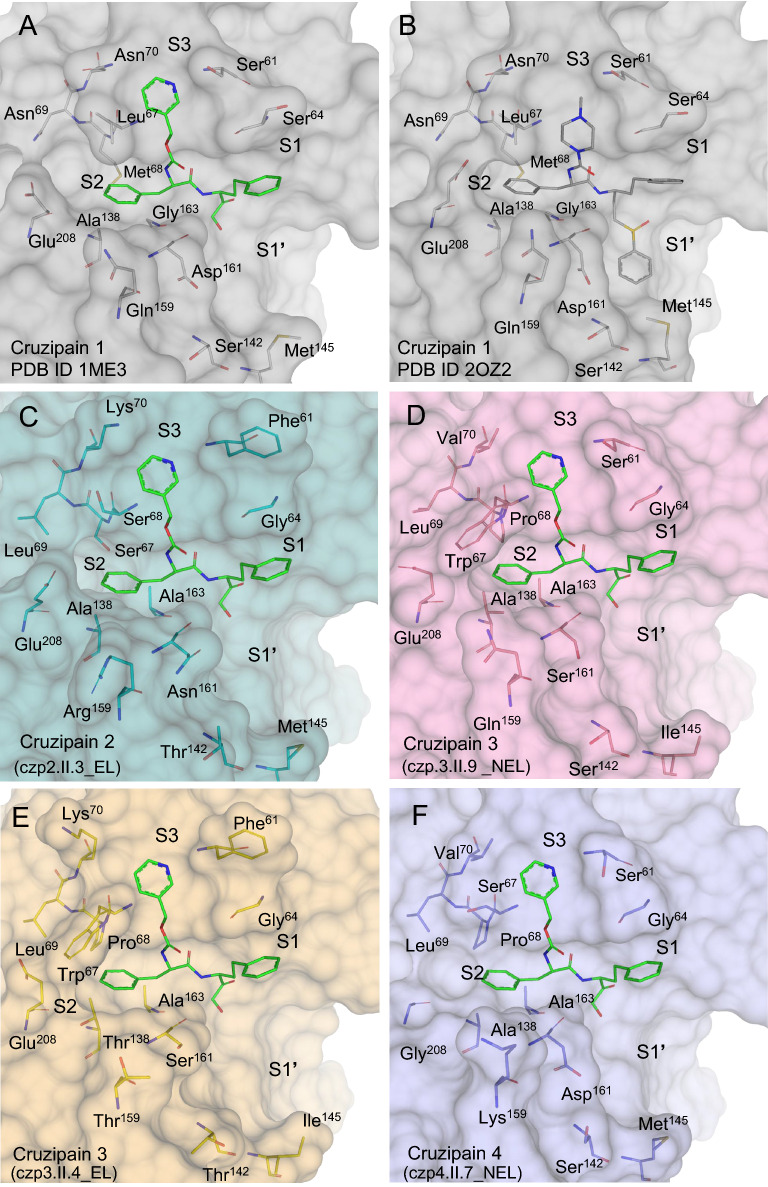
Mutations in cruzipain sequences impact the active site shape and the interactions between the enzyme and its ligands. (**A**) Cruzain (cruzipain 1 sub-type) from PDB 1ME3, in complex with a hydroxymethyl ketone inhibitor, the template for modeling the different sub-types. (**B**) Cruzain (cruzipain 1 sub-type) co-crystallized with K11777 (PDB 2OZ2), a preclinical drug candidate. C-F) Theoretical models for cruzipain 2 (czp.2.II.3_EL), 3 (czp.3.II.4_EL and czp.3.II.9_NEL), and 4 (czp.4.II.7_NEL) with the hydroxymethyl ketone inhibitor from PDB 1ME3. Protein structures are shown as surface and the residues that differ in at least one sub-type as sticks. S1, S1’, S2, and S3 indicate cruzipain subsites. Stick representations are colored by atom, with different colors for carbons in each cruzipain sequence: czp1 (gray), czp2 (light cyan), czp.3.II.9_NEL (pink), czp.3.II.7_EL (yellow), and czp4 (purple). Ligand carbons are represented in green (hydroxymethyl ketone) and gray (K11777).

A total of thirteen positions that contain residue variations in the cruzipain sequences were evaluated. Overall, the sub-types’ differences impact the shape, volume, and physical properties of the active site (Fig. 6). Even though these differences are distributed throughout the active site, there is an accumulation of amino acid substitutions within the S2 and S1’ subsites among the cruzipain sub-types. These regions are also more frequently involved in interactions with cruzain ligands (Table 2, Supplementary Table S7).

Two modifications occur in the S3 subsite: at residue 61, from Ser (czp1) to Phe (czp2 and czp.3.II.4_EL), and at residue 70, from Asn (czp1) to Lys (czp2 and czp.3.II.4_EL) or Val (czp.3.II.9_NEL and czp4). These changes affect the polarity and volume of S3, creating a better defined and positively charged cavity in czp2 and czp.3.II.4_NEL. Among the currently determined structures of cruzain-inhibitor complexes, Ser61 and Asn70 do not interact with any ligands.

The S2 subsite is the best-defined site in cruzain, playing an essential role in enzyme selectivity and ligand recognition^27,30^. This pocket concentrates most of the cruzain residues that interact with ligands in over 50% of the known crystal structures, including Gly66, which hydrogen bonds to 58% of the ligands, and several residues that form a hydrophobic pocket (Leu67, Met68, Ala133, Leu160) and establish hydrophobic interactions with at least 70% of the inhibitors (Supplementary Table S7). Among these highly interacting residues, we found differences in the cruzipain sub-types at positions 67, 68, 138, and 208 (Table 2). Positions 67 and 68 are critical to defining the shape of this pocket. Compared to czp1, which has Leu67 and Met68, czp2 has a bigger and more polar S2 due to Ser67 and Ser68. On the other hand, the two czp3 have a smaller and more solvent-exposed S2 than members of all other cruzipain sub-types since they contain Trp67 and Pro68; in the case of czp.3.II.4_EL, Ala138 is also replaced by a bulkier residue (Thr). The czp4 sub-type has Ser67 and Pro68 (Fig. 6E). The most noticeable difference found in czp4 is the replacement of Glu208 by a glycine, making the S2 subsite shallow and open. The Glu208 at the bottom of the pocket of this cruzipain subsite is essential for accommodating either hydrophobic or positively charged groups by cruzipain 1 in this region^30^.

The S1 subsite is similar in all cruzipain sub-types, and the only modification found in this region is the replacement of czp1 Ser64 by Gly64 in all other cruzipains. The modifications at S1’ impact the charge and volume of this subsite. Before the catalytic histidine, czp1 and czp4 have an Asp161, which frequently performs hydrogen bonds with ligands in cruzain crystals and donates a negative character to the S1’ pocket (Table 2). This position is occupied by polar neutral residues (Asn in czp2 and Ser in czp3) in other sub-types. A conservative substitution is found in position 142, containing either Ser (czp1, czp4, and czp.3.II.9_NEL) or Thr (czp2 and czp.3.II.4_EL). At the bottom of this pocket, Met145 interacts with 29% of cruzain crystallographic ligands, and Ile replaces it in czp3.

Additionally, we observed differences in position 159, located between the S2 and S1’ sites. Czp1 and czp3 have polar uncharged residues (Gln159 in czp 1 and czp.3.II.9_NEL, Thr159 in czp.3.II.4_EL), while in czp2 and czp4 there are basic residues, Arg159 and Lys159 respectively, in this position (Fig. 6).

## Discussion

The existence of structural divergence between at least two cruzipain sub-types, cruzipain 1 and cruzipain 2, was already well established, with consequences to their kinetic properties^17,18^. However, the exact genomic organization, copy number, and heterogeneity of cruzipains were still uncertain. To better characterize cruzipain sub-types in *T. cruzi*, we combined genomic, phylogenetic, transcriptomic, and structural analysis to study cruzipain sequences from three parasite strains belonging to three major lineages found in the parasite population: *T. cruzi* I (Dm28c), *T. cruzi* II (YC6), and the hybrid CL Brener strain belonging to *T. cruzi* VI. We propose the division of cruzipains into two families, with Family II further subdivided into three sub-types. Czp1, the archetype of the classical cruzipain/cruzain that has been extensively studied, was identified in all parasite strains analyzed, and they are virtually identical. In the CL Brener and Dm28c genomes, the czp1 sequences are clustered in a single genomic location, referred to as Cluster I. This cruzipain sub-type has been previously reported in several *T. cruzi* strains^20^. In contrast, czp1 gene copies in the YC6 strain are distributed over two independent clusters.

Based on genomic location, phylogenetic analysis, and expression pattern during the lifecycle, the cruzipains can be divided into two families, with sequences from Family II being more diverse than sequences from Family I. These results suggest that while the expansion of Family I with identical copies of czp1 may have arisen to increase expression levels of this sub-type, the expansion of Family II may be associated with the increased enzymatic repertoire of the parasite. In previous work that described sequence divergence among cruzipains of Dm28c^16^, the combinations of specific primers and restriction digestion of PCR fragments using genomic DNA from Dm28c and Y strain led to the proposal that further genetic diversity occurred in cruzipains from different *T. cruzi* strains. In agreement with this hypothesis, we could not find all the Family II sub-types in all the strains we analyzed. Furthermore, the comparison of cruzipain sub-types from the different strains suggests that isoenzymes exist within each sub-type (Fig. 3C).

Our data suggest that parasite strains have adapted to acquire specific cruzipain sub-types for distinct biological functions if one assumes that the four cruzipain sub-types display distinct kinetic properties and/or specificities. One example is the usage of cruzipain 1 and 2 by Dm28c trypomastigotes to process human kininogen and generate bioactive kinins required for host cell invasion^6^. Beyond its role in invasion, the generation of kinins by cruzipains impacts the course of adaptive immunity^9,31^, tissue parasitism, and pathology^32^. Studies using recombinant cruzipain 2 originated from a Dm28c gene revealed increased stability at alkaline pH, distinct substrate specificity, and lower affinity for mammalian cysteine peptidase inhibitors^17,19^. The expansion of the copy number of czp2 in the YC6 strain, compared to Dm28c and CL Brener (Fig. 4), may offer the means for increased expression of this sub-type, if required.

The third cluster of czp1 copies found solely in YC6 is intriguing and raises questions as to whether it is also present in other TcII strains, and if there is any adaptative advantage for its retention by YC6 strain parasites. By comparing the YC6 TriTrypDB syntenic information with that of CL Brener EL we could not find a third cluster in the Esmeraldo haplotype. However, we will be able to address this issue further as more genomic data become available. In the absence of available genomes from additional strains containing a third cruzipain cluster, it is difficult to answer whether YC6 is the exception or the rule. Either way, selective pressure may be taking place to drive the maintenance of this third cluster by YC6 strain parasites.

We found a variety of truncated cruzipain genes due to frameshifts at different positions that should mostly result in inactive protease. Some of those truncations may not be real and result from assembly problems. Artificial truncated cruzipain genes could be a consequence of assembly limitations imposed by repetitive sequences including multigene families such as cruzipains, a largely recognized challenge faced during *T. cruzi* genome assembly (revised by^33^). Another possibility related to the biology of the parasite is that truncated sequences could be the result of recombination events, a known mechanism to generate sequence variability in *T. cruzi* multigene families, but that may also generate pseudogenes^34,35^. Gene conversion is also a possibility, considering their homology and the short distances between copies in a cluster, and could have contributed both to the expansion of the family and to the generation of variability. In Cluster II of Dm28c and YC6, for example, truncated sequences were found solely at the extremities of the tandems, which could be scars from genomic rearrangement that occurred after a break in a pre-existing tandem. In Dm28c, the existence of a truncated single czp4 copy further down the chromosome, flanked by a truncated UDP-Gal gene, together with an intergenic region which is identical to that found between the last truncated copy of the tandem in Cluster II and the downstream full-length UDP-Gal gene, could be an example of the duplication at the extremity of the cluster, followed by a break. Interestingly, we found that intergenic regions between copies of the same cluster were nearly identical to each other when the different strains were compared. In contrast, there was less similarity between the intergenic regions located in Cluster I with those located in Cluster II within the same strain. Indeed, we found a deletion of 62 base pairs in intergenic regions in Cluster II in all three strains, when comparing to the respective intergenic regions of Cluster I. This raises the tempting possibility that the cruzipain genes might have diverged and separated in different clusters very early in *T. cruzi*, before the different DTUs diverged, followed by further expansion of each cluster by duplication events leading to heterogeneous Cluster lengths and cruzipain subtypes.

Based on the RNA-seq data for the CL Brener strain and in agreement with other studies, trypomastigotes were found to have higher expression levels of Family II cruzipains. At present, we do not know how this stage-regulation takes place. However, the shorter intergenic region between Family II gene copies might contribute to regulate stage-specific expression. Previous quantitative studies have shown lower overall cruzipain mRNA levels in trypomastigotes as compared to epimastigotes^21^, although they did not discriminate between cruzipain sub-types. Qualitative studies identified mRNA for Family II cruzipains in Dm28c trypomastigotes and amastigotes, while only Family I cruzipain mRNA could be identified in epimastigotes^16^. This repertoire switch might be related to distinct biological functions displayed by cruzipain sub-types at the infective stage. The differential expression of genes belonging to distinct sub-types may be necessary for the parasite to meet the different metabolic demands in each life stage. The varied sub-types may not be redundant and cooperate to fulfill the parasite’s changing needs. That is the case, for example, of mammalian lysosomal cysteine cathepsins, which retain distinct substrate specificities. Another advantage of keeping different copies of such an important gene could be the generation of resistance to natural inhibitors. For example, cruzipain 2 shows increased resistance to inhibition by kininogen and cystatins, while cruzipain 1 is more susceptible to those inhibitors^19^.

The more significant variation found in the catalytic domain than in other regions of the cruzipain sub-types, namely the prodomain and C-terminal regions, agrees with previous reports^16^ and correlates with the expression of different arrays of cruzipain sub-types at different life stages. Among the substitutions found in the catalytic domain, thirteen positions are located within the active site, in the region comprising the S3-S1’ pockets, and include nonconservative substitutions. As expected, based on these differences, modeling of the four cruzipain sub-types revealed significant changes in the volume, hydrophobicity, and shape of their active sites. Modeled sequences included those expressed in all life cycle stages (czp.3.II.4_EL and czp.2.II.3_EL), and those more highly expressed in trypomastigotes (czp.3.II.9_NEL and czp.4.II.7_NEL). In the S3 subsite, replacing Ser61 with Phe allows hydrophobic contacts and π-stacking interactions, and modification from Asn70 to Lys adds a positive charge to the pocket, opening the possibility to explore new types of interactions. In the S1’ subsite, replacement of Asp161 in cruzain for Ser in Dm28c cruzipain 2 has been associated with loss of recognition of Arg in P1′^18^. Our results show that position 161 may also contain serine (in CL Brener czp3) or asparagine, which should affect the ability to recognize positively charged groups in S1’ due to the absence of the protein negative charge in this subsite. Residues Asp161 and Met145, for which substitutions are also found among the cruzipain sub-types, frequently interact with cruzain ligands in crystallography complexes. A study with a series of vinyl sulfones varying the P1′ substituents has also reported these two positions to be key for recognition in S1′^36^.

Despite the substitutions observed throughout all subsites analyzed, interestingly, most modifications are found in S2, the only well-defined pocket and a key determinant of specificity in papain-like cysteine proteases^26,37^. Six of the variable positions are in this pocket, including Leu67, Met68, and Ala138, which interact with at least 70% of all ligands present in known cruzain structures (Table 2 and Supplementary Table S7). Interactions with Leu67 have also been described as an important characteristic, discriminating potent from less active vinyl sulfone inhibitors^38^. Therefore, nonconservative substitutions in these positions might have an impact on the inhibition profile of known cruzain inhibitors towards other cruzipain sub-types. In the case of cruzipain 2, it has been already shown that the amino acid differences, when compared to cruzipain 1, affect substrate specificity and susceptibility to inhibitors^17,18^. For other sub-types, the effect of the substitutions is still to be experimentally verified, but it is likely to occur, considering our current knowledge on ligand recognition by cysteine proteases. Most strikingly is the substitution in residue 208 encountered in czp4. It is known that Glu208 is a determinant of cruzain specificity and allows recognition of both positively charged and hydrophobic groups in the S2 pocket, donating cathepsin B-like characteristics to cruzain^30^. *Tbr*CatL (or rhodesain), the orthologous enzyme from *T. brucei*, has an alanine structurally aligned with the cruzain Glu208. That is the only difference in the S2 pocket of these parasitic enzymes, resulting in a more open pocket. While several classes of inhibitors binding both to cruzain and *Tbr*CatL have been described^12^, studies with a series of benzimidazole inhibitors provide an example of different structure–activity relationships for the two enzymes^39–41^.

Based on these active site comparisons, two points are worth highlighting. First, the conservation of several residues in the active site of different cruzipain sub-types suggests that it is possible to develop inhibitors that hit enzymes of all the four sub-types. On the other hand, there are enough substitutions in this region to suggest that inhibitors will have different affinities or even reach selectivity among the cruzipain sub-types. Throughout the literature, several studies have described cruzain inhibitors that are not effective against *T. cruzi* or discrepancies between the structure–activity relationships obtained for a series of compounds against cruzain and the parasite^40,42,43^. These differences are partially explained by the different chemical properties of the compounds, affecting features like membrane permeability, chemical stability, and metabolism. However, the inhibition profile against multiple cruzipain sub-types might also play an essential role in defining the correlation between enzyme inhibition and trypanocidal activity. Studies reporting compounds with activity against the parasite in concentrations lower than their IC50 against cruzain^44,45^, or even with efficacy in mouse models despite modest cruzain inhibition^46^, have also put forth the hypothesis that activity against other cruzipain sub-types could explain these results.

For decades, drug design projects have been focused on cruzain, the recombinant form of czp1, expressed in all the parasite life cycle forms. However, epimastigotes express much higher levels of czp1, while amastigotes and trypomastigotes express higher levels of Family II cruzipains. Considering that antichagasic drugs must be effective in the mammalian stages, one should look for inhibitors that are active against both czp1 and Family II cruzipains. Furthermore, the different expression patterns of the cruzipain sub-types within the different *T. cruzi* strains should be considered, as czp2 is expressed in Dm28c amastigotes and trypomastigotes, while czp3 and czp4 are expressed in CL Brener trypomastigotes. It would be of great importance to drug discovery projects to biochemically characterize these sub-types and evaluate their inhibition profile by known cruzain inhibitors in order to support the development of new inhibitor series. Family II sequences are now under investigation as possible new drug targets to treat Chagas disease.

## Material and methods

### Genomic characterization

We searched the TriTrypDB for annotated cruzipain sequences in CL Brener, CL Brener Esmeraldo-like (EL), CL Brener non-Esmeraldo-like (NEL), and YC6 strain using procruzipain (GenBank: AAB41119.1) and cruzain (PDB: 3KKU) sequences as queries.

We obtained the genomic sequences from CL Brener (Coqueiro-dos-Santos et al., *In preparation*) and Dm28c^22,23^ strains using PacBio^47^ and Nanopore (gently provided by Carlos Robello, Institut Pasteur, Uruguay) sequencing technologies; YC6 strain sequences^24^ are all from PacBio^47^ and Illumina HiSeq platform. The assembly of CL Brener genome will be described elsewhere (Coqueiro-dos-Santos et al., *In preparation*). CL Brener sequences were deposited in GenBank under the numbers MZ087216 to MZ087262, and the Dm28c ones, can be found in the BioProject PRJNA727253, in the NCBI database. We analyzed these genomic sequences by BLAST to localize the cruzipain sequences in both genomes. We manually annotated the target sequences to establish the localization of all cruzipain sequences and their flanking genes. To do so, we compared our genomic sequences with annotated *T. cruzi* genomes at TriTrypDB. We compared Nanopore derived Dm28c sequences with Dm28c 2018 and Dm28c 2017, and CL Brener sequences with CL Brener EL and CL Brener NEL.

The intergenic regions between any two tandem cruzipain copies, from CL Brener, Dm28c and YC6 strains, were aligned with Clustal Omega^48^. This region starts with the stop codon (TGA) from one copy and ends with the ATG codon from the next cruzipain copy. For truncated copies, we considered the start and stop codons corresponding to the expected original full-length sequences. To do so, we first aligned all the intergenic regions between cruzipains copies of all strains and established these codons based on this alignment.

### Phylogenetic analysis

We aligned all cruzipain full-length protein sequences from CL Brener, Dm28c, and YC6 with Clustal Omega^48^ and constructed phylogenetic trees with MEGA-X^49^, based on the catalytic domain sequences. We constructed a Maximum Likelihood tree and a Neighbor-Joining tree; in both, we analyzed 1000 replicates for the bootstrap calculation and used the Jones-Taylor-Thornton (JTT) matrix-based method^50^ to compute the evolutionary distances. We used the rhodesain gene as root.

### RNA-seq data access, quality control, mapping, and expression profile of cruzipains

To create the expression profile of cruzipains in *T. cruzi*’s life cycle different forms (epimastigote, trypomastigote, and amastigote)*,* we obtained the RNA-seq data from CL Brener^51,52^ bioprojects PRJNA389925 and PRJNA389926, respectively, deposited in NCBI database. Read quality was analyzed with FastQC (http://www.bioinformatics.babraham.ac.uk/ projects/fastqc/) and low-quality reads (Per Sequence Quality Scores < 25 and read length < 50) were removed with Trimmomatic^53^. Both paired and unpaired remnant reads were mapped to the CL Brener genome (Coqueiro-dos-Santos et al., *In preparation*) with BWA-MEM61. We selected the mapped reads presenting mapping quality above 30 with Samtools^54^ and counted with featureCounts^55^, normalizing the counts by the total number of mapped reads in each sample and by the gene length to obtain the reads per kilobase per million (RPKM). Cruzipain sequences with low expression (rowSums(cpm > 2) >  = 2) or truncated sequences/pseudogenes were filtered out. The expression profile of remnant cruzipains were displayed by a heatmap of log2(RPKM) of each sequence for all samples. Log2(RPKM) values from the czp I (czp.1.I.10_EL, czp.1.I.9_EL, czp.1.I.1_EL, czp.1.I.16_NEL, czp.1.I.12_NEL, czp.1.I.11_NEL) and czp II (czp.3.II.4_EL, czp.2.II.3_EL, czp.3.II.9_NEL, czp.4.II.7_NEL, czp.4.II.5_NEL) genes were compared using Dunn’s test of multiple comparison using rank sums, with the R library dunn.test (https://cran.r-project.org/web/packages/dunn.test/index.html) to compare alterations in the gene expression of cruzipains from Families I and II. Significance was assumed if the corrected *P* value (Benjamini-Hochberg) was lower than 0.05.

### Comparative modeling

We submitted to comparative modeling on Modeller v9.23^56^ the cruzipain sequences czp.2.II.3_EL, czp.3.II.4_EL, czp.3.II.9_NEL, and czp.4.II.7_NEL. We employed the cruzain structure PDB ID 1ME3^57^ as a template and transferred the hydroxymethyl ketone inhibitor’s coordinates located in this structure to the models. We selected the top five models built for each sequence, based on the DOPE score, for model assessment in Molprobity, ERRAT, and QMEAN servers. We minimized the best-validated model, applying 20 steps of the conjugate gradient method and fixing the catalytic dyad (Cys25 and His162) to preserve the conserved conformation observed in cruzain crystals. We edited the Gln19 rotamer on Chimera to maintain the rotamer compatible with its function in the oxyanion hole and conserved among cruzain crystal structures. After minimizing each structure, we repeated the evaluation described to validate the final model for each sequence.

### Analysis of protein–ligand interactions

We analyzed twenty-four crystal structures of cruzain in complex with inhibitors, available in the Protein Data Bank in May 2020, using nAPOLI^58^. We detected the interactions based on the default distance cutoffs set in this tool: hydrogen bonds minimum angle of 120º and acceptor–donor distance between 2.5 and 3.9 Å; aromatic stacking between 2 and 4 Å; and hydrophobic contacts between 2.0 and 4.5 Å. We considered the following PDB structures in this analysis: 1AIM, 1EWL, 1EWM, 1EWO, 1EWP, 1F29, 1F2A, 1F2B, 1F2C, 1ME3, 1ME4, 1U9Q, 2AIM, 2OZ2, 3HD3, 3I06, 3IUT, 3KKU, 3LXS, 4KLB, 4PI3, 4W5B, 4XUI, 6O2X.

## Supplementary Information

Supplementary Information 1.

Supplementary Information 2.

## Data Availability

The accession codes and links for the gene sequences, and PDB codes employed in our analyses, are included in the Methods section.

## Abbreviations

EL: Esmeraldo-like
NEL: Non-Esmeraldo-like
Czp: Cruzipain
Chr: Chromosome
Epi: Epimastigotes
Tryp: Trypomastigotes
Ama: Amastigotes
