# Supplementary material for: The gene repertoire of the main cysteine protease of Trypanosoma cruzi, cruzipain, reveals four sub-types with distinct active sites
Source: Sci Rep. 2021 Sep 14;11:18231. doi: 10.1038/s41598-021-97490-2 (PMC8440672; doi:10.1038/s41598-021-97490-2)
Supplement: Supplementary file 2 — Supplementary Information 2. [file 41598_2021_97490_MOESM2_ESM.pdf]

Supporting Information

The gene repertoire of the main cysteine protease of *Trypanosoma cruzi*, cruzipain, reveals four sub-types with distinct active sites

Viviane Corrêa Santos[1,#], Antonio Edson Rocha Oliveira[1,2,#], Augusto César Broilo Campos[1], João Luís Reis-Cunha[3,4], Daniella Castanheira Bartholomeu[3], Santuza Maria Ribeiro Teixeira[1], Ana Paula C. A. Lima[5,*], Rafaela Salgado Ferreira[1,*]

[1] Departamento de Bioquímica e Imunologia, Universidade Federal de Minas Gerais, Belo Horizonte, MG, Brazil.

[2] Departamento de análises clínicas e toxicológicas, Faculdade de Ciências Farmacêuticas, Universidade de São Paulo, São Paulo, Brazil.

[3] Departamento de Parasitologia, Universidade Federal de Minas Gerais, Belo Horizonte, MG, Brazil.

[4] Departamento de Medicina Veterinária Preventiva, Escola de Veterinária, Universidade Federal de Minas Gerais, Belo Horizonte, Minas Gerais, Brazil

[5] Instituto de Biofísica Carlos Chagas Filho, Universidade Federal do Rio de Janeiro, Rio de Janeiro, RJ, Brazil.

[#]These authors contributed equally to this work.

Table S1. **Identity matrix for complete cruzipain protein sequences from Dm28c, CL Brener and YC6 *Trypanosoma cruzi* strains.** Provided as a separate spreadsheet.

Table S2. **Identity matrix for the intergenic regions of cruzipains from Dm28c, CL Brener and YC6 *Trypanosoma cruzi* strains.** Provided as a separate spreadsheet.

Table S3. **Identity matrix for the preprodomain protein sequences of cruzipains from Dm28c, CL Brener and YC6 *Trypanosoma cruzi* strains.** Provided as a separate spreadsheet.

Table S4. **Identity matrix for the C-terminal protein sequences of cruzipains from Dm28c, CL Brener and YC6 *Trypanosoma cruzi* strains.** Provided as a separate spreadsheet.

Table S5. **Identity matrix for the catalytic domain protein sequences of cruzipains from Dm28c, CL Brener and YC6 *Trypanosoma cruzi* strains.** Provided as a separate spreadsheet.

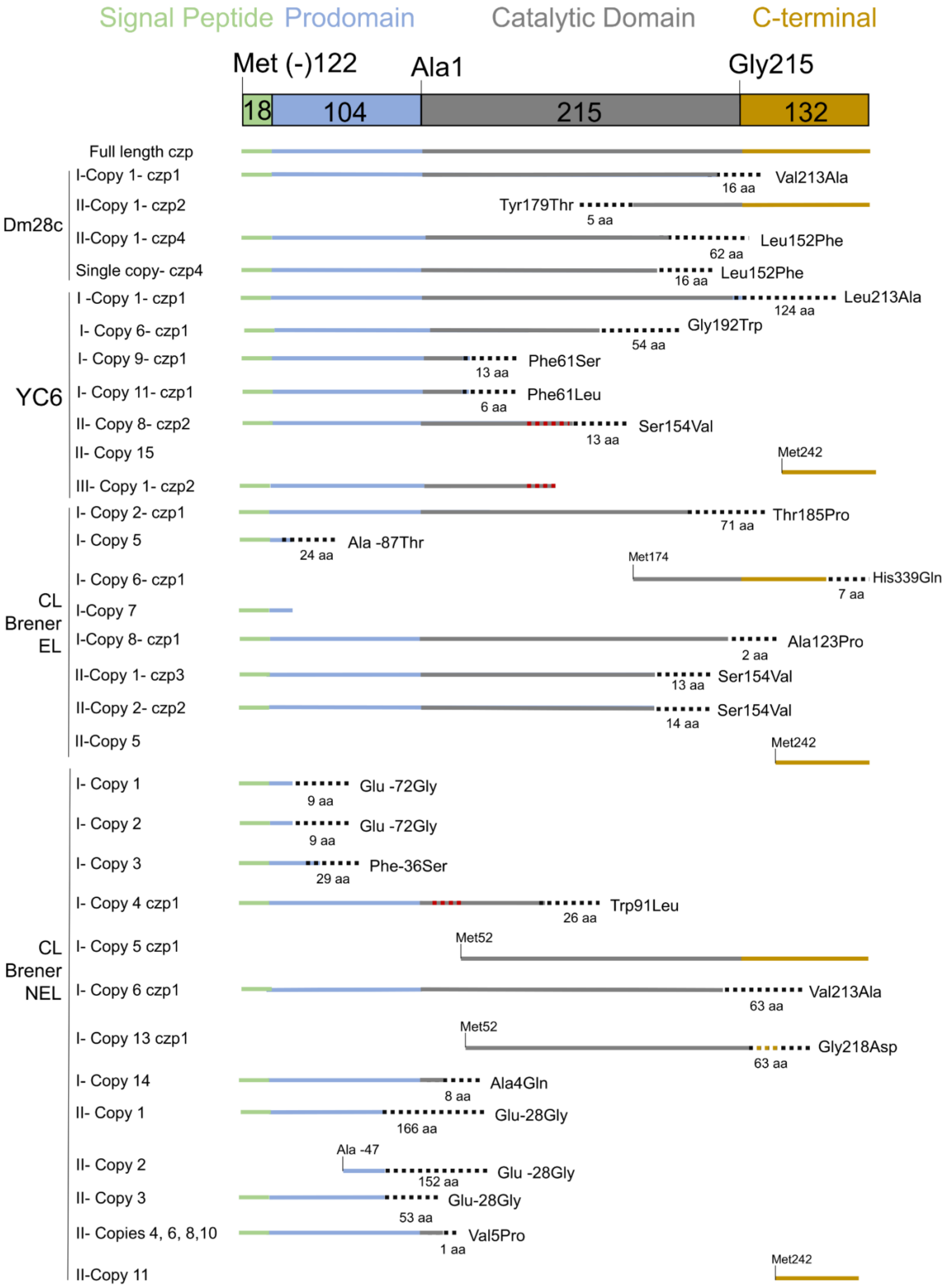

Signal Peptide
Prodomain
Catalytic Domain
C-terminal
Met (-)122
Ala1
Gly215
18
104
215
132
Full length czp
Dm28c
I-Copy 1- czp1
Val213Ala
16 aa
II-Copy 1- czp2
Tyr179Thr
5 aa
II-Copy 1- czp4
Leu152Phe
62 aa
Single copy- czp4
Leu152Phe
16 aa
YC6
I -Copy 1- czp1
Leu213Ala
124 aa
I- Copy 6- czp1
Gly192Trp
54 aa
I- Copy 9- czp1
Phe61Ser
13 aa
I- Copy 11- czp1
Phe61Leu
6 aa
II- Copy 8- czp2
Ser154Val
13 aa
II- Copy 15
Met242
III- Copy 1- czp2
CL Brener EL
I- Copy 2- czp1
Thr185Pro
71 aa
I- Copy 5
Ala -87Thr
24 aa
I- Copy 6- czp1
Met174
His339Gln
7 aa
I-Copy 7
I-Copy 8- czp1
Ala123Pro
2 aa
II-Copy 1- czp3
Ser154Val
13 aa
II-Copy 2- czp2
Ser154Val
14 aa
II-Copy 5
Met242
CL Brener NEL
I- Copy 1
Glu -72Gly
9 aa
I- Copy 2
Glu -72Gly
9 aa
I- Copy 3
Phe-36Ser
29 aa
I- Copy 4 czp1
Trp91Leu
26 aa
I- Copy 5 czp1
Met52
I- Copy 6 czp1
Val213Ala
63 aa
I- Copy 13 czp1
Met52
Gly218Asp
63 aa
I- Copy 14
Ala4Gln
8 aa
II- Copy 1
Glu-28Gly
166 aa
II- Copy 2
Ala -47
Glu -28Gly
152 aa
II- Copy 3
Glu-28Gly
53 aa
II- Copies 4, 6, 8,10
Val5Pro
1 aa
II-Copy 11
Met242

Figure S1. **Schematic representation of truncated cruzipain genes.** The schematic representation of full-length cruzipain is depicted at the top and color-coded for each domain: green, signal peptide (pre-region = 18 amino acid residues); blue, prodomain = 104 amino acid residues; grey, catalytic (central) domain = 215 amino acid residues; and gold, C-terminal extension = 130 amino acid residues. The parasite strain is indicated at the left of the figure. Sequences are named according to the following scheme: the gene cluster is indicated in roman, followed by the gene copy number according to its position in the cluster, followed by sub-type classification. The protein domains are represented according to the color codes, and the positions where frameshifts occurred are shown. The black dotted lines indicate amino acid residues predicted after the frameshift (and unrelated to cruzipain) until the stop codon is reached, resulting in chimeric protein. The number of unrelated amino acids represented by the black dotted lines for each truncated cruzipain is indicated below the line, while the residue where the frameshift occurs is indicated next to each line. The red dotted lines represent amino acid substitutions when aligned to the corresponding sub-type of the same strain. The gold dotted lines indicate regions in the middle of the frameshift sharing similarity with cruzipain sequence.

```
CLUSTAL O(1.2.4) multiple sequence alignment

I_c8_7_EL          TGAGGGCACTCTTTGTTTCTGATGCTGCCTTTTCTCTCATTTTACTTTTTTATTATTATT   60
I_c16_15_YC6       TGAGGGCACACTTTGTTTCTGATGCTGCCTTTTCTCTCATTTTACT-TTTTATTATTATT   59
I_c5_4_EL          TGAGGGCACTCTTTGTTTCTGATGCTGCCTTTTCTCTCATTTTACTTTTTTATTATTATT   60
I_c18_17_YC6       TGAGGGCACTCTTTGTTTCTGATGCTGCCTTTTCTCTCATTTTACTTTTTTATTATTATT   60
I_c17_16_YC6       TGAGGGCACACTTTGTTTCTGATGCTGCCTTTTCTCTCATTTTACTTTTTTATTATTATT   60
I_c10_9_EL         TGAGGGCACTCTTTGTTTCTGATGCTGCCTTTTCTCTCATTTTACTTTTTTATTATTATT   60
I_c4_3_EL          TGAGGGCACTCTTTGTTTCTGATGCTGCCTTTTCTCTCATTTTACTTTTTTATTATTATT   60
I_c3_2_EL          TGAGGGCACTCTTTGTTTCTGATGCTGCCTTTTCTCTCATTTTACTTTTTTATTATTATT   60
I_c2_1_EL          TGAGGGCACTCTTTGTTTCTGATGCTGCCTTTTCTCTCATTTTACTTTTTTATTATTATT   60
I_c2-1_YC6         TGAGGGCACTCTTTGTTTCTGATGCTGCCTTTTCTCTCATTTTACTTTTTTATTATTATT   60
I_c3_2_YC6         TGAGGGCACTCTTTGTTTCTGATGCTGCCTTTTCTCTCATTTTACTTTTTTATTATTATT   60
I_c4_3_YC6         TGAGGGCACTCTTTGTTTCTGATGCTGCCTTTTCTCTCATTTTACTTTTTTATTATTATT   60
I_c5_4_YC6         TGAGGGCACTCTTTGTTTCTGATGCTGCCTTTTCTCTCATTTTACTTTTTTATTATTATT   60
I_c6_5_YC6         TGAGGGCACTCTTTGTTTCTGATGCTGCCTTTTCTCTCATTTTACTTTTTTATTATTATT   60
I_Yc7_6_YC6        TGAGGGCACTCTTTGTTTCTGATGCTGCCTTTTCTCTCATTTTACTTTTTTATTATTATT   60
I_c8_7_YC6         TGAGGGCACTCTTTGTTTCTGATGCTGCCTTTTCTCTCATTTTACTTTTTTATTATTATT   60
I_c9_8_YC6         TGAGGGCACTCTTTGTTTCTGATGCTGCCTTTTCTCTCATTTTACTTTTTTATTATTATT   60
I_c10_9_YC6        TGAGGGCACTCTTTGTTTCTGATGCTGCCTTTTCTCTCATTTTACTTTTTTATTATTATT   60
I_c11_10_YC6       TGAGGGCACTCTTTGTTTCTGATGCTGCCTTTTCTCTCATTTTACTTTTTTATTATTATT   60
I_c12_11_YC6       TGAGGGCACTCTTTGTTTCTGATGCTGCCTTTTCTCTCATTTTACTTTTTTATTATTATT   60
I_c13_12_YC6       TGAGGGCACTCTTTGTTTCTGATGCTGCCTTTTCTCTCATTTTACTTTTTTATTATTATT   60
I_c14_13_YC6       TGAGGGCACTCTTTGTTTCTGATGCTGCCTTTTCTCTCATTTTACTTTTTTATTATTATT   60
I_c15_14_YC6       TGAGGGCACTCTTTGTTTCTGATGCTGCCTTTTCTCTCATTTTACTTTTTTATTATTATT   60
III_c2_1_YC6       TGAGGGCACTCTTTGTTTCTGATGCTGCCTTTTCTCTCATTTTACTTTTTTATTATTATT   60
III_c3_2_YC6       TGAGGGCACTCTTTGTTTCTGATGCTGCCTTTTCTCTCATTTTACTTTTTTATTATTATT   60
III_c4_3_YC6       TGAGGGCACTCTTTGTTTCTGATGCTGCCTTTTCTCTCATTTTACTTTTTTATTATTATT   60
III_c5_4_YC6       TGAGGGCACTCTTTGTTTCTGATGCTGCCTTTTCTCTCATTTTACTTTTTTATTATTATT   60
III_c6_5_YC6       TGAGGGCACTCTTTGTTTCTGATGCTGCCTTTTCTCTCATTTTACTTTTTTATTATTATT   60
III_c7_6_YC6       TGAGGGCACTCTTTGTTTCTGATGCTGCCTTTTCTCTCATTTTACTTTTTTATTATTATT   60
I_c9_8_EL          TGAGGGCACTCTTTGTTTCTGATGCTGCCTTTTCTCTCATTTTACTTTTTTATTATTATT   60
I_c14_13_NEL       TGAGGGCACTCTTTGTTTCTGATGCTGCC-TTTTTCTCATTTTACTTTTTTATTATTATT   59
I_c5_4_NEL         TGAGGGCACTCTTTGTTTCTGATGCTGCCTTTTTTCTCATTTTACTTTTTTATTATTATT   60
I_c7_6_NEL         TGAGGGCACTCTTTGTTTCTGATGCTGCCTTTTTTCTCATTTTACTTTTTTATTATTATT   60
I_c2_1_dm28c       TGAGGGCACTCTTTGTTTCTGATGCTGCCTTTTTTCTCATTTTACTTTTTTATTATTATT   60
I_c3_2_dm28c       TGAGGGCACTCTTTGTTTCTGATGCTGCCTTTTTTCTCATTTTACTTTTTTATTATTATT   60
I_c4_3_dm28c       TGAGGGCACTCTTTGTTTCTGATGCTGCCTTTTTTCTCATTTTACTTTTTTATTATTATT   60
I_c5_4_dm28c       TGAGGGCACTCTTTGTTTCTGATGCTGCCTTTTTTCTCATTTTACTTTTTTATTATTATT   60
I_c6_5_dm28c       TGAGGGCACTCTTTGTTTCTGATGCTGCCTTTTTTCTCATTTTACTTTTTTATTATTATT   60
I_c7_6_dm28c       TGAGGGCACTCTTTGTTTCTGATGCTGCCTTTTTTCTCATTTTACTTTTTTATTATTATT   60
I_c8_7_dm28c       TGAGGGCACTCTTTGTTTCTGATGCTGCCTTTTTTCTCATTTTACTTTTTTATTATTATT   60
I_c9_8_dm28c       TGAGGGCACTCTTTGTTTCTGATGCTGCCTTTTTTCTCATTTTACTTTTTTATTATTATT   60
I_c10_9_dm28c      TGAGGGCACTCTTTGTTTCTGATGCTGCCTTTTTTCTCATTTTACTTTTTTATTATTATT   60
I_c11_10_dm28c     TGAGGGCACTCTTTGTTTCTGATGCTGCCTTTTTTCTCATTTTACTTTTTTATTATTATT   60
I_c12_11_dm28c     TGAGGGCACTCTTTGTTTCTGATGCTGCCTTTTTTCTCATTTTACTTTTTTATTATTATT   60
I_c13_12_dm28c     TGAGGGCACTCTTTGTTTCTGATGCTGCCTTTTTTCTCATTTTACTTTTTTATTATTATT   60
I_c14_13_dm28c     TGAGGGCACTCTTTGTTTCTGATGCTGCCTTTTTTCTCATTTTACTTTTTTATTATTATT   60
I_c15_14_dm28c     TGAGGGCACTCTTTGTTTCTGATGCTGCCTTTTTTCTCATTTTACTTTTTTATTATTATT   60
I_c2_1_NEL         TGAGGGCACTCTTTGTTTCTGATGCTGCCTTTTTCTCTATTTTACTTTTTTATTATTATT   60
I_c13_12_NEL       TGAGGGCACTCTTTGTTTCTGATGCTGCCTTTTTTCTCA-TTTACTTTTTTATTATTATT   59
I_c6_5_EL          TGAGGGCACTCTTTGTTTCTGATGCTGCCTTTTTTCTCATTTTACTTTTTTATTATTATT   60
I_c15_14_NEL       TGAGGGCACTCTTTGTTTCTGATGCTGCCTTTTTTCTCATTTTACTTTTTTATTATTATT   60
I_c12_11_NEL       TGAGGGCACTCTTTGTTTCTGATGCTGCCTTTTTTCTCATTTTACTTTTTTATTATTATT   60
I_c11_10_NEL       TGAGGGCACTCTTTGTTTCTGATGCTGCCTTTTTTCTCATTTTACTTTTTTATTATTATT   60
I_c9_8_NEL         TGAGGGCACTCTTTGTTTCTGATGCTGCCTTTTTTCTCATTTTACTTTTTTATTATTATT   60
I_c8_7_NEL         TGAGGGCACTCTTTGTTTCTGATGCTGCCTTTTTTCTCATTTTACTTTTTTATTATTATT   60
I_c3_2_NEL         TGAGGGCACTCTTTGTTTCTGATGCTGCCTTTTTTCTCATTTTACTTTTTTATTATTATT   60
I_c17_16_NEL       TGAGGGCACTCTTTGTTTCTGATGCTGCCTTTTTTCTCATTTTACTTTTTTATTATTATT   60
I_c18_17_NEL       TGAGGGCACTCTTTGTTTCTGATGCTGCCTTTTTTCTCATTTTACTTTTTTATTATTATT   60
I_c16_15_NEL       TGAGGGCACTCTTTGTTTCTGATGCTGCCTTTTTTCTCATTTTACTTTTTTATTATTATT   60
I_c10_9_NEL        TGAGGGCACTCTTTGTTTCTGATGCTGCCTTTTTTCTCATTTTACTTTTTTATTATTATT   60
I_c4_3_NEL         TGAGGGCACTCTTTGTTTCTGATGCTGCCTTTTTTCTCATTTTACTTTTTTATTATTATT   60
II_c8_7_NEL        TGAGGGCACTCTTTGTTTCTGATGCTGC-TTTTTTCTCATTTTCCTTCTTATTATTATT   59
II_c6_5_NEL        TGAGGGCACTCTTTGTTTCTGATGCTGC-TTTTTTCTCATTTTCCTTCTTATTATTATT   59
II_c10_9_NEL       TGAGGGCACTCTTTGTTTCTGATGCTGC-TTTTTTCTCATTTTACTTTCTTATTATTATT   59
II_c9_8_NEL        TGAGGGCACTCTTTGTTTCTGATGCTGC-TTTTTTCTCATTTTACTTTCTTATTATTATT   59
```

Figure S2 - **Alignment of intergenic sequences among tandem cruzipain copies from Dm28c, CL Brener and YC6 *T. cruzi* strains.** Figure continues on the next page.

```
II_c4_3_NEL        TGAGGGCACTCTTTGTTTCTGATGCTGC-TTTTTTCTCATTTTACTTTCTTATTATTATT   59
II_c11_10_NEL      TGAGGGCACTCTTTGTTTCTGATGCTGC-TTTTTTCTCATTTTACTTTCTTATTATTATT   59
II_c7_6_NEL        TGAGGGCACTCTTTGTTTCTGATGCTGC-TTTTTTCTCATTTTACTTTCTTATTATTATT   59
II_c5_4_NEL        TGAGGGCACTCTTTGTTTCTGATGCTGC-TTTTTTCTCATTTTACTTTCTTATTATTATT   59
II_c5_4_EL         TGAGGGCACTCTTTGTTTCTGATGCTGCCTTTTTTCTCATTTTACTTTCTTATTATTATT   60
II_c15_14_YC6      TGAGGGCACTCTTTGTTTCTGATGCTGCCTTTTTTCTCATTTTACTTTCTTATTATTATT   60
II_c13_12_YC6      TGAGGACACTCTTTGTTTCTGATGCTGCCTTTTTTCTCATTTTACTTTCTTATTATTATT   60
II_c12_11_YC6      TGAGGACACTCTTTGTTTCTGATGCTGCCTTTTTTCTCATTTTACTTTCTTATTATTATT   60
II_c11_10_YC6      TGAGGGCACTCTTTGTTTCTGATGCTGCCTTTTTTCTCATTTTACTTTCTTATTATTATT   60
II_c10_9_YC6       TGAGGGCACTCTTTGTTTCTGATGCTGCCTTTTTTCTCATTTTACTTTCTTATTATTATT   60
II_c9_8_YC6        TGAGGACACTCTTTGTTTCTGATGCTGCCTTTTTTCTCATTTTACTTTCTTATTATTATT   60
II_c3_2_EL         TGAGGACACTCTTTGTTTCTGATGCTGCCTTTTTTCTCATTTTACTTTCTTATTATTATT   60
II_c14_13_YC6      TGAGGACACTCTTTGTTTCTGATGCTGCCTTTTTTCTCATTTTACTTTCTTATTATTATT   60
II_c4_3_EL         TGAGGACACTCTTTGTTTCTGATGCTGCCTTTTTTCTCATTTTACTTTCTTATTATTATT   60
II_c2_1_dm28c      TGAGGGCACTCTTTGTTTCTGATGCTGCCTTTTTTCTCATTTTACTTTTTTATTATTATT   60
II_c4_3_dm28c      TGAGGGCACTCTTTGTTTCTGATGCTGCCTTTTTTCTCATTTTACTTTTTTATTATTATT   60
II_c5_4_dm28c      TGAGGGCACTCTTTGTTTCTGATGCTGCCTTTTTTCTCATTTTACTTTTTTATTATTATT   60
II_c6_5_dm28c      TGAGGGCACTCTTTGTTTCTGATGCTGCCTTTTTTCTCATTTTACTTTTTTATTATTATT   60
II_c8_7_dm28c      TGAGGGCACTCTTTGTTTCTGATGCTGCCTTTTTTCTCATTTTACTTTTTTATTATTATT   60
II_c3_2_dm28c      TGAGGGCACTCTTTGTTTCTGATGCTGCCTTTTTTCTCATTTTACTTTTTTATTATTATT   60
II_c9_8_dm28c      TGAGGGCACTCTTTGTTTCTGATGCTGCCTTTTTTCTCATTTTACTTTTTTATTATTATT   60
II_c7_6_dm28c      TGAGGGCACTCTTTGTTTCTGATGCTGCCTTTTTTCTCATTTTACTTTTTTATTATTATT   60
                   ***** *** ******************  *** ***** *** *  * ***********

I_c8_7_EL          GAGCGAGCGTCGGTGCTGCGGCACACTGGGACAAATAAGTCCCAGTGGAGTGGATTGTTT   120
I_c16_15_YC6       GAGCGAGCGTCGGTGCTGCGGCACACTGG-GCAAATAAGTCCCAGTGGAGTGGATTGTTT   118
I_c5_4_EL          GAGCGAGCGTCGGTGCTGCGGCACACTGG-GCAAATAAGTCCCAGTGGAGTGGATTGTTT   119
I_c18_17_YC6       GAGCGAGCGTCGGTGCTGCGGCACACTGG-ACAAATAAGTCCCAGTGGAGTGGATTGTTT   119
I_c17_16_YC6       GAGCGAGCGTCGGTGCTGCGGCACACTGG-GCAAATAAGTCCCAGTGGAGTGGATTGTTT   119
I_c10_9_EL         GAGCGAGCGTCGGTGCTGCGGCACACTGG-GCAAATAAGTCCCAGTGGAGTGGATTGTTT   119
I_c4_3_EL          GAGCGAGCGTCGGTGCTGCGGCACACTGG-GCAAATAAGTCCCAGTGGAGTGGATTGTTT   119
I_c3_2_EL          GAGCGAGCGTCGGTGCTGCGGCACACTGG-GCAAATAAGTCCCAGTGGAGTGGATTGTTT   119
I_c2_1_EL          GAGCGAGCGTCGGTGCTGCGGCACACTGG-GCAAATAAGTCCCAGTGGAGTGGATTGTTT   119
I_c2-1_YC6         GAGCGAGCGTCGGTGCTGCGGCACACTGG-GCAAATAAGTCCCAGTGGAGTGGATTGTTT   119
I_c3_2_YC6         GAGCGAGCGTCGGTGCTGCGGCACACTGG-GCAAATAAGTCCCAGTGGAGTGGATTGTTT   119
I_c4_3_YC6         GAGCGAGCGTCGGTGCTGCGGCACACTGG-GCAAATAAGTCCCAGTGGAGTGGATTGTTT   119
I_c5_4_YC6         GAGCGAGCGTCGGTGCTGCGGCACACTGG-GCAAATAAGTCCCAGTGGAGTGGATTGTTT   119
I_c6_5_YC6         GAGCGAGCGTCGGTGCTGCGGCACACTGG-GCAAATAAGTCCCAGTGGAGTGGATTGTTT   119
I_Yc7_6_YC6        GAGCGAGCGTCGGTGCTGCGGCACACTGG-GCAAATAAGTCCCAGTGGAGTGGATTGTTT   119
I_c8_7_YC6         GAGCGAGCGTCGGTGCTGCGGCACACTGG-GCAAATAAGTCCCAGTGGAGTGGATTGTTT   119
I_c9_8_YC6         GAGCGAGCGTCGGTGCTGCGGCACACTGG-GCAAATAAGTCCCAGTGGAGTGGATTGTTT   119
I_c10_9_YC6        GAGCGAGCGTCGGTGCTGCGGCACACTGG-GCAAATAAGTCCCAGTGGAGTGGATTGTTT   119
I_c11_10_YC6       GAGCGAGCGTCGGTGCTGCGGCACACTGG-GCAAATAAGTCCCAGTGGAGTGGATTGTTT   119
I_c12_11_YC6       GAGCGAGCGTCGGTGCTGCGGCACACTGG-GCAAATAAGTCCCAGTGGAGTGGATTGTTT   119
I_c13_12_YC6       GAGCGAGCGTCGGTGCTGCGGCACACTGG-GCAAATAAGTCCCAGTGGAGTGGATTGTTT   119
I_c14_13_YC6       GAGCGAGCGTCGGTGCTGCGGCACACTGG-GCAAATAAGTCCCAGTGGAGTGGATTGTTT   119
I_c15_14_YC6       GAGCGAGCGTCGGTGCTGCGGCACACTGG-GCAAATAAGTCCCAGTGGAGTGGATTGTTT   119
III_c2_1_YC6       GAGCGAGCGTCGGTGCTGCGGCACACTGG-GCAAATAAGTCCCAGTGGAGTGGATTGTTT   119
III_c3_2_YC6       GAGCGAGCGTCGGTGCTGCGGCACACTGG-GCAAATAAGTCCCAGTGGAGTGGATTGTTT   119
III_c4_3_YC6       GAGCGAGCGTCGGTGCTGCGGCACACTGG-GCAAATAAGTCCCAGTGGAGTGGATTGTTT   119
III_c5_4_YC6       GAGCGAGCGTCGGTGCTGCGGCACACTGG-GCAAATAAGTCCCAGTGGAGTGGATTGTTT   119
III_c6_5_YC6       GAGCGAGCGTCGGTGCTGCGGCACACTGG-GCAAATAAGTCCCAGTGGAGTGGATTGTTT   119
III_c7_6_YC6       GAGCGAGCGTCGGTGCTGCGGCACACTGG-GCAAATAAGTCCCAGTGGAGTGGATTGTTT   119
I_c9_8_EL          GAGCGAGCGTCGGTGCTGCGGCACACTGG-ACAAATAAGTCCCAGTGGAGTGGATTGTTT   119
I_c14_13_NEL       GAGCGAGCGTCGGTGCTGCGGCACACTGG-GCAAATAAGTCCCAGTGGAGTGGATTGTTT   118
I_c5_4_NEL         GAGCGAGCGTCGGTGCTGCGGCACACTGG-GCAAATAAGTCCCAGTGGAGTGGATTGTTT   119
I_c7_6_NEL         GAGCGAGCGTCGGTGCTGCGGCACACTGG-GCAAATAAGTCCCAGTGGAGTGGATTGTTT   119
I_c2_1_dm28c       GAGCGAGAGTCGGTGCTGCGGCACACTGG-GCAAATAAGTCCCAGTGGAGTGGATTGCTT   119
I_c3_2_dm28c       GAGCGAGAGTCGGTGCTGCGGCACACTGG-GCAAATAAGTCCCAGTGGAGTGGATTGCTT   119
I_c4_3_dm28c       GAGCGAGAGTCGGTGCTGCGGCACACTGG-GCAAATAAGTCCCAGTGGAGTGGATTGCTT   119
I_c5_4_dm28c       GAGCGAGAGTCGGTGCTGCGGCACACTGG-GCAAATAAGTCCCAGTGGAGTGGATTGCTT   119
I_c6_5_dm28c       GAGCGAGAGTCGGTGCTGCGGCACACTGG-GCAAATAAGTCCCAGTGGAGTGGATTGCTT   119
I_c7_6_dm28c       GAGCGAGAGTCGGTGCTGCGGCACACTGG-GCAAATAAGTCCCAGTGGAGTGGATTGCTT   119
I_c8_7_dm28c       GAGCGAGAGTCGGTGCTGCGGCACACTGG-GCAAATAAGTCCCAGTGGAGTGGATTGCTT   119
I_c9_8_dm28c       GAGCGAGAGTCGGTGCTGCGGCACACTGG-GCAAATAAGTCCCAGTGGAGTGGATTGCTT   119
I_c10_9_dm28c      GAGCGAGAGTCGGTGCTGCGGCACACTGG-GCAAATAAGTCCCAGTGGAGTGGATTGCTT   119
I_c11_10_dm28c     GAGCGAGAGTCGGTGCTGCGGCACACTGG-GCAAATAAGTCCCAGTGGAGTGGATTGCTT   119
```

Figure S2 - **Alignment of intergenic sequences among tandem cruzipain copies from Dm28c, CL Brener and YC6 *T. cruzi* strains.** Figure continues on the next page.

```
I_c12_11_dm28c   GAGCGAGAGTCGGTGCTGCGGCACACTGG-GCAAATAAGTCCCAGTGGAGTGGATTGCTT   119
I_c13_12_dm28c   GAGCGAGAGTCGGTGCTGCGGCACACTGG-GCAAATAAGTCCCAGTGGAGTGGATTGCTT   119
I_c14_13_dm28c   GAGCGAGAGTCGGTGCTGCGGCACACTGG-GCAAATAAGTCCCAGTGGAGTGGATTGCTT   119
I_c15_14_dm28c   GAGCGAGAGTCGGTGCTGCGGCACACTGG-GCAAATAAGTCCCAGTGGAGTGGATTGCTT   119
I_c2_1_NEL       GAGCGAGCGTCGGTGCTGCGGCACACTGG-GCAAATAAGTCCCAGTGGAGTGGATTGTTT   119
I_c13_12_NEL     GAGCGAGCGTCGGTGCTGCGGCACACTGG-GCAAATAAGTCCCAGTGGAGTGGATTGTTT   118
I_c6_5_EL        GAGCGAGCGTCGGTGCTGCGGCACACTGG-GCAAATAAGTCCCAGTGGAGTGGATTGTTT   119
I_c15_14_NEL     GAGCGAGCGTCGGTGCTGCGGCACACTGG-GCAAATAAGTCCCAGTGGAGTGGATTGTTT   119
I_c12_11_NEL     GAGCGAGCGTCGGTGCTGCGGCACACTGG-GCAAATAAGTCCCAGTGGAGTGGATTGTTT   119
I_c11_10_NEL     GAGCGAGCGTCGGTGCTGCGGCACACTGG-GCAAATAAGTCCCAGTGGAGTGGATTGTTT   119
I_c9_8_NEL       GAGCGAGCGTCGGTGCTGCGGCACACTGG-GCAAATAAGTCCCAGTGGAGTGGATTGTTT   119
I_c8_7_NEL       GAGCGAGCGTCGGTGCTGCGGCACACTGG-GCAAATAAGTCCCAGTGGAGTGGATTGTTT   119
I_c3_2_NEL       GAGCGAGCGTCGGTGCTGCGGCACACTGG-GCAAATAAGTCCCAGTGGAGTGGATTGTTT   119
I_c17_16_NEL     GAGCGAGCGTCGGTGCTGCGGCACACTGG-GCAAATAAGTCCCAGTGGAGTGGATTGTTT   119
I_c18_17_NEL     GAGCGAGCGTCGGTGCTGCGGCACACTGG-GCAAATAAGTCCCAGTGGAGTGGATTGTTT   119
I_c16_15_NEL     GAGCGAGCGTCGGTGCTGCGGCACACTGG-GCAAATAAGTCCCAGTGGAGTGGATTGTTT   119
I_c10_9_NEL      GAGCGAGCGTCGGTGCTGCGGCACACTGG-GCAAATAAGTCCCAGTGGAGTGGATTGTTT   119
I_c4_3_NEL       GAGCGAGCGTCGGTGCTGCGGCACACTGG-GCAAATAAGTCCCAGTGGAGTGGATTGTTT   119
II_c8_7_NEL      GAGCGAGCGTCGGTGCTGCGGCACACCGGGAAAA-CAAGTCCCAGTGGAGTGGATCGTTT   118
II_c6_5_NEL      GAGCGAGCGTCGGTGCTGCGGCACACCGGGAAAA-CAAGTCCCAGTGGAGTGGATCGTTT   118
II_c10_9_NEL     GAGCGAGCGTCGGTGCTGCGGCACACCGGGAAAA-CAAGTCCCAGTGGAGTGGATCGTTT   118
II_c9_8_NEL      GAGCGAGCGTCGGTGCTGCGGCACACCGGGAAAA-CAAGTCCCAGTGGAGTGGATCGTTT   118
II_c4_3_NEL      GAGCGAGCGTCGGTGCTGCGGCACACCGGGAAAA-CAAGTCCCAGTGGAGTGGATCGTTT   118
II_c11_10_NEL    GAGCGAGCGTCGGTGCTGCGGCACACCGGGAAAA-CAAGTCCCAGTGGAGTGGATCGTTT   118
II_c7_6_NEL      GAGCGAGCGTCGGTGCTGCGGCACACCGGGAAAA-CAAGTCCCAGTGGAGTGGATCGTTT   118
II_c5_4_NEL      GAGCGAGCGTCGGTGCTGCGGCACACCGGGAAAA-CAAGTCCCAGTGGAGTGGATCGTTT   118
II_c5_4_EL       GAGCAAGCGTTGGTGCTGCGGCACACTGGGCAAA-TAAGTCCCAATGGGGTGGATCGTTT   119
II_c15_14_YC6    GAGCAAGCGTTGGTGCTGCGGCACACTGGGCAAA-TAAGTCCCAATGGGGTGGATCGTTT   119
II_c13_12_YC6    GAGCAAGCGTTGGTGCTGCAGCACACTGGGCAAA-TAAGTCCCAATGGGGTGGATCGTTT   119
II_c12_11_YC6    GAGCAAGCGTTGGTGCTGCGGCACACTGGGCAAA-TAAGTCCCAATGGGGTGGATCGTTT   119
II_c11_10_YC6    GAGCAAGCGTTGGTGCTGCGGCACACTGGGCAAA-TAAGTCCCAATGGGGTGGATCGTTT   119
II_c10_9_YC6     GAGCAAGCGTTGGTGCTGCGGCACACTGGGCAAA-TAAGTCCCAATGGGGTGGATCGTTT   119
II_c9_8_YC6      GAGCAAGCGTTGGTGCTGCGGTACACTGGGCAAA-TAAGTCCCAATGGGGTGGATCGTTT   119
II_c3_2_EL       GAGCAAGCGTTGGTGCTGCGGCACACTGGGCAAA-TAAGTCCCAATGGGGTGGATCGTTT   119
II_c14_13_YC6    GAGCAAGCGTTGGTGCTGCGGCACACTGGGCAAA-TAAGTCCCAATGGGGTGGATCGTTT   119
II_c4_3_EL       GAGCAAGCGTTGGTGCTGCGGCACACTGGGCAAA-TAAGTCCCAATGGGGTGGATCGTTT   119
II_c2_1_dm28c    GAGCAAGCGTTGGTTCTCATTATTATTGGGCAAA-TAATTCCCAATGGGGTGGATCGTTT   119
II_c4_3_dm28c    GAGCAAGCGTTGGTTCTCATTATTATTGGGCAAA-TAATTCCCAATGGGGTGGATCGTTT   119
II_c5_4_dm28c    GAGCAAGCGTTGGTTCTCATTATTATTGGGCAAA-TAATTCCCAATGGGGTGGATCGTTT   119
II_c6_5_dm28c    GAGCAAGCGTTGGTTCTCATTATTATTGGGCAAA-TAATTCCCAATGGGGTGGATCGTTT   119
II_c8_7_dm28c    GAGCAAGCGTTGGTTCTCATTATTATTGGGCAAA-TAATTCCCAATGGGGTGGATCGTTT   119
II_c3_2_dm28c    GAGCAAGCGTTGGTTCTCATTATTATTGGGCAAA-TAATTCCCAATGGAGTGGATCGTTT   119
II_c9_8_dm28c    GAGCAAGCGTTGGTTCTCATTATTATTGGGCAAA-TAATTCCCAATGGAGTGGATCGTTT   119
II_c7_6_dm28c    GAGCAAGCGTTGGTTCTCATTATTATTGGGCAAA-TAATTCCCAATGGAGTGGATCGTTT   119
                 **** ** ** *** **       *  **   **  ** ***** *** ****** * **

I_c8_7_EL        ATTGATTTGCTCCATGTATGTGTTTTCTTTTTTGTGCACAACTGCAAATATGTGTCCGCG   180
I_c16_15_YC6     ATTGATTTGCTCCATGTATGTGTTTTCTTTTTTGTGCACAACTGCAAATATGTGTCCGCG   178
I_c5_4_EL        ATTGATTTGCTCCATGTATGTGTTTTCTTTTTTGTGCACAACTGCAAATATGTGTCCGCG   179
I_c18_17_YC6     ATTGATTTGCTCCATGTATGTGTTTTCTTTTTTGTGCACAACTGCAAATATGTGTCCGCG   179
I_c17_16_YC6     ATTGATTTGCTCCATGTATGTGTTTTCTTTTTTGTGCACAACTGCAAATATGTGTCCGCG   179
I_c10_9_EL       ATTGATTTGCTCCATGTATGTGTTTTCTTTTTTGTGCACAACTGCAAATATGTGTCCGCG   179
I_c4_3_EL        ATTGATTTGCTCCATGTATGTGTTTTCTTTTTTGTGCACAACTGCAAATATGTGTCCGCG   179
I_c3_2_EL        ATTGATTTGCTCCATGTATGTGTTTTCTTTTTTGTGCACAACTGCAAATATGTGTCCGCG   179
I_c2_1_EL        ATTGATTTGCTCCATGTATGTGTTTTCTTTTTTGTGCACAACTGCAAATATGTGTCCGCG   179
I_c2-1_YC6       ATTGATTTGCTCCATGTATGTGTTTTCTTTTTTGTGCACAACTGCAAATATGTGTCCGCG   179
I_c3_2_YC6       ATTGATTTGCTCCATGTATGTGTTTTCTTTTTTGTGCACAACTGCAAATATGTGTCCGCG   179
I_c4_3_YC6       ATTGATTTGCTCCATGTATGTGTTTTCTTTTTTGTGCACAACTGCAAATATGTGTCCGCG   179
I_c5_4_YC6       ATTGATTTGCTCCATGTATGTGTTTTCTTTTTTGTGCACAACTGCAAATATGTGTCCGCG   179
I_c6_5_YC6       ATTGATTTGCTCCATGTATGTGTTTTCTTTTTTGTGCACAACTGCAAATATGTGTCCGCG   179
I_Yc7_6_YC6      ATTGATTTGCTCCATGTATGTGTTTTCTTTTTTGTGCACAACTGCAAATATGTGTCCGCG   179
I_c8_7_YC6       ATTGATTTGCTCCATGTATGTGTTTTCTTTTTTGTGCACAACTGCAAATATGTGTCCGCG   179
I_c9_8_YC6       ATTGATTTGCTCCATGTATGTGTTTTCTTTTTTGTGCACAACTGCAAATATGTGTCCGCG   179
I_c10_9_YC6      ATTGATTTGCTCCATGTATGTGTTTTCTTTTTTGTGCACAACTGCAAATATGTGTCCGCG   179
I_c11_10_YC6     ATTGATTTGCTCCATGTATGTGTTTTCTTTTTTGTGCACAACTGCAAATATGTGTCCGCG   179
I_c12_11_YC6     ATTGATTTGCTCCATGTATGTGTTTTCTTTTTTGTGCACAACTGCAAATATGTGTCCGCG   179
I_c13_12_YC6     ATTGATTTGCTCCATGTATGTGTTTTCTTTTTTGTGCACAACTGCAAATATGTGTCCGCG   179
```

Figure S2 - **Alignment of intergenic sequences among tandem cruzipain copies from Dm28c, CL Brener and YC6 *T. cruzi* strains.** Figure continues on the next page.

```
I_c14_13_YC6         ATTGATTTGCTCCATGTATGTGTTTTCTTTTTTGTGCACAACTGCAAATATGTGTCCGCG   179
I_c15_14_YC6         ATTGATTTGCTCCATGTATGTGTTTTCTTTTTTGTGCACAACTGCAAATATGTGTCCGCG   179
III_c2_1_YC6         ATTGATTTGCTCCATGTATGTGTTTTCTTTTTTGTGCACAACTGCAAATATGTGTCCGCG   179
III_c3_2_YC6         ATTGATTTGCTCCATGTATGTGTTTTCTTTTTTGTGCACAACTGCAAATATGTGTCCGCG   179
III_c4_3_YC6         ATTGATTTGCTCCATGTATGTGTTTTCTTTTTTGTGCACAACTGCAAATATGTGTCCGCG   179
III_c5_4_YC6         ATTGATTTGCTCCATGTATGTGTTTTCTTTTTTGTGCACAACTGCAAATATGTGTCCGCG   179
III_c6_5_YC6         ATTGATTTGCTCCATGTATGTGTTTTCTTTTTTGTGCACAACTGCAAATATGTGTCCGCG   179
III_c7_6_YC6         ATTGATTTGCTCCATGTATGTGTTTTCTTTTTTGTGCACAACTGCAAATATGTGTCCGCG   179
I_c9_8_EL            ATTGATTTGCTCCATGTATGTGTTTTCTTTTTTGTGCACAACTGCAAATATGTGTCCGCG   179
I_c14_13_NEL         ATTGATTTGCTCCATGTATTTATT-TCTTTTTTGAGCACAACTGCAAATATGTGTCCGCG   177
I_c5_4_NEL           ATTGATTTGCTCCATGTATTTATTTTCTTTTTTGAGCACAACTGCAAATATGTGTCCGCG   179
I_c7_6_NEL           ATTGATTTGCTCCATGTATTTATTTTCTTTTTTGAGCACAACTGCAAATATGTGTCCGCG   179
I_c2_1_dm28c         ATTGATTTGCTCCATGTAATTATTTTCTTTTTTGAGCACAACTGCAAATATGTGTCCGCG   179
I_c3_2_dm28c         ATTGATTTGCTCCATGTAATTATTTTCTTTTTTGAGCACAACTGCAAATATGTGTCCGCG   179
I_c4_3_dm28c         ATTGATTTGCTCCATGTAATTATTTTCTTTTTTGAGCACAACTGCAAATATGTGTCCGCG   179
I_c5_4_dm28c         ATTGATTTGCTCCATGTAATTATTTTCTTTTTTGAGCACAACTGCAAATATGTGTCCGCG   179
I_c6_5_dm28c         ATTGATTTGCTCCATGTAATTATTTTCTTTTTTGAGCACAACTGCAAATATGTGTCCGCG   179
I_c7_6_dm28c         ATTGATTTGCTCCATGTAATTATTTTCTTTTTTGAGCACAACTGCAAATATGTGTCCGCG   179
I_c8_7_dm28c         ATTGATTTGCTCCATGTAATTATTTTCTTTTTTGAGCACAACTGCAAATATGTGTCCGCG   179
I_c9_8_dm28c         ATTGATTTGCTCCATGTAATTATTTTCTTTTTTGAGCACAACTGCAAATATGTGTCCGCG   179
I_c10_9_dm28c        ATTGATTTGCTCCATGTAATTATTTTCTTTTTTGAGCACAACTGCAAATATGTGTCCGCG   179
I_c11_10_dm28c       ATTGATTTGCTCCATGTAATTATTTTCTTTTTTGAGCACAACTGCAAATATGTGTCCGCG   179
I_c12_11_dm28c       ATTGATTTGCTCCATGTAATTATTTTCTTTTTTGAGCACAACTGCAAATATGTGTCCGCG   179
I_c13_12_dm28c       ATTGATTTGCTCCATGTAATTATTTTCTTTTTTGAGCACAACTGCAAATATGTGTCCGCG   179
I_c14_13_dm28c       ATTGATTTGCTCCATGTAATTATTTTCTTTTTTGAGCACAACTGCAAATATGTGTCCGCG   179
I_c15_14_dm28c       ATTGATTTGCTCCATGTAATTATTTTCTTTTTTGAGCACAACTGCAAATATGTGTCCGCG   179
I_c2_1_NEL           ATTGATTTGCTCCATGTATTTATTTTCTT-TTTGAGCACAACTGCAAATATGTGTCCGCG   178
I_c13_12_NEL         ATTGATTTGCTCCATGTATTTATTTTCTTTTTTGAGCACAACTGCAAATATGTGTCCGCG   178
I_c6_5_EL            ATTGATTTGCTCCATGTATTTATTTTCTTTTTTGAGCACAACTGCAAATATGTGTCCGCG   179
I_c15_14_NEL         ATTGATTTGCTCCATGTATTTATTTTCTTTTTTGAGCACAACTGCAAATATGTGTCCGCG   179
I_c12_11_NEL         ATTGATTTGCTCCATGTATTTATTTTCTTTTTTGAGCACAACTGCAAATATGTGTCCGCG   179
I_c11_10_NEL         ATTGATTTGCTCCATGTATTTATTTTCTTTTTTGAGCACAACTGCAAATATGTGTCCGCG   179
I_c9_8_NEL           ATTGATTTGCTCCATGTATTTATTTTCTTTTTTGAGCACAACTGCAAATATGTGTCCGCG   179
I_c8_7_NEL           ATTGATTTGCTCCATGTATTTATTTTCTTTTTTGAGCACAACTGCAAATATGTGTCCGCG   179
I_c3_2_NEL           ATTGATTTGCTCCATGTATTTATTTTCTTTTTTGAGCACAACTGCAAATATGTGTCCGCG   179
I_c17_16_NEL         ATTGATTTGCTCCATGTATTTATTTTCTTTTTTGAGCACAACTGCAAATATGTGTCCGCG   179
I_c18_17_NEL         ATTGATTTGCTCCATGTATTTATTTTCTTTTTTGAGCACAACTGCAAATATGTGTCCGCG   179
I_c16_15_NEL         ATTGATTTGCTCCATGTATTTATTTTCTTTTTTGAGCACAACTGCAAATATGTGTCCGCG   179
I_c10_9_NEL          ATTGATTTGCTCCATGTATTTATTTTCTTTTTTGAGCACAACTGCAAATATGTGTCCGCG   179
I_c4_3_NEL           ATTGATTTGCTCCATGTATTTATTTTCTTTTTTGAGCACAACTGCAAATATGTGTCCGCG   179
II_c8_7_NEL          ATTGATTTGCTCT-----------------------------------------------   131
II_c6_5_NEL          ATTGATTTGCTCT-----------------------------------------------   131
II_c10_9_NEL         ATTGATTTGCTCT-----------------------------------------------   131
II_c9_8_NEL          ATTGATTTGCTCT-----------------------------------------------   131
II_c4_3_NEL          ATTGATTTGCTCT-----------------------------------------------   131
II_c11_10_NEL        ATTGATTTGCTCT-----------------------------------------------   131
II_c7_6_NEL          ATTGATTTGCTCT-----------------------------------------------   131
II_c5_4_NEL          ATTGATTTGCTCT-----------------------------------------------   131
II_c5_4_EL           ATTGATTTTCTCT-----------------------------------------------   132
II_c15_14_YC6        ATTGATTTTCTCT-----------------------------------------------   132
II_c13_12_YC6        ATTGATTTTCTCT-----------------------------------------------   132
II_c12_11_YC6        ATTGATTTTCTCT-----------------------------------------------   132
II_c11_10_YC6        ATTGATTTTCTCT-----------------------------------------------   132
II_c10_9_YC6         ATTGATTTTCTCT-----------------------------------------------   132
II_c9_8_YC6          ATTGATTTTCTCT-----------------------------------------------   132
II_c3_2_EL           ATTGATTTTCTCT-----------------------------------------------   132
II_c14_13_YC6        ATTGATTTTCTCT-----------------------------------------------   132
II_c4_3_EL           ATTGATTTTCTCT-----------------------------------------------   132
II_c2_1_dm28c        ATTGATTTGCTCT-----------------------------------------------   132
II_c4_3_dm28c        ATTGATTTGCTCT-----------------------------------------------   132
II_c5_4_dm28c        ATTGATTTGCTCT-----------------------------------------------   132
II_c6_5_dm28c        ATTGATTTGCTCT-----------------------------------------------   132
II_c8_7_dm28c        ATTGATTTGCTCT-----------------------------------------------   132
II_c3_2_dm28c        ATTGATTTGCTCT-----------------------------------------------   132
II_c9_8_dm28c        ATTGATTTGCTCT-----------------------------------------------   132
II_c7_6_dm28c        ATTGATTTGCTCT-----------------------------------------------   132
                     ******** ***
```

Figure S2 - **Alignment of intergenic sequences among tandem cruzipain copies from Dm28c, CL Brener and YC6 *T. cruzi* strains.** Figure continues on the next page.

```
I_c8_7_EL          ATCGACGTCTCAGCCACGCGTGTGTTTTTTTCTATTATATGCTTCTGTTGAGCGCCCGAGC  240
I_c16_15_YC6       ATCGACGTCTCAGCCACGCGTGTGTTTTTTTCTATTGTATGCTTCTGTTGAGCGCCCGAGC  238
I_c5_4_EL          ATCGACGTCTCAGCCACGCGTGTGTTTTTTTCTATTATATGCTTCTGTTGAGCGCCCGAGC  239
I_c18_17_YC6       ATCGACTTCTCAGCCACGCGTGTGTTTTTTTCTACTATATGCTTCTGTTGAGCGCCCGAGC  239
I_c17_16_YC6       ATCGACGTCTCAGCCACGCGTGTGTTTTTTTCTATTGTATGCTTCTGTTGAGCGCCCGAGC  239
I_c10_9_EL         ATCGACGTCTCAGCCACGCGTGTGTTTTTTTCTATTATATGCTTCTGTTGAGCGCCCGAGC  239
I_c4_3_EL          ATCGACGTCTCAGCCACGCGTGTGTTTTTTTCTATTATATGCTTCTGTTGAGCGCCCGAGC  239
I_c3_2_EL          ATCGACGTCTCAGCCACGCGTGTGTTTTTTTCTATTATATGCTTCTGTTGAGCGCCCGAGC  239
I_c2_1_EL          ATCGACGTCTCAGCCACGCGTGTGTTTTTTTCTATTATATGCTTCTGTTGAGCGCCCGAGC  239
I_c2-1_YC6         ATCGACGTCTCAGCCACGCGTGTGTTTTTTTCTATTATATGCTTCTGTTGAGCGCCCGAGC  239
I_c3_2_YC6         ATCGACGTCTCAGCCACGCGTGTGTTTTTTTCTATTATATGCTTCTGTTGAGCGCCCGAGC  239
I_c4_3_YC6         ATCGACGTCTCAGCCACGCGTGTGTTTTTTTCTATTATATGCTTCTGTTGAGCGCCCGAGC  239
I_c5_4_YC6         ATCGACGTCTCAGCCACGCGTGTGTTTTTTTCTATTATATGCTTCTGTTGAGCGCCCGAGC  239
I_c6_5_YC6         ATCGACGTCTCAGCCACGCGTGTGTTTTTTTCTATTATATGCTTCTGTTGAGCGCCCGAGC  239
I_Yc7_6_YC6        ATCGACGTCTCAGCCACGCGTGTGTTTTTTTCTATTATATGCTTCTGTTGAGCGCCCGAGC  239
I_c8_7_YC6         ATCGACGTCTCAGCCACGCGTGTGTTTTTTTCTATTATATGCTTCTGTTGAGCGCCCGAGC  239
I_c9_8_YC6         ATCGACGTCTCAGCCACGCGTGTGTTTTTTTCTATTATATGCTTCTGTTGAGCGCCCGAGC  239
I_c10_9_YC6        ATCGACGTCTCAGCCACGCGTGTGTTTTTTTCTATTATATGCTTCTGTTGAGCGCCCGAGC  239
I_c11_10_YC6       ATCGACGTCTCAGCCACGCGTGTGTTTTTTTCTATTATATGCTTCTGTTGAGCGCCCGAGC  239
I_c12_11_YC6       ATCGACGTCTCAGCCACGCGTGTGTTTTTTTCTATTATATGCTTCTGTTGAGCGCCCGAGC  239
I_c13_12_YC6       ATCGACGTCTCAGCCACGCGTGTGTTTTTTTCTATTATATGCTTCTGTTGAGCGCCCGAGC  239
I_c14_13_YC6       ATCGACGTCTCAGCCACGCGTGTGTTTTTTTCTATTATATGCTTCTGTTGAGCGCCCGAGC  239
I_c15_14_YC6       ATCGACGTCTCAGCCACGCGTGTGTTTTTTTCTATTATATGCTTCTGTTGAGCGCCCGAGC  239
III_c2_1_YC6       ATCGACGTCTCAGCCACGCGTGTGTTTTTTTCTATTATATGCTTCTGTTGAGCGCCCGAGC  239
III_c3_2_YC6       ATCGACGTCTCAGCCACGCGTGTGTTTTTTTCTATTATATGCTTCTGTTGAGCGCCCGAGC  239
III_c4_3_YC6       ATCGACGTCTCAGCCACGCGTGTGTTTTTTTCTATTATATGCTTCTGTTGAGCGCCCGAGC  239
III_c5_4_YC6       ATCGACGTCTCAGCCACGCGTGTGTTTTTTTCTATTATATGCTTCTGTTGAGCGCCCGAGC  239
III_c6_5_YC6       ATCGACGTCTCAGCCACGCGTGTGTTTTTTTCTATTATATGCTTCTGTTGAGCGCCCGAGC  239
III_c7_6_YC6       ATCGACGTCTCAGCCACGCGTGTGTTTTTTTCTATTATATGCTTCTGTTGAGCGCCCGAGC  239
I_c9_8_EL          ATCGACTTCTCAGCCACGCGTGTGTTTTTTTCTATTATATGCTTCTGTTGAGCGCCCGAGC  239
I_c14_13_NEL       ATCGACGTCTCAGCCACGCGTGTGTTTTTTTCTATTATATGCTTCTGTTGAGCTCCCGAGC  237
I_c5_4_NEL         ATCGACGTCTCAGCCACGCGTGTGTTT-TTCTATTATATGCTTCTGTTGAGCTCCCGAGC  238
I_c7_6_NEL         ATCGACGTCTCAGCCACGCGTGTGTTTTTTTCTATTATATGCTTCTGTTGAGCTCCCGAGC  239
I_c2_1_dm28c       ATCGACGTCTCAGCCACGCGTGTGTTTTTTTCTATTATATGCTTCTGTTGAGCGCCCGAGC  239
I_c3_2_dm28c       ATCGACGTCTCAGCCACGCGTGTGTTTTTTTCTATTATATGCTTCTGTTGAGCGCCCGAGC  239
I_c4_3_dm28c       ATCGACGTCTCAGCCACGCGTGTGTTTTTTTCTATTATATGCTTCTGTTGAGCGCCCGAGC  239
I_c5_4_dm28c       ATCGACGTCTCAGCCACGCGTGTGTTTTTTTCTATTATATGCTTCTGTTGAGCGCCCGAGC  239
I_c6_5_dm28c       ATCGACGTCTCAGCCACGCGTGTGTTTTTTTCTATTATATGCTTCTGTTGAGCGCCCGAGC  239
I_c7_6_dm28c       ATCGACGTCTCAGCCACGCGTGTGTTTTTTTCTATTATATGCTTCTGTTGAGCGCCCGAGC  239
I_c8_7_dm28c       ATCGACGTCTCAGCCACGCGTGTGTTTTTTTCTATTATATGCTTCTGTTGAGCGCCCGAGC  239
I_c9_8_dm28c       ATCGACGTCTCAGCCACGCGTGTGTTTTTTTCTATTATATGCTTCTGTTGAGCGCCCGAGC  239
I_c10_9_dm28c      ATCGACGTCTCAGCCACGCGTGTGTTTTTTTCTATTATATGCTTCTGTTGAGCGCCCGAGC  239
I_c11_10_dm28c     ATCGACGTCTCAGCCACGCGTGTGTTTTTTTCTATTATATGCTTCTGTTGAGCGCCCGAGC  239
I_c12_11_dm28c     ATCGACGTCTCAGCCACGCGTGTGTTTTTTTCTATTATATGCTTCTGTTGAGCGCCCGAGC  239
I_c13_12_dm28c     ATCGACGTCTCAGCCACGCGTGTGTTTTTTTCTATTATATGCTTCTGTTGAGCGCCCGAGC  239
I_c14_13_dm28c     ATCGACGTCTCAGCCACGCGTGTGTTTTTTTCTATTATATGCTTCTGTTGAGCGCCCGAGC  239
I_c15_14_dm28c     ATCGACGTCTCAGCCACGCGTGTGTTTTTTTCTATTATATGCTTCTGTTGAGCGCCCGAGC  239
I_c2_1_NEL         ATCGACGTCTCAGCCACGCGTGTGTTTTTTTCTATTATATGCTTCTGTTGAGCTCCCGAGC  238
I_c13_12_NEL       ATCGACGTCTCAGCCACGCGTGTGTTTTTTTCTATTATATGCTTCTGTTGAGCTCCCGAGC  238
I_c6_5_EL          ATCGACGTCTCAGCCACGCGTGTGTTTTTTTCTATTATATGCTTCTGTTGAGCTCCCGAGC  239
I_c15_14_NEL       ATCGACGTCTCAGCCACGCGTGTGTTTTTTTCTATTATATGCTTCTGTTGAGCTCCCGAGC  239
I_c12_11_NEL       ATCGACGTCTCAGCCACGCGTGTGTTTTTTTCTATTATATGCTTCTGTTGAGCTCCCGAGC  239
I_c11_10_NEL       ATCGACGTCTCAGCCACGCGTGTGTTTTTTTCTATTATATGCTTCTGTTGAGCTCCCGAGC  239
I_c9_8_NEL         ATCGACGTCTCAGCCACGCGTGTGTTTTTTTCTATTATATGCTTCTGTTGAGCTCCCGAGC  239
I_c8_7_NEL         ATCGACGTCTCAGCCACGCGTGTGTTTTTTTCTATTATATGCTTCTGTTGAGCTCCCGAGC  239
I_c3_2_NEL         ATCGACGTCTCAGCCACGCGTGTGTTTTTTTCTATTATATGCTTCTGTTGAGCTCCCGAGC  239
I_c17_16_NEL       ATCGACGTCTCAGCCACGCGTGTGTTTTTTTCTATTATATGCTTCTGTTGAGCGCCCGAGC  239
I_c18_17_NEL       ATCGACGTCTCAGCCACGCGTGTGTTTTTTTCTATTATATGCTTCTGTTGAGCTCCCGAGC  239
I_c16_15_NEL       ATCGACGTCTCAGCCACGCGTGTGTTTTTTTCTATTATATGCTTCTGTTGAGCTCCCGAGC  239
I_c10_9_NEL        ATCGACGTCTCAGCCACGCGTGTGTTTTTTTCTATTATATGCTTCTGTTGAGCTCCCGAGC  239
I_c4_3_NEL         ATCGACGTCTCAGCCACGCGTGTGTTTTTTTCTATTATATGCTTCTGTTGAGCTCCCGAGC  239
II_c8_7_NEL        ---------------GTGCATGCTTTTTTTCTATTATATGCTTCTGTTGAGCTCCCGAGC  176
II_c6_5_NEL        ---------------GTGCATGCTTTTTTTCTATTATATGCTTCTGTTGAGCTCCCGAGC  176
II_c10_9_NEL       ---------------GTGCATGCTTTTTTTCTATTATATGCTTCTGTTGAGCTCCCGAGC  176
II_c9_8_NEL        ---------------GTGCATGCTTTTTTTCTATTATATGCTTCTGTTGAGCTCCCGAGC  176
II_c4_3_NEL        ---------------GTGCATGCTTTTTTTCTATTATATGCTTCTGTTGAGCGCCCGAGC  176
```

Figure S2 - **Alignment of intergenic sequences among tandem cruzipain copies from Dm28c, CL Brener and YC6 T. cruzi strains.** Figure continues on the next page.

```
II_c11_10_NEL        ---------------GTGCATGCTTTTTTTCTATTATATGCTTCTGTTGAGCGCCCGAGC   176
II_c7_6_NEL          ---------------GTGCATGCTTTTTTTCTATTATATGCTTCTGTTGAGCGCCCGAGC   176
II_c5_4_NEL          ---------------GTGCATGCTTTTTTTCTATTATATGCTTCTGTTGAGCGCCCGAGC   176
II_c5_4_EL           ---------------GTGCATGCTTTTTTTCTATTATATGCTTCTGTTGAGCGCCCGAGC   177
II_c15_14_YC6        ---------------GTGCATGCTTTTTTTCTATTATATGCTTCTGTTGAGCGCCCGAGC   177
II_c13_12_YC6        ---------------GTGCATGCTTTTTTTCTATTATATGCTTCTGTTGAGCGCCCGAGC   177
II_c12_11_YC6        ---------------GTGCATGCTTTTTTTCTATTATATGCTTCTGTTGAGCGCCCGAGC   177
II_c11_10_YC6        ---------------GTGCATGCTTTTTTTCTATTATATGCTTCTGTTGAGCGCCCGAGC   177
II_c10_9_YC6         ---------------GTGCATGCTTTTTTTCTATTATATGCTTCTGTTGAGCGCCCGAGC   177
II_c9_8_YC6          ---------------GTGCATGCTTTTTTTCTATTATATGCTTCTGTTGAGCGCCCGAGC   177
II_c3_2_EL           ---------------GTGCATGCTTTTTTTCTATTATATGCTTCTGTTGAGCGCCCGAGC   177
II_c14_13_YC6        ---------------GTGCATGCTTTTTTTCTATTATATGCTTCTGTTGAGCGCCCGAGC   177
II_c4_3_EL           ---------------GTGCATGCTTTTTTTCTATTATATGCTTCTGTTGAGCGCCCGAGC   177
II_c2_1_dm28c        ---------------GTGCATGCTTTTTTTCTATTATATGCTTCTGTTGAGCGCCCGAGC   177
II_c4_3_dm28c        ---------------GTGCATGCTTTTTTTCTATTATATGCTTCTGTTGAGCGCCCGAGC   177
II_c5_4_dm28c        ---------------GTGCATGCTTTTTTTCTATTATATGCTTCTGTTGAGCGCCCGAGC   177
II_c6_5_dm28c        ---------------GTGCATGCTTTTTTTCTATTATATGCTTCTGTTGAGCGCCCGAGC   177
II_c8_7_dm28c        ---------------GTGCATGCTTTTTTTCTATTATATGCTTCTGTTGAGCGCCCGAGC   177
II_c3_2_dm28c        ---------------GTGCATGCTTTTTTTCTATTATATGCTTCTGTTGAGCGCCCGAGC   177
II_c9_8_dm28c        ---------------GTGCATGCTTTTTTTCTATTATATGCTTCTGTTGAGCGCCCGAGC   177
II_c7_6_dm28c        ---------------GTGCATGCTTTTTTTCTATTATATGCTTCTGTTGAGCGCCCGAGC   177
                                      ** **  *** ***** * **************** *******

I_c8_7_EL            ATTCCCCACTGTGGCTGAACCCACCGCCGTCCCCGACTGTCACCCGACCATGCGGGGC-G   299
I_c16_15_YC6         ATTCCCCACTGTGGCTGAACCCACCGCCGTCCCCGACTGTCACCCGACCATGCGGGGCGG   298
I_c5_4_EL            ATTCCCCACTGTGGCTGAACCCACCGCCGTC-CCGACTGTCACCCGACCATGCGGGGCGG   298
I_c18_17_YC6         ATTCCCCACTGTGGCTGAACCCACCGCCGTCCCCGACTGTCACCCGACCATGCGGGGCGG   299
I_c17_16_YC6         ATTCCCCACTGTGGCTGAACCCACCGCCGTCCCCGACTGTCACCCGACCATGCGGGGCGG   299
I_c10_9_EL           ATTCCCCACTGTGGCTGAACCCACCGCCGTCCCCGACTGTCACCCGACCATGCGGGGCGG   299
I_c4_3_EL            ATTCCCCACTGTGGCTGAACCCACCGCCGTCCCCGACTGTCACCCGACCATGCGGGGCGG   299
I_c3_2_EL            ATTCCCCACTGTGGCTGAACCCACCGCCGTCCCCGACTGTCACCCGACCATGCGGGGCGG   299
I_c2_1_EL            ATTCCCCACTGTGGCTGAACCCACCGCCGTCCCCGACTGTCACCCGACCATGCGGGGCGG   299
I_c2-1_YC6           ATTCCCCACTGTGGCTGAACCCACCGCCGTCCCCGACTGTCACCCGACCATGCGGGGCGG   299
I_c3_2_YC6           ATTCCCCACTGTGGCTGAACCCACCGCCGTCCCCGACTGTCACCCGACCATGCGGGGCGG   299
I_c4_3_YC6           ATTCCCCACTGTGGCTGAACCCACCGCCGTCCCCGACTGTCACCCGACCATGCGGGGCGG   299
I_c5_4_YC6           ATTCCCCACTGTGGCTGAACCCACCGCCGTCCCCGACTGTCACCCGACCATGCGGGGCGG   299
I_c6_5_YC6           ATTCCCCACTGTGGCTGAACCCACCGCCGTCCCCGACTGTCACCCGACCATGCGGGGCGG   299
I_Yc7_6_YC6          ATTCCCCACTGTGGCTGAACCCACCGCCGTCCCCGACTGTCACCCGACCATGCGGGGCGG   299
I_c8_7_YC6           ATTCCCCACTGTGGCTGAACCCACCGCCGTCCCCGACTGTCACCCGACCATGCGGGGCGG   299
I_c9_8_YC6           ATTCCCCACTGTGGCTGAACCCACCGCCGTCCCCGACTGTCACCCGACCATGCGGGGCGG   299
I_c10_9_YC6          ATTCCCCACTGTGGCTGAACCCACCGCCGTCCCCGACTGTCACCCGACCATGCGGGGCGG   299
I_c11_10_YC6         ATTCCCCACTGTGGCTGAACCCACCGCCGTCCCCGACTGTCACCCGACCATGCGGGGCGG   299
I_c12_11_YC6         ATTCCCCACTGTGGCTGAACCCACCGCCGTCCCCGACTGTCACCCGACCATGCGGGGCGG   299
I_c13_12_YC6         ATTCCCCACTGTGGCTGAACCCACCGCCGTCCCCGACTGTCACCCGACCATGCGGGGCGG   299
I_c14_13_YC6         ATTCCCCACTGTGGCTGAACCCACCGCCGTCCCCGACTGTCACCCGACCATGCGGGGCGG   299
I_c15_14_YC6         ATTCCCCACTGTGGCTGAACCCACCGCCGTCCCCGACTGTCACCCGACCATGCGGGGCGG   299
III_c2_1_YC6         ATTCCCCACTGTGGCTGAACCCACCGCCGTCCCCGACTGTCACCCGACCATGCGGGGCGG   299
III_c3_2_YC6         ATTCCCCACTGTGGCTGAACCCACCGCCGTCCCCGACTGTCACCCGACCATGCGGGGCGG   299
III_c4_3_YC6         ATTCCCCACTGTGGCTGAACCCACCGCCGTCCCCGACTGTCACCCGACCATGCGGGGCGG   299
III_c5_4_YC6         ATTCCCCACTGTGGCTGAACCCACCGCCGTCCCCGACTGTCACCCGACCATGCGGGGCGG   299
III_c6_5_YC6         ATTCCCCACTGTGGCTGAACCCACCGCCGTCCCCGACTGTCACCCGACCATGCGGGGCGG   299
III_c7_6_YC6         ATTCCCCACTGTGGCTGAACCCACCGCCGTCCCCGACTGTCACCCGACCATGCGGGGCGG   299
I_c9_8_EL            ATTCCCCACTGTGGCTGAACCCACCGCCGTCCCCGACTGTCACCCGACCATGCGGGGCGG   299
I_c14_13_NEL         ACTCCCCACTGTGGCTGAACCCACCGCCGTCCCCGACTGTCAGCCGACCATGCGGGGCGG   297
I_c5_4_NEL           ACTCCCCACTGTGGCTGAACCCACCGCCGTCCCCGACTGTCAGCCGACCATGCGGGGCGG   298
I_c7_6_NEL           ACTCCCCACTGTGGCTGAACCCACCGCCGTC-CCGACTGTCAGCCGACCATGCGGGGCGG   298
I_c2_1_dm28c         ATTCCCCACTGTGGCTGAACCCACCGCCGTCCCCGACTGTCAGCCGACCATGCGGGGCGG   299
I_c3_2_dm28c         ATTCCCCACTGTGGCTGAACCCACCGCCGTCCCCGACTGTCAGCCGACCATGCGGGGCGG   299
I_c4_3_dm28c         ATTCCCCACTGTGGCTGAACCCACCGCCGTCCCCGACTGTCAGCCGACCATGCGGGGCGG   299
I_c5_4_dm28c         ATTCCCCACTGTGGCTGAACCCACCGCCGTCCCCGACTGTCAGCCGACCATGCGGGGCGG   299
I_c6_5_dm28c         ATTCCCCACTGTGGCTGAACCCACCGCCGTCCCCGACTGTCAGCCGACCATGCGGGGCGG   299
I_c7_6_dm28c         ATTCCCCACTGTGGCTGAACCCACCGCCGTCCCCGACTGTCAGCCGACCATGCGGGGCGG   299
I_c8_7_dm28c         ATTCCCCACTGTGGCTGAACCCACCGCCGTCCCCGACTGTCAGCCGACCATGCGGGGCGG   299
I_c9_8_dm28c         ATTCCCCACTGTGGCTGAACCCACCGCCGTCCCCGACTGTCAGCCGACCATGCGGGGCGG   299
I_c10_9_dm28c        ATTCCCCACTGTGGCTGAACCCACCGCCGTCCCCGACTGTCAGCCGACCATGCGGGGCGG   299
I_c11_10_dm28c       ATTCCCCACTGTGGCTGAACCCACCGCCGTCCCCGACTGTCAGCCGACCATGCGGGGCGG   299
I_c12_11_dm28c       ATTCCCCACTGTGGCTGAACCCACCGCCGTCCCCGACTGTCAGCCGACCATGCGGGGCGG   299
```

Figure S2 - **Alignment of intergenic sequences among tandem cruzipain copies from Dm28c, CL Brener and YC6 *T. cruzi* strains.** Figure continues on the next page.

```
I_c13_12_dm28c      ATTCCCCACTGTGGCTGAACCCACCGCCGTCCCCGACTGTCAGCCGACCATGCGGGGCGG   299
I_c14_13_dm28c      ATTCCCCACTGTGGCTGAACCCACCGCCGTCCCCGACTGTCAGCCGACCATGCGGGGCGG   299
I_c15_14_dm28c      ATTCCCCACTGTGGCTGAACCCACCGCCGTCCCCGACTGTCAGCCGACCATGCGGGGCGG   299
I_c2_1_NEL          ACTCCCCACTGTGGCTGAACCCACCGCCGTCCCCGACTGTCAGCCGACCATGCGGGGCGG   298
I_c13_12_NEL        ACTCCCCACTGTGGCTGAACCCACCGCCGTCCCCGACTGTCAGCCGACCATGCGGGGCGG   298
I_c6_5_EL           ACTCCCCACTGTGGCTGAACCCACCGCCGTCCCCGACTGTCAGCCGACCATGCGGGGCGG   299
I_c15_14_NEL        ACTCCCCACTGTGGCTGAACCCACCGCCGTCCCCGACTGTCAGCCGACCATGCGGGGCGG   299
I_c12_11_NEL        ACTCCCCACTGTGGCTGAACCCACCGCCGTCCCCGACTGTCAGCCGACCATGCGGGGCGG   299
I_c11_10_NEL        ACTCCCCACTGTGGCTGAACCCACCGCCGTCCCCGACTGTCAGCCGACCATGCGGGGCGG   299
I_c9_8_NEL          ACTCCCCACTGTGGCTGAACCCACCGCCGTCCCCGACTGTCAGCCGACCATGCGGGGCGG   299
I_c8_7_NEL          ACTCCCCACTGTGGCTGAACCCACCGCCGTCCCCGACTGTCAGCCGACCATGCGGGGCGG   299
I_c3_2_NEL          ACTCCCCACTGTGGCTGAACCCACCGCCGTCCCCGACTGTCAGCCGACCATGCGGGGCGG   299
I_c17_16_NEL        ACTCCCCACTGTGGCTGAACCCACCGCCGTCCCCGACTGTCAGCCGACCATGCGGGGCGG   299
I_c18_17_NEL        ACTCCCCACTGTGGCTGAACCCACCGCCGTCCCCGACTGTCAGCCGACCATGCGGGGCGG   299
I_c16_15_NEL        ACTCCCCACTGTGGCTGAACCCACCGCCGTCCCCGACTGTCAGCCGACCATGCGGGGCGG   299
I_c10_9_NEL         ACTCCCCACTGTGGCTGAACCCACCGCCGTCCCCGACTGTCAGCCGACCATGCGGGGCGG   299
I_c4_3_NEL          ACTCCCCACTGTGGCTGAACCCACCGCCGTCCCCGACTGTCAGCCGACCATGCGGGGCGG   299
II_c8_7_NEL         ACTCCCCACTGTGGCTGAACCCACCGCCGTCCCCGACTGTCAGCCGACCATGCGGGGCGG   236
II_c6_5_NEL         ACTCCCCACTGTGGCTGAACCCACCGCCGTCCCCGACTGTCAGCCGACCATGCGGGGCGG   236
II_c10_9_NEL        ACTCCCCACTGTGGCTGAACCCACCGCCGTCCCCGACTGTCAGCCGACCATGCGGGGCGG   236
II_c9_8_NEL         ACTCCCCACTGTGGCTGAACCCACCGCCGTCCCCGACTGTCAGCCGACCATGCGGGGCGG   236
II_c4_3_NEL         ATTCCCCACTGTGGCTGAACCCACCGCCGTCCCCGACTGTCAGCCGACCATGCGGGGCGG   236
II_c11_10_NEL       ATTCCCCACTGTGGCTGAACCCACCGCCGTCCCCGACTGTCAGCCGACCATGCGGGGCGG   236
II_c7_6_NEL         ATTCCCCACTGTGGCTGAACCCACCGCCGTCCCCGACTGTCAGCCGACCATGCGGGGCGG   236
II_c5_4_NEL         ATTCCCCACTGTGGCTGAACCCACCGCCGTCCCCGACTGTCAGCCGACCATGCGGGGCGG   236
II_c5_4_EL          ATTCCCCACTGTGGCTGAGCCCACCGCCGTCCCCGACTGTCACCCGACCATGCGGGGCGG   237
II_c15_14_YC6       ATTCCCCACTGTGGCTGAGCCCACCGCCGTCCCCGACTGTCACCCGACCATGCGGGGCGG   237
II_c13_12_YC6       ATTCCCCACTGTGGCTGAGCCCGCCGCCGTCCCCGACTGTCACCCGACCATGCGGGGCGG   237
II_c12_11_YC6       ATTCCCCACTGTGGCTGAGCCCGCCGCCGTCCCCGACTGTCACCCGACCATGCGGGGCGG   237
II_c11_10_YC6       ATTCCCCACTGTGGCTGAGCCCGCCGCCGTCCCCGACTGTCACCCGACCATGCGGGGCGG   237
II_c10_9_YC6        ATTCCCCACTGTGGCTGAGCCCGCCGCCGTCCCCGACTGTCACCCGACCATGCGGGGCGG   237
II_c9_8_YC6         ATTCCCCACTGTGGCTGAGCCCGCCGCCGTCCCCGACTGTCACCCGACCATGCGGGGCGG   237
II_c3_2_EL          ATTCCCCACTGTGGCTGAGCCCGCCGCCGTCCCCGACTGTCACCCGACCATGCGGGGCGG   237
II_c14_13_YC6       ATTCCCCACTGTGGCTGAGCCCGCCGCCGTCCCCGACTGTCACCCGACCATGCGGGGCGG   237
II_c4_3_EL          ATTCCCCACTGTGGCTGAGCCCGCCGCCGTCCCCGACTGTCACCCGACCATGCGGGGCGG   237
II_c2_1_dm28c       ATTCCCCACTGTGGCTGAACCCACCGCCGTCCCCGACTGTCAGCCGACCATGCGGGGCGG   237
II_c4_3_dm28c       ATTCCCCACTGTGGCTGAACCCACCGCCGTCCCCGACTGTCAGCCGACCATGCGGGGCGG   237
II_c5_4_dm28c       ATTCCCCACTGTGGCTGAACCCACCGCCGTCCCCGACTGTCAGCCGACCATGCGGGGCGG   237
II_c6_5_dm28c       ATTCCCCACTGTGGCTGAACCCACCGCCGTCCCCGACTGTCAGCCGACCATGCGGGGCGG   237
II_c8_7_dm28c       ATTCCCCACTGTGGCTGAACCCACCGCCGTCCCCGACTGTCAGCCGACCATGCGGGGCGG   237
II_c3_2_dm28c       ATTCCCCACTGTGGCTGAACCCACCGCCGTCCCCGACTGTCAGCCGACCATGCGGGGCGG   237
II_c9_8_dm28c       ATTCCCCACTGTGGCTGAACCCACCGCCGTCCCCGACTGTCAGCCGACCATGCGGGGCGG   237
II_c7_6_dm28c       ATTCCCCACTGTGGCTGAACCCACCGCCGTCCCCGACTGTCAGCCGACCATGCGGGGCGG   237
                    * **************** *** ******** ********** *************** *

I_c8_7_EL           GGTGGCGTGGCAACGCTCCTTTGCCGCGGCGTGCACATACACGCGCGCATGCTTCTTCTC   359
I_c16_15_YC6        GGTGGCGTGGCAACGCTCCTTTGCCGCGGCGTGCACATACACGCGCGCATGCTTCTTCTC   358
I_c5_4_EL           GGTGGCGTGGCAACGCTCCTTTGCCGCGGCGTGCACATACACGCGCGCATGCTTCTTCTC   358
I_c18_17_YC6        GGTGGCGTGGCAACGCTCCTTTGCCGCGGCGTGCACATACACGCGCGCATGCTTCTTCTC   359
I_c17_16_YC6        GGTGGCGTGGCAACGCTCCTTTGCCGCGGCGTGCACATACACGCGCGCATGCTTCTTCTC   359
I_c10_9_EL          GGTGGCGTGGCAACGCTCCTTTGCCGCGGCGTGCACATACACGCGCGCATGCTTCTTCTC   359
I_c4_3_EL           GGTGGCGTGGCAACGCTCCTTTGCCGCGGCGTGCACATACACGCGCGCATGCTTCTTCTC   359
I_c3_2_EL           GGTGGCGTGGCAACGCTCCTTTGCCGCGGCGTGCACATACACGCGCGCATGCTTCTTCTC   359
I_c2_1_EL           GGTGGCGTGGCAACGCTCCTTTGCCGCGGCGTGCACATACACGCGCGCATGCTTCTTCTC   359
I_c2-1_YC6          GGTGGCGTGGCAACGCTCCTTTGCCGCGGCGTGCACATACACGCGCGCATGCTTCTTCTC   359
I_c3_2_YC6          GGTGGCGTGGCAACGCTCCTTTGCCGCGGCGTGCACATACACGCGCGCATGCTTCTTCTC   359
I_c4_3_YC6          GGTGGCGTGGCAACGCTCCTTTGCCGCGGCGTGCACATACACGCGCGCATGCTTCTTCTC   359
I_c5_4_YC6          GGTGGCGTGGCAACGCTCCTTTGCCGCGGCGTGCACATACACGCGCGCATGCTTCTTCTC   359
I_c6_5_YC6          GGTGGCGTGGCAACGCTCCTTTGCCGCGGCGTGCACATACACGCGCGCATGCTTCTTCTC   359
I_Yc7_6_YC6         GGTGGCGTGGCAACGCTCCTTTGCCGCGGCGTGCACATACACGCGCGCATGCTTCTTCTC   359
I_c8_7_YC6          GGTGGCGTGGCAACGCTCCTTTGCCGCGGCGTGCACATACACGCGCGCATGCTTCTTCTC   359
I_c9_8_YC6          GGTGGCGTGGCAACGCTCCTTTGCCGCGGCGTGCACATACACGCGCGCATGCTTCTTCTC   359
I_c10_9_YC6         GGTGGCGTGGCAACGCTCCTTTGCCGCGGCGTGCACATACACGCGCGCATGCTTCTTCTC   359
I_c11_10_YC6        GGTGGCGTGGCAACGCTCCTTTGCCGCGGCGTGCACATACACGCGCGCATGCTTCTTCTC   359
I_c12_11_YC6        GGTGGCGTGGCAACGCTCCTTTGCCGCGGCGTGCACATACACGCGCGCATGCTTCTTCTC   359
I_c13_12_YC6        GGTGGCGTGGCAACGCTCCTTTGCCGCGGCGTGCACATACACGCGCGCATGCTTCTTCTC   359
I_c14_13_YC6        GGTGGCGTGGCAACGCTCCTTTGCCGCGGCGTGCACATACACGCGCGCATGCTTCTTCTC   359
```

Figure S2 - **Alignment of intergenic sequences among tandem cruzipain copies from Dm28c, CL Brener and YC6 *T. cruzi* strains.** Figure continues on the next page.

```
I_c15_14_YC6      GGTGGCGTGGCAACGCTCCTTTGCCGCGGCGTGCACATACACGCGCGCATGCTTCTTCTC   359
III_c2_1_YC6      GGTGGCGTGGCAACGCTCCTTTGCCGCGGCGTGCACATACACGCGCGCATGCTTCTTCTC   359
III_c3_2_YC6      GGTGGCGTGGCAACGCTCCTTTGCCGCGGCGTGCACATACACGCGCGCATGCTTCTTCTC   359
III_c4_3_YC6      GGTGGCGTGGCAACGCTCCTTTGCCGCGGCGTGCACATACACGCGCGCATGCTTCTTCTC   359
III_c5_4_YC6      GGTGGCGTGGCAACGCTCCTTTGCCGCGGCGTGCACATACACGCGCGCATGCTTCTTCTC   359
III_c6_5_YC6      GGTGGCGTGGCAACGCTCCTTTGCCGCGGCGTGCACATACACGCGCGCATGCTTCTTCTC   359
III_c7_6_YC6      GGTGGCGTGGCAACGCTCCTTTGCCGCGGCGTGCACATACACGCGCGCATGCTTCTTCTC   359
I_c9_8_EL         GGTGGCGTGGCAACGCTCCTTTGCCGCGGCGTGCACATACACGCGCGCATGCTTCTTCTC   359
I_c14_13_NEL      AGTGGCGTGGCAACGCTCCTTTGCCGCGGCGTGCACATACACGCGCGCATGCTCCTTCTC   357
I_c5_4_NEL        AGTGGCGTGGCAACGCTCCTTTGCCGCGGCGTGCACATACACGCGCGCATGCTCCTTCTC   358
I_c7_6_NEL        AGTGGCGTGGCAACGCTCCCTTGCCGCGGCGTGCACATACACGCGCGCATGCTCCTTCTC   358
I_c2_1_dm28c      AGTGGCGTGGCAACGCTCCTTTGCCGCGGCGTGCACATACACGCGCGCATGCTTCTTCTC   359
I_c3_2_dm28c      AGTGGCGTGGCAACGCTCCTTTGCCGCGGCGTGCACATACACGCGCGCATGCTTCTTCTC   359
I_c4_3_dm28c      AGTGGCGTGGCAACGCTCCTTTGCCGCGGCGTGCACATACACGCGCGCATGCTTCTTCTC   359
I_c5_4_dm28c      AGTGGCGTGGCAACGCTCCTTTGCCGCGGCGTGCACATACACGCGCGCATGCTTCTTCTC   359
I_c6_5_dm28c      AGTGGCGTGGCAACGCTCCTTTGCCGCGGCGTGCACATACACGCGCGCATGCTTCTTCTC   359
I_c7_6_dm28c      AGTGGCGTGGCAACGCTCCTTTGCCGCGGCGTGCACATACACGCGCGCATGCTTCTTCTC   359
I_c8_7_dm28c      AGTGGCGTGGCAACGCTCCTTTGCCGCGGCGTGCACATACACGCGCGCATGCTTCTTCTC   359
I_c9_8_dm28c      AGTGGCGTGGCAACGCTCCTTTGCCGCGGCGTGCACATACACGCGCGCATGCTTCTTCTC   359
I_c10_9_dm28c     AGTGGCGTGGCAACGCTCCTTTGCCGCGGCGTGCACATACACGCGCGCATGCTTCTTCTC   359
I_c11_10_dm28c    AGTGGCGTGGCAACGCTCCTTTGCCGCGGCGTGCACATACACGCGCGCATGCTTCTTCTC   359
I_c12_11_dm28c    AGTGGCGTGGCAACGCTCCTTTGCCGCGGCGTGCACATACACGCGCGCATGCTTCTTCTC   359
I_c13_12_dm28c    AGTGGCGTGGCAACGCTCCTTTGCCGCGGCGTGCACATACACGCGCGCATGCTTCTTCTC   359
I_c14_13_dm28c    AGTGGCGTGGCAACGCTCCTTTGCCGCGGCGTGCACATACACGCGCGCATGCTTCTTCTC   359
I_c15_14_dm28c    AGTGGCGTGGCAACGCTCCTTTGCCGCGGCGTGCACATACACGCGCGCATGCTTCTTCTC   359
I_c2_1_NEL        AGTGGCGTGGCAACGCTCCTTTGCCGCGGCGTGCACATACACGCGCGCATGCTCCTTCTC   358
I_c13_12_NEL      AGTGGCGTGGCAACGCTCCTTTGCCGCGGCGTGCACATACACGCGCGCATGCTCCTTCTC   358
I_c6_5_EL         AGTGGCGTGGCAACGCTCCTTTGCCGCGGCGTGCACATACACGCGCGCATGCTCCTTCTC   359
I_c15_14_NEL      AGTGGCGTGGCAACGCTCCTTTGCCGCGGCGTGCACATACACGCGCGCATGCTCCTTCTC   359
I_c12_11_NEL      AGTGGCGTGGCAACGCTCCTTTGCCGCGGCGTGCACATACACGCGCGCATGCTCCTTCTC   359
I_c11_10_NEL      AGTGGCGTGGCAACGCTCCTTTGCCGCGGCGTGCACATACACGCGCGCATGCTCCTTCTC   359
I_c9_8_NEL        AGTGGCGTGGCAACGCTCCTTTGCCGCGGCGTGCACATACACGCGCGCATGCTCCTTCTC   359
I_c8_7_NEL        AGTGGCGTGGCAACGCTCCTTTGCCGCGGCGTGCACATACACGCGCGCATGCTCCTTCTC   359
I_c3_2_NEL        AGTGGCGTGGCAACGCTCCTTTGCCGCGGCGTGCACATACACGCGCGCATGCTCCTTCTC   359
I_c17_16_NEL      AGTGGCGTGGCAACGCTCCTTTGCCGCGGCGTGCACATACACGCGCGCATGCTCCTTCTC   359
I_c18_17_NEL      AGTGGCGTGGCAACGCTCCTTTACCGCGGCGTGCACATACACGCGCGCATGCTCCTTCTC   359
I_c16_15_NEL      AGTGGCGTGGCAACGCTCCTTTACCGCGGCGTGCACATACACGCGCGCATGCTCCTTCTC   359
I_c10_9_NEL       AGTGGCGTGGCAACGCTCCTTTGCCGCGGCGTGCACATACACGCGCGCATGCTCCTTCTC   359
I_c4_3_NEL        AGTGGCGTGGCAACGCTCCTTTGCCGCGGCGTGCACATACACGCGCGCATGCTCCTTCTC   359
II_c8_7_NEL       AGTGGCGTGGCAACGCTCCTTTGCCGCGGCGTGCACATACACGCGCGCATGCTCCTTCTC   296
II_c6_5_NEL       AGTGGCGTGGCGACGCTCCTTTGCCGTGGCGTGCACATACACGCGCGCATGCTCCTTCTC   296
II_c10_9_NEL      AGTGGCGTGGCAACGCTCCTTTGCCGCGGCGTGCACATACACGCGCGCATGCTCCTTCTC   296
II_c9_8_NEL       AGTGGCGTGGCAACGCTCCTTTGCCGTGGCGTGCACATACACGCGCGCATGCTCCTTCTC   296
II_c4_3_NEL       AGTGGCGTGGCAACGCTCCTTTGCCGTGGCGTGCACATACACGCGCGCATGCTCCTTCTC   296
II_c11_10_NEL     AGTGGCGTGGCGACGCTCCTTTGCCGTGGCGTGCACATACACGCGCGCATGCTTCTTCTC   296
II_c7_6_NEL       AGTGGCGTGGCGACGCTCCTTTGCCGTGGCGTGCACATACACGCGCGCATGCTCCTTCTC   296
II_c5_4_NEL       AGTGGCGTGGCGACGCTCCTTTGCCGTGGCGTGCACATACACGCGCGCATGCTCCTTCTC   296
II_c5_4_EL        AGTGGCGTGGCAACGCTCCTTTGCCGCGGCGTGCACATACACGCGCGCATGCTTCTTCTC   297
II_c15_14_YC6     AGTGGCGTGGCAACGCTCCTTTGCCGCGGCGTGCACATACACGCGCGCATGCTTCTTCTC   297
II_c13_12_YC6     AGTGGCGTGGCAACGCTCCTTTGCCGCGGCGTGCACATACACGCGCGCATGCTTCTTCTC   297
II_c12_11_YC6     AGTGGCGTGGCAACGCTCCTTTGCCGCGGCGTGCACATACACGCGCGCATGCTTCTTCTC   297
II_c11_10_YC6     AGTGGCGTGGCAACGCTCCTTTGCCGCGGCGTGCACATACACGCGCGCATGCTTCTTCTC   297
II_c10_9_YC6      AGTGGCGTGGCAACGCTCCTTTGCCGCGGCGTGCACATACACGCGCGCATGCTTCTTCTC   297
II_c9_8_YC6       AGTGGCGTGGCAACGCTCCTTTGCCGCGGCGTGCACATACACGCGCGCATGCTTCTTCTC   297
II_c3_2_EL        AGTGGCGTGGCAACGCTCCTTTGCCGCGGCGTGCACATACACGCGCGCATGCTTCTTCTC   297
II_c14_13_YC6     AGTGGCGTGGCAACGCTCCTTTGCCGCGGCGTGCACATACACGCGCGCATGCTTCTTCTC   297
II_c4_3_EL        AGTGGCGTGGCAACGCTCCTTTGCCGCGGCGTGCACATGCACGCGCGCATGCTTCTTCTC   297
II_c2_1_dm28c     AGTGGCGTGGCAACGCTCCTTTGCCGCGGCGTGCACATACACGCGCGCATGCTTCTTCTC   297
II_c4_3_dm28c     AGTGGCGTGGCAACGCTCCTTTGCCGCGGCGTGCACATACACGCGCGCATGCTTCTTCTC   297
II_c5_4_dm28c     AGTGGCGTGGCAACGCTCCTTTGCCGCGGCGTGCACATACACGCGCGCATGCTTCTTCTC   297
II_c6_5_dm28c     AGTGGCGTGGCAACGCTCCTTTGCCGCGGCGTGCACATACACGCGCGCATGCTTCTTCTC   297
II_c8_7_dm28c     AGTGGCGTGGCAACGCTCCTTTGCCGCGGCGTGCACATACACGCGCGCATGCTTCTTCTC   297
II_c3_2_dm28c     AGTGGCGTGGCAACGCTCCTTTGCCGCGGCGTGCACATACACGCGCGCATGCTTCTTCTC   297
II_c9_8_dm28c     AGTGGCGTGGCAACGCTCCTTTGCCGCGGCGTGCACATACACGCGCGCATGCTTCTTCTC   297
II_c7_6_dm28c     AGTGGCGTGGCAACGCTCCTTTGCCGCGGCGTGCACATACACGCGCGCATGCTTCTTCTC   297
                   ********** ******* ** *** *********** ************** ******
```

Figure S2 - **Alignment of intergenic sequences among tandem cruzipain copies from Dm28c, CL Brener and YC6 *T. cruzi* strains.** Figure continues on the next page.

```
I_c8_7_EL          TCCACGTCAACGCCCGCACACCACCACAGTCTGAAGGTGACGCTGTTGAAGGCACACAAG   419
I_c16_15_YC6       TCCACGTCAACGCCCGCACACCACCACAGTCTGAAGGTGACGCTGTTGAAGGCACACAAG   418
I_c5_4_EL          TCCACGTCAACGCCCGCACACCACCACAGTCTGAAGGTGACGCTGTTGAAGGCACACAAG   418
I_c18_17_YC6       TCCACGTCAACGCCCGCGCACCACCACAGTCTGAAGGTGACGCTGTTGAAGGCACACAAG   419
I_c17_16_YC6       TCCACGTCAACGCCCGCACACCACCACAGTCTGAAGGTGACGCTGTTGAAGGCACACAAG   419
I_c10_9_EL         TCCACGTCAACGCCCGCACACCACCACAGTCTGAAGGTGACGCTGTTGAAGGCACACAAG   419
I_c4_3_EL          TCCACGTCAACGCCCGCACACCACCACAGTCTGAAGGTGACGCTGTTGAAGGCACACAAG   419
I_c3_2_EL          TCCACGTCAACGCCCGCACACCACCACAGTCTGAAGGTGACGCTGTTGAAGGCACACAAG   419
I_c2_1_EL          TCCACGTCAACGCCCGCACACCACCACAGTCTGAAGGTGACGCTGTTGAAGGCACACAAG   419
I_c2-1_YC6         TCCACGTCAACGCCCGCACACCACCACAGTCTGAAGGTGACGCTGTTGAAGGCACACAAG   419
I_c3_2_YC6         TCCACGTCAACGCCCGCACACCACCACAGTCTGAAGGTGACGCTGTTGAAGGCACACAAG   419
I_c4_3_YC6         TCCACGTCAACGCCCGCACACCACCACAGTCTGAAGGTGACGCTGTTGAAGGCACACAAG   419
I_c5_4_YC6         TCCACGTCAACGCCCGCACACCACCACAGTCTGAAGGTGACGCTGTTGAAGGCACACAAG   419
I_c6_5_YC6         TCCACGTCAACGCCCGCACACCACCACAGTCTGAAGGTGACGCTGTTGAAGGCACACAAG   419
I_Yc7_6_YC6        TCCACGTCAACGCCCGCACACCACCACAGTCTGAAGGTGACGCTGTTGAAGGCACACAAG   419
I_c8_7_YC6         TCCACGTCAACGCCCGCACACCACCACAGTCTGAAGGTGACGCTGTTGAAGGCACACAAG   419
I_c9_8_YC6         TCCACGTCAACGCCCGCACACCACCACAGTCTGAAGGTGACGCTGTTGAAGGCACACAAG   419
I_c10_9_YC6        TCCACGTCAACGCCCGCACACCACCACAGTCTGAAGGTGACGCTGTTGAAGGCACACAAG   419
I_c11_10_YC6       TCCACGTCAACGCCCGCACACCACCACAGTCTGAAGGTGACGCTGTTGAAGGCACACAAG   419
I_c12_11_YC6       TCCACGTCAACGCCCGCACACCACCACAGTCTGAAGGTGACGCTGTTGAAGGCACACAAG   419
I_c13_12_YC6       TCCACGTCAACGCCCGCACACCACCACAGTCTGAAGGTGACGCTGTTGAAGGCACACAAG   419
I_c14_13_YC6       TCCACGTCAACGCCCGCACACCACCACAGTCTGAAGGTGACGCTGTTGAAGGCACACAAG   419
I_c15_14_YC6       TCCACGTCAACGCCCGCACACCACCACAGTCTGAAGGTGACGCTGTTGAAGGCACACAAG   419
III_c2_1_YC6       TCCACGTCAACGCCCGCACACCACCACAGTCTGAAGGTGACGCTGTTGAAGGCACACAAG   419
III_c3_2_YC6       TCCACGTCAACGCCCGCACACCACCACAGTCTGAAGGTGACGCTGTTGAAGGCACACAAG   419
III_c4_3_YC6       TCCACGTCAACGCCCGCACACCACCACAGTCTGAAGGTGACGCTGTTGAAGGCACACAAG   419
III_c5_4_YC6       TCCACGTCAACGCCCGCACACCACCACAGTCTGAAGGTGACGCTGTTGAAGGCACACAAG   419
III_c6_5_YC6       TCCACGTCAACGCCCGCACACCACCACAGTCTGAAGGTGACGCTGTTGAAGGCACACAAG   419
III_c7_6_YC6       TCCACGTCAACGCCCGCACACCACCACAGTCTGAAGGTGACGCTGTTGAAGGCACACAAG   419
I_c9_8_EL          TCCACGTCAACGCCCGCACACCACCACAGTCTGAAGGTGACGCTGTTGAAGGCACACAAG   419
I_c14_13_NEL       TCCACGTCAACGCCCGCACACCACCACAGTCTGAAGGTGACGCTGTTGAAGGCACANGAA   417
I_c5_4_NEL         TCCACGTCAACGCCCGCACACCACCACAGTCTGAAGGTGACGCTGTTGAAGGCACACAAG   418
I_c7_6_NEL         TCCACGTCAACGCCCGCACACCACCACAGTCTGAAGGTGACGCTGTTGAAGGCACATAAG   418
I_c2_1_dm28c       TCCACGTCAACGCCCGCACACCACCACAGTCTGAAGGTGACGCTGTTGAAGGCACACAAG   419
I_c3_2_dm28c       TCCACGTCAACGCCCGCACACCACCACAGTCTGAAGGTGACGCTGTTGAAGGCACACAAG   419
I_c4_3_dm28c       TCCACGTCAACGCCCGCACACCACCACAGTCTGAAGGTGACGCTGTTGAAGGCACACAAG   419
I_c5_4_dm28c       TCCACGTCAACGCCCGCACACCACCACAGTCTGAAGGTGACGCTGTTGAAGGCACACAAG   419
I_c6_5_dm28c       TCCACGTCAACGCCCGCACACCACCACAGTCTGAAGGTGACGCTGTTGAAGGCACACAAG   419
I_c7_6_dm28c       TCCACGTCAACGCCCGCACACCACCACAGTCTGAAGGTGACGCTGTTGAAGGCACACAAG   419
I_c8_7_dm28c       TCCACGTCAACGCCCGCACACCACCACAGTCTGAAGGTGACGCTGTTGAAGGCACACAAG   419
I_c9_8_dm28c       TCCACGTCAACGCCCGCACACCACCACAGTCTGAAGGTGACGCTGTTGAAGGCACACAAG   419
I_c10_9_dm28c      TCCACGTCAACGCCCGCACACCACCACAGTCTGAAGGTGACGCTGTTGAAGGCACACAAG   419
I_c11_10_dm28c     TCCACGTCAACGCCCGCACACCACCACAGTCTGAAGGTGACGCTGTTGAAGGCACACAAG   419
I_c12_11_dm28c     TCCACGTCAACGCCCGCACACCACCACAGTCTGAAGGTGACGCTGTTGAAGGCACACAAG   419
I_c13_12_dm28c     TCCACGTCAACGCCCGCACACCACCACAGTCTGAAGGTGACGCTGTTGAAGGCACACAAG   419
I_c14_13_dm28c     TCCACGTCAACGCCCGCACACCACCACAGTCTGAAGGTGACGCTGTTGAAGGCACACAAG   419
I_c15_14_dm28c     TCCACGTCAACGCCCGCACACCACCACAGTCTGAAGGTGACGCTGTTGAAGGCACACAAG   419
I_c2_1_NEL         TCCACGTCAACGCCCGCACACCACCACAGTCTGAAGGTGACGCTGTTGAAGGCACACAAG   418
I_c13_12_NEL       TCCACGTCAACGCCCGCACACCACCACAGTCTGAAGGTGACGCTGTTGAAGGCACATAAG   418
I_c6_5_EL          TCCACGTCAACGCCCGCACACCACCACAGTCTGAAGGTGACGCTGTTGAAGGCACATAAG   419
I_c15_14_NEL       TCCACGTCAACGCCCGCACACCACCACAGTCTGAAGGTGACGCTGTTGAAGGCACATAAG   419
I_c12_11_NEL       TCCACGTCAACGCCCGCACACCACCACAGTCTGAAGGTGACGCTGTTGAAGGCACATAAG   419
I_c11_10_NEL       TCCACGTCAACGCCCGCACACCACCACAGTCTGAAGGTGACGCTGTTGAAGGCACATAAG   419
I_c9_8_NEL         TCCACGTCAACGCCCGCACACCACCACAGTCTGAAGGTGACGCTGTTGAAGGCACATAAG   419
I_c8_7_NEL         TCCACGTCAACGCCCGCACACCACCACAGTCTGAAGGTGACGCTGTTGAAGGCACATAAG   419
I_c3_2_NEL         TCCACGTCAACGCCCGCACACCACCACAGTCTGAAGGTGACGCTGTTGAAGGCACATAAG   419
I_c17_16_NEL       TCCACGTCAACGCCCGCACACCACCACAGTCTGAAGGTGACGCTGTTGAAGGCACATAAG   419
I_c18_17_NEL       TCCACGTCAACGCCCGCACACCACCACAGTCTGAAGGTGACGCTGTTGAAGGCACACAAG   419
I_c16_15_NEL       TCCACGTCAACGCCCGCACACCACCACAGTCTGAAGGTGACGCTGTTGAAGGCACACAAG   419
I_c10_9_NEL        TCCACGTCAACGCCCGCACACCACCACAGTCTGAAGGTGACGCTGTTGAAGGCACACAAG   419
I_c4_3_NEL         TCCACGTCAACGCCCGCACACCACCACAGTCTGAAGGTGACGCTGTTGAAGGCACACAAG   419
II_c8_7_NEL        TCCACGTCAACGCCCGCACACCACCACAGTCTGAAGGTGACGCTGTTGAAGGCACACAAG   356
II_c6_5_NEL        TCCACGTCAACGCCCGCACACCACCACAGTCTGAAGGTGACGCTGTTGAAGGCACACAAG   356
II_c10_9_NEL       TCCACGTCAACGCCCGCACACCACCACAGTCTGAAGGTGACGCTGTTGAAGGCACACAAG   356
II_c9_8_NEL        TCCACGTCAACGCCCGCACACCACCACAGTCTGAAGGTGACGCTGTTGAAGGCACACAAG   356
II_c4_3_NEL        TCCACGTCAACGCCCGCACACCACCACAGTCTGAAGGTGACGCTGTTGAAGGCACACAAG   356
II_c11_10_NEL      TCCACGTCAACGCCCGCACACCACCACAGTCTGAAGGTGACGCTGTTGAAGGCACACAAG   356
```

Figure S2 - **Alignment of intergenic sequences among tandem cruzipain copies from Dm28c, CL Brener and YC6 *T. cruzi* strains.** Figure continues on the next page.

```
II_c7_6_NEL         TCCACGTCAACGCCCGCACACCACCACAGTCTGAAGGTGACGCTGTTGAAGGCACACAAG  356
II_c5_4_NEL         TCCACGTCAACGCCCGCACACCACCACAGTCTGAAGGTGACGCTGTTGAAGGCACACAAG  356
II_c5_4_EL          TCCACGTCAACGCCCGCACACCACCACAGTCTGAAGGTGACGCTGTTGAAGGCACACAAG  357
II_c15_14_YC6       TCCACGTCAACGCCCGCACACCACCACAGTCTGAAGGTGACGCTGTTGAAGGCACACAAG  357
II_c13_12_YC6       TCCACGTCAACGCCCGCGCACCACCACAGTCTGAAGGTGACGCTGTTGAAGGCACACAAG  357
II_c12_11_YC6       TCCACGTCAACGCCCGCGCACCACCACAGTCTGAAGGTGACGCTGTTGAAGGCACACAAG  357
II_c11_10_YC6       TCCACGTCAACGCCCGCGCACCACCACAGTCTGAAGGTGACGCTGTTGAAGGCACACAAG  357
II_c10_9_YC6        TCCACGTCAACGCCCGCGCACCACCACAGTCTGAAGGTGACGCTGTTGAAGGCACACAAG  357
II_c9_8_YC6         TCCACGTCAACGCCCGCGCACCACCACAGTCTGAAGGTGACGCTGTTGAAGGCACACAAG  357
II_c3_2_EL          TCCACGTCAACGCCCGCGCACCACCACAGTCTGAAGGTGACGCTGTTGAAGGCACACAAG  357
II_c14_13_YC6       TCCACGTCAACGCCCGCGCACCACCACAGTCTGAAGGTGACGCTGTTGAAGGCACACAAG  357
II_c4_3_EL          TCCACGTCAACGCCCGCGCACCACCACAGTCTGAAGGTGACGCTGTTGAAGGCACACAAG  357
II_c2_1_dm28c       TCCACGTCAACGCCCGCACACCACCACAGTCTGAAGGTGACGCTGTTGAAGGCACACAAG  357
II_c4_3_dm28c       TCCACGTCAACGCCCGCACACCACCACAGTCTGAAGGTGACGCTGTTGAAGGCACACAAG  357
II_c5_4_dm28c       TCCACGTCAACGCCCGCACACCACCACAGTCTGAAGGTGACGCTGTTGAAGGCACACAAG  357
II_c6_5_dm28c       TCCACGTCAACGCCCGCACACCACCACAGTCTGAAGGTGACGCTGTTGAAGGCACACAAG  357
II_c8_7_dm28c       TCCACGTCAACGCCCGCACACCACCACAGTCTGAAGGTGACGCTGTTGAAGGCACACAAG  357
II_c3_2_dm28c       TCCACGTCAACGCGCGCACACCACCACAGTCTGAAGGTGACGCTGTTGAAGGCACACAAG  357
II_c9_8_dm28c       TCCACGTCAACGCGCGCACACCACCACAGTCTGAAGGTGACGCTGTTGAAGGCACACAAG  357
II_c7_6_dm28c       TCCACGTCAACGCGCGCACACCACCACAGTCTGAAGGTGACGCTGTTGAAGGCAAACAAG  357
                    ************* *** ************************************ *  *

I_c8_7_EL           GAAGGAAACACACCCACACAAGCAGTAATG  449
I_c16_15_YC6        GAAGGAAACACACCCACACAAGCAGTAATG  448
I_c5_4_EL           GAAGGAAACACACCCACACAAGCAGTAATG  448
I_c18_17_YC6        GAAGGAAACACACCCACACAAGCAGTAATG  449
I_c17_16_YC6        GAAGGAAACACACCCACACAAGCAGTAATG  449
I_c10_9_EL          GAAGGAAACACACCCACACAAGCAGTAATG  449
I_c4_3_EL           GAAGGAAACACACCCACACAAGCAGTAATG  449
I_c3_2_EL           GAAGGAAACACACCCACACAAGCAGTAATG  449
I_c2_1_EL           GAAGGAAACACACCCACACAAGCAGTAATG  449
I_c2-1_YC6          GAAGGAAACACACCCACACAAGCAGTAATG  449
I_c3_2_YC6          GAAGGAAACACACCCACACAAGCAGTAATG  449
I_c4_3_YC6          GAAGGAAACACACCCACACAAGCAGTAATG  449
I_c5_4_YC6          GAAGGAAACACACCCACACAAGCAGTAATG  449
I_c6_5_YC6          GAAGGAAACACACCCACACAAGCAGTAATG  449
I_Yc7_6_YC6         GAAGGAAACACACCCACACAAGCAGTAATG  449
I_c8_7_YC6          GAAGGAAACACACCCACACAAGCAGTAATG  449
I_c9_8_YC6          GAAGGAAACACACCCACACAAGCAGTAATG  449
I_c10_9_YC6         GAAGGAAACACACCCACACAAGCAGTAATG  449
I_c11_10_YC6        GAAGGAAACACACCCACACAAGCAGTAATG  449
I_c12_11_YC6        GAAGGAAACACACCCACACAAGCAGTAATG  449
I_c13_12_YC6        GAAGGAAACACACCCACACAAGCAGTAATG  449
I_c14_13_YC6        GAAGGAAACACACCCACACAAGCAGTAATG  449
I_c15_14_YC6        GAAGGAAACACACCCACACAAGCAGTAATG  449
III_c2_1_YC6        GAAGGAAACACACCCACACAAGCAGTAATG  449
III_c3_2_YC6        GAAGGAAACACACCCACACAAGCAGTAATG  449
III_c4_3_YC6        GAAGGAAACACACCCACACAAGCAGTAATG  449
III_c5_4_YC6        GAAGGAAACACACCCACACAAGCAGTAATG  449
III_c6_5_YC6        GAAGGAAACACACCCACACAAGCAGTAATG  449
III_c7_6_YC6        GAAGGAAACACACCCACACAAGCAGTAATG  449
I_c9_8_EL           GAAGGAAACACACCCACACAAGCAGTAATG  449
I_c14_13_NEL        ------------------------------  417
I_c5_4_NEL          GAAGGAAACACACCACACAAGC-AGTAATG  447
I_c7_6_NEL          GAAGGAAACAAACCCACACAAGCAGTAATG  448
I_c2_1_dm28c        GAAGGAAACACACCCACACAAGCAGTAATG  449
I_c3_2_dm28c        GAAGGAAACACACCCACACAAGCAGTAATG  449
I_c4_3_dm28c        GAAGGAAACACACCCACACAAGCAGTAATG  449
I_c5_4_dm28c        GAAGGAAACACACCCACACAAGCAGTAATG  449
I_c6_5_dm28c        GAAGGAAACACACCCACACAAGCAGTAATG  449
I_c7_6_dm28c        GAAGGAAACACACCCACACAAGCAGTAATG  449
I_c8_7_dm28c        GAAGGAAACACACCCACACAAGCAGTAATG  449
I_c9_8_dm28c        GAAGGAAACACACCCACACAAGCAGTAATG  449
I_c10_9_dm28c       GAAGGAAACACACCCACACAAGCAGTAATG  449
I_c11_10_dm28c      GAAGGAAACACACCCACACAAGCAGTAATG  449
I_c12_11_dm28c      GAAGGAAACACACCCACACAAGCAGTAATG  449
I_c13_12_dm28c      GAAGGAAACACACCCACACAAGCAGTAATG  449
```

Figure S2 - **Alignment of intergenic sequences among tandem cruzipain copies from Dm28c, CL Brener and YC6 *T. cruzi* strains.** Figure continues on the next page.

I_c14_13_dm28c	GAAGGAAACACACCCACACAAGCAGTAATG	449
I_c15_14_dm28c	GAAGGAAACACACCCACACAAGCAGTAATG	449
I_c2_1_NEL	GAAGGAAACACACCCACACAAGCAGTAATG	448
I_c13_12_NEL	GAAGGAAACACACCCACACAAGCAGTAATG	448
I_c6_5_EL	GAAGGAAACACACCCACACAAGCAGTAATG	449
I_c15_14_NEL	GAAGGAAACACACCCACACAAGCAGTAATG	449
I_c12_11_NEL	GAAGGAAACACACCCACACAAGCAGTAATG	449
I_c11_10_NEL	GAAGGAAACACACCCACACAAGCAGTAATG	449
I_c9_8_NEL	GAAGGAAACACACCCACACAAGCAGTAATG	449
I_c8_7_NEL	GAAGGAAACACACCCACACAAGCAGTAATG	449
I_c3_2_NEL	GAAGGAAACACACCCACACAAGCAGTAATG	449
I_c17_16_NEL	GAAGGAAACACACCCACACAAGCAGTAATG	449
I_c18_17_NEL	GAAGGAAACACACCCACACAAGCAGTAATG	449
I_c16_15_NEL	GAAGGAAACACACCCACACAAGCAGTAATG	449
I_c10_9_NEL	GAAGGAAACACACCCACACAAGCAGTAATG	449
I_c4_3_NEL	GAAGGAAACACACCCACACAAGCAGTAATG	449
II_c8_7_NEL	GAAGGAAACACGCCCACACAAGCAGTAATG	386
II_c6_5_NEL	GAAGGAAACACGCCCACACAAGCAGTAATG	386
II_c10_9_NEL	GAAGGAAACACGCCCACACAAGCAGTAATG	386
II_c9_8_NEL	GAAGGAAACACGCCCACACAAGCAGTAATG	386
II_c4_3_NEL	GAAGGAAACACGCCCACACAAGCAGTAATG	386
II_c11_10_NEL	GAAGGAAACACGCCCACACAAGCAGTAATG	386
II_c7_6_NEL	GAAGGAAACACGCCCACACAAGCAGTAATG	386
II_c5_4_NEL	GAAGGAAACACGCCCACACAAGCAGTAATG	386
II_c5_4_EL	GAAGGAAACACACCCACACAAACAGTAATG	387
II_c15_14_YC6	GAAGGAAACACACCCACACAAACAGTAATG	387
II_c13_12_YC6	GAAGGAAACACACCCACACAAACAGTAATG	387
II_c12_11_YC6	GAAGGAAACACACCCACACAAGCAGTAATG	387
II_c11_10_YC6	GAAGGAAACACACCCACACAAGCAGTAATG	387
II_c10_9_YC6	GAAGGAAACACACCCACACAAACAGTAATG	387
II_c9_8_YC6	GAAGGAAACACACCCACACAAACAGTAATG	387
II_c3_2_EL	GAAGGAAACACACCCACACAAACAGTAATG	387
II_c14_13_YC6	GAAGGAAACACACCCACACAAACAGTAATG	387
II_c4_3_EL	GAAGGAAACACACCCACACAAACAGTAATG	387
II_c2_1_dm28c	GAAGGAAACACACCCACACAAGCAGTAATG	387
II_c4_3_dm28c	GAAGGAAACACACCCACACAAGCAGTAATG	387
II_c5_4_dm28c	GAAGGAAACACACCCACACAAGCAGTAATG	387
II_c6_5_dm28c	GAAGGAAACACACCCACACAAGCAGTAATG	387
II_c8_7_dm28c	GAAGGAAACACACCCACACAAGCAGTAATG	387
II_c3_2_dm28c	GAAGGAAACACACCCACACAAGCAGTAATG	387
II_c9_8_dm28c	GAAGGAAACACACCCACACAAGCAGTAATG	387
II_c7_6_dm28c	GAAGGAAACACACCCACACAAGCAGTAATG	387

Figure S2. **Alignment of intergenic sequences among tandem cruzipain copies from Dm28c, CL Brener and YC6 *T. cruzi* strains.** Alignment performed with Clustal O (1.2.4). Positions conserved in all sequences are shown in white, while positions containing single nucleotide polymorphisms are colored in gray if they are similar to the reference sequence I_c2_1_NEL (highlighted in yellow), and cyan if they differ from the reference sequence.

```
                           -122
                           ↓
czp.1.I.10_EL              MSGWARALLLAAVLVVMACLVPAATASLHAEETLTSQFAEFKQKHGRVYESAAEEAFRLS
czp.1.I.17_YC6             MSGWARALLLAAVLVVMACLVPAATASLHAEETLTSQFAEFKQKHGRVYESAAEEAFRLS
czp.1.I.9_EL               MSGWARALLLAAVLVVMACLVPAATASLHAEETLTSQFAEFKQKHGRVYESAAEEAFRLS
czp.1.I.18_YC6             MSGWARALLLAAVLVVMACLVPAATASLHAEETLTSQFAEFKQKHGRVYESAAEEAFRLS
czp.1.I.3_EL               MSGWARALLLAAVLVVMACLVPAATASLHAEETLTSQFAEFKQKHGRVYESAAEEAFRLS
czp.1.I.2_YC6              MSGWARALLLAAVLVVMACLVPAATASLHAEETLTSQFAEFKQKHGRVYESAAEEAFRLS
czp.1.I.3_dm28c            MSGWARALSLAAVLVVMACLVPAATASLHAEETLASQFAEFKQKHGRVYGSAAEEAFRLS
czp.1.I.2_dm28c            MSGWARALSLAAVLVVMACLVPAATASLHAEETLASQFAEFKQKHGRVYESAAEEAFRLS
czp.1.I.10_NEL             MSGWARALSLAAVLVVMACLVPAATASLHAEETLASQFAEFKQKHGRVYGSAAEEAFRLS
czp.1.I.8_NEL              MSGWARALSLAAVLVVMACLVPAATASLHAEETLASQFAEFKQKHGRVYESAAEEAFRLS
czp.1.I.15_NEL             MSGWARALSLAAVLVVMACLVPAATASLHAEETLASQFAEFKQKHGRVYESAAEEAFRLS
czp.1.I.17_NEL             MSGWARALSLAAVLVVMACLVPAATASLHAEETLASQFAEFKQKHGRVYESAAEEAFRLS
czp.1.I.7_NEL              MSGWARALSLAAVLVVMACLVPAATASLHAEETLASQFAEFKQKHGRVYESAAEEAFRLS
czp.1.III.3_YC6            MSGWARALLLAAVLVVMACLVPAATASLHAEETLASQFAEFKQKHGRVYESAAEEAFRLS
czp.1.III.4_YC6            MSGWARALLLAAVLVVMACLVPAATASLHAEETLASQFAEFKQKHGRVYESAAEEAFRLS
czp.1.III.7_YC6            MSGWARALLLAAVLVVMACLVPAATASLHAEETLASQFAEFKQKHGRVYESAAEEAFRLS
czp.1.III.2_YC6            MSGWARALLLAAVLVVMACLVPAATASLHAEETLASQFAEFKQKHGRVYESAAEEAFRLS
czp.2.II.3_EL              MSGWARALLLAAVLVVMACLVPAATASLHAEETLASQFAEFKQKHGRVYESAAEEAFRLS
czp.2.II.13_YC6            MSGWARALLLAAVLVVMACLVPAATASLHAEETLASQFAEFKQKHGRVYESAAEEAFRLS
czp.2.II.10_YC6            MSGWARALLLAAVLVVMACLVPAATASLHAEETLASQFAEFKQKHGRVYESAAEEAFRLS
czp.2.II.12_YC6            MSGWARALLLAAVLVVMACLVPAATASLHAEETLASQFAEFKQKHGRVYESAAEEAFRLS
czp.2.II.2_dm28c           MSGWARALSLAAVLVVMACLVPAATASLHAEETLASQFAEFKQKHGRVYGSAAEEAFRLS
czp.3.II.4_EL              MSGWARALLLAAVLVVMACLVPAATASLHAEETLASQFAEFKQKHGRVYESAAEEAFRLS
czp.3.II.14_YC6            MSGWARALLLAAVLVVMACLVPAATASLHAEETLASQFAEFKQKHGRVYESAAEEAFRLS
czp.3.II.9_NEL             MSGWARALSLAAVLVVMACLVPAATASLHAEETLASQFAEFKQKHGRVYGSAAEEAFRLS
czp.4.II.5_NEL             MSGWARALSLAAVLVVMACLVPAATASLHAEETLASQFAEFKQKHGRVYGSAAEEAFRLS
czp.4.II.7_NEL             MSGWARALSLAAVLVVMACLVPAATASQHAEETLASQFAEFKQKHGRVYGSAAEEAFRLS
czp.4.II.3_dm28c           MSGWARALSLAAVLVVMACLVPAATASLHAEETLASQFAEFKQKHGRVYGSAAEEAFRLS
                           ******** ****************** ******:************** **********

czp.1.I.10_EL              VFRENLFLARLHAAANPHATFGVTPFSDLTREEFRSRYHNGAVHFAAAQERARVPVKVEV
czp.1.I.17_YC6             VFRENLFLARLHAAANPHATFGVTPFSDLTREEFRSRYHNGAAHFAAAQERARVPVKVEV
czp.1.I.9_EL               VFRENLFLARLHAAANPHATFGVTPFSDLTREEFRSRYHNGAAHFAAAQERARVPVKVEV
czp.1.I.18_YC6             VFRENLFLARLHAAANPHATFGVTPFSDLTREEFRSRYHNGAAHFAAAQERARVPVKVEV
czp.1.I.3_EL               VFRENLFLARLHAAANPHATFGVTPFSDLTREEFRSRYHNGAAHFAAAQERARVPVKVEV
czp.1.I.2_YC6              VFRENLFLARLHAAANPHATFGVTPFSDLTREEFRSRYHNGAAHFAAAQERARVPVKVEV
czp.1.I.3_dm28c            VFRENLFLARLHAAANPHATFGVTPFSDLTREEFRSRYHNGAAHFAAAQERARVPVNVEV
czp.1.I.2_dm28c            VFRENLFLARLHAAANPHATFGVTPFSDLTREEFRSRYHNGAAHFAAAQERARVPVNVEV
czp.1.I.10_NEL             VFRENLFLARLHAAANPHATFGVTPFSDLTREEFRSRYHNGAAHFAAAQERARVPVNVEV
czp.1.I.8_NEL              VFRENLFLARLHAAANPHATFGVTPFSDLTREEFRSRYHNGAAHFAAAQERARVPVNVEV
czp.1.I.15_NEL             VFRENLFLARLHAAANPHATFGVTPFSDLTREEFRSRYHNGAAHFAAAQERARVPVNVEV
czp.1.I.17_NEL             VFRENLFLARLHAAANPHATFGVTPFSDLTREEFRSRYHNGAAHFAAAQERARVPVNVEV
czp.1.I.7_NEL              VFRENLFLARLHAAANPHATFGVTPFSDLTREEFRSRYHNGAAHFAAAQERARVPVNVEV
czp.1.III.3_YC6            VFRENLFLARLHAAANPHATFGVTPFSDLTREEFRSRYRNGAAHFAVAQERARVPVKVEV
czp.1.III.4_YC6            VFRENLFLARLHAAANPHATFGVTPFSDLTREEFRSRYRNGAAHFAVAQERARVPVKVEV
czp.1.III.7_YC6            VFRENLFLARLHAAANPHATFGVTPFSDLTREEFRSRYRNGAAHFAVAQERARVPVKVEV
czp.1.III.2_YC6            VFRENLFLARLHAAANPHATFGVTPFSDLTREEFRSRYRNGAAHFAAAQERARVPVKVEV
czp.2.II.3_EL              VFRANLFLARLHAAANPHATFGVTPFSDLTREEFRSRYHNGAAHFAAAQERARVPVDVEV
czp.2.II.13_YC6            VFRANLFLARLHAAANPHATFGVTPFSDLTREEFRSRYHNGAAHFAAAQERARVPVDVEV
czp.2.II.10_YC6            VFRANLFLARLHAAANPHATFGVTPFSDLTREEFRSRYHNGAAHFAAAQERARVPVDVEV
czp.2.II.12_YC6            VFRANLFLARLHAAANPHATFGVTPFSDLTREEFRSRYHNGAAHFAAAQERARVPVDVEV
czp.2.II.2_dm28c           VFRANLFLARLHAAANPHATFGVTPFSDLTREEFRSRYHNGAAHFAAAQERARVPVDVEV
czp.3.II.4_EL              VFRANLFLARLHAAANPHATFGVTPFSDLTREEFRSRYHNGAAHFAAAQERARVPVDVEV
czp.3.II.14_YC6            VFRANLFLARLHAAANPHATFGVTPFSDLTREEFRSRYHNGAAHFAAAQERARVPVDVEV
czp.3.II.9_NEL             VFRANLFLARLHAAANPHATFGVTPFSDLTREEFRSRYHNGAAHFAAAQERARVPVDVEF
czp.4.II.5_NEL             VFRANLFLARLHAAANPHATFGVTPFSDLTREEFRSRYHNGAAHFAAAQERARVPVDVEF
czp.4.II.7_NEL             VFRANLFLARLHAAANPHATFGVTPFSDLTREEFRSRYHNGAAHFAAAQERARVPVDVEF
czp.4.II.3_dm28c           VFRANLFLARLHAAANPHATFGVTPFSDLTREEFRSRYHNGAAHFAAAQERARVPVDVEV
                           *** **********************************:***.***.*********.**.
```

Figure S3 - **Alignment of complete cruzipain sequences from Dm28c, CL Brener and YC6 *T. cruzi* strains**. Figure continues on the next page.

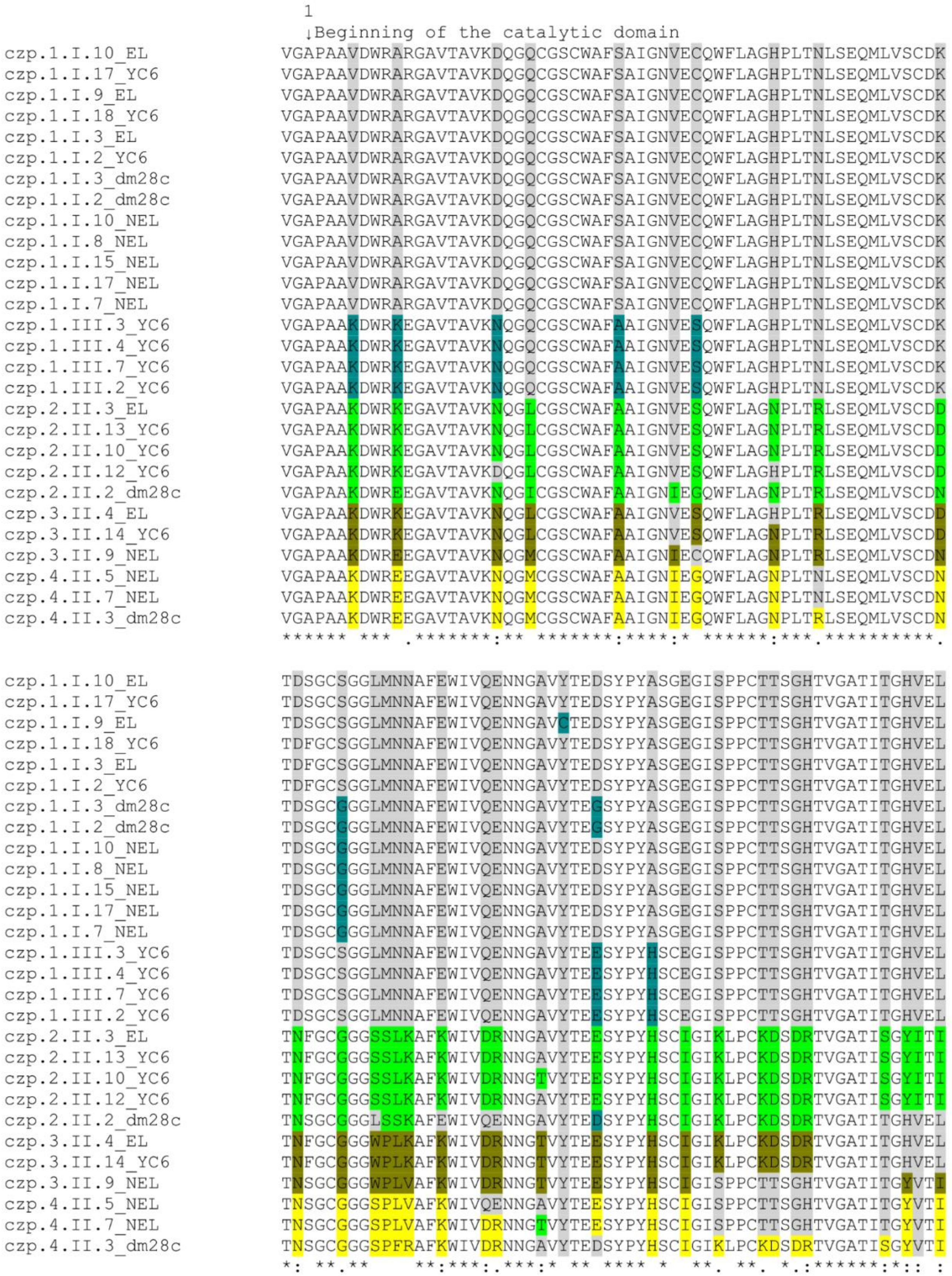

Figure S3 - **Alignment of complete cruzipain sequences from Dm28c, CL Brener and YC6 *T. cruzi* strains**. Figure continues on the next page.

```
czp.1.I.10_EL          PQDEAQIAAWLAVNGPVAVAVDASSWMTYTGGVMTSCVSEQLDHGVLLVGYNDSAAVPYW
czp.1.I.17_YC6         PQDEAQIAAWLAVNGPVAVAVDASSWMTYTGGVMTSCVSEQLDHGVLLVGYNDSAAVPYW
czp.1.I.9_EL           PQDEAQIAAWLAVNGPVAVAVDASSWMTYTGGVMTSCVSEQLDHGVLLVGYNDSAAVPYW
czp.1.I.18_YC6         PQDEAQIAAWLAVNGPVAVAVDASSWMTYTGGVMTSCVSEQLDHGVLLVGYNDSAAVPYW
czp.1.I.3_EL           PQDEAQIAAWLAVNGPVAVAVDASSWMTYTGGVMTSCVSEQLDHGVLLVGYNDSAAVPYW
czp.1.I.2_YC6          PQDEAQIAAWLAVNGPVAVAVDASSWMTYTGGVMTSCVSEQLDHGVLLVGYNDSAAVPYW
czp.1.I.3_dm28c        PQDEAQIAAWLAVNGPVAVAVDASSWMTYTGGVMTSCVSEQLDHGVLLVGYNDSAAVPYW
czp.1.I.2_dm28c        PQDEAQIAAWLAVNGPVAVAVDASSWMTYTGGVMTSCVSEQLDHGVLLVGYNDSAAVPYW
czp.1.I.10_NEL         PQDEAQIAAWLAVNGPVAVAVDASSWMTYTGGVMTSCVSEQLDHGVLLVGYNDSAAVPYW
czp.1.I.8_NEL          PQDEAQIAAWLAVNGPVAVAVDASSWMTYTGGVMTSCVSEQLDHGVLLVGYNDSAAVPYW
czp.1.I.15_NEL         PQDEAQIAAWLAVNGPVAVAVDASSWMTYTGGVMTSCVSEQLDHGVLLVGYNDSAAVPYW
czp.1.I.17_NEL         PQDEAQIAAWLAVNGPVAVAVDASSWMTYTGGVMTSCVSEQLDHGVLLVGYNDSAAVPYW
czp.1.I.7_NEL          PQDEAQIAAWLAVNGPVAVAVDASSWMTYTGGVMTSCVSEQLDHGVLLVGYNDSAAVPYW
czp.1.III.3_YC6        PQDEAQIAAWLAVNGPVAVAVDASSWMTYTGGVMTSCVSEQLDHGVLLVGYNDSAAVPYW
czp.1.III.4_YC6        PQDEAQIAAWLAVNGPVAVAVDASSWMTYTGGVMTSCVSEQLDHGVLLVGYNDSAAVPYW
czp.1.III.7_YC6        PQDEAQIAAWLAVNGPVAVAVDASSWMTYTGGVMTSCVSEQLDHGVLLVGYNDSAAVPYW
czp.1.III.2_YC6        PQDEAQIAAWLAVNGPVAVAVDASSWMTYTGGVMTSCVSEQLDHGVLLVGYNDSAAVPYW
czp.2.II.3_EL          PRDEEGIAAVLSIKGPLAVAVDATSWMSYTGGVLTSCVSKRLNHAVLLVGYNDSAAVPYW
czp.2.II.13_YC6        PRDEEGIAAVLSIKGPLAVAVDATSWMSYTGGVLTSCVSKRLNHAVLLVGYNDSAAVPYW
czp.2.II.10_YC6        PRDEEGIAAVLSIKGPLAVAVDATSWMSYTGGVLTSCVSKRLNHAVLLVGYNDSAAVPYW
czp.2.II.12_YC6        PRDEEGIAAVLSIKGPLAVAVDATSWMSYTGGVLTSCVSKRLNHAVLLVGYNDSAAVPYW
czp.2.II.2_dm28c       PQDEAQIAAWLAVKGPLSVAVDASSWFFYTGGVLTNCVSKRLSHAVLLVGYNDSAAVPYW
czp.3.II.4_EL          PQDEAQIAAWLAANGPLVATVDATSWIFYTGGVMTSCVSKTLSHAVLLVGYNDSAAVPYW
czp.3.II.14_YC6        PQDEAQIAAWLAANGPLVATVDATSWIFYTGGVMTSCVSKTLSHAVLLVGYNDSAAVPYW
czp.3.II.9_NEL         PRDENGIAAWLAVNGPVAVVVDASSWIFYTGGVMTSCVSKQLSHAVLLVGYNDSATVPHW
czp.4.II.5_NEL         PRDEKRIAAVLAVKGPLSVAVDASSWMPYTGGVLTNCVSKKLDHAVLLVGYNDSATVPYW
czp.4.II.7_NEL         PRDEKRIAAVLAVKGPLSVAVDASSWMPYTGGVLTNCVSKKLDHAVLLVGYNDSATVPYW
czp.4.II.3_dm28c       PSDEKRIAAVLAVKGPLSVAVDASSWMPYTGGVLTNCVSKKLDHAVLLVGYNDSAAVPYW
                       * **  *** *: :**: ..***:**: *****:*.***: *.*.**********:**:*

                                                                      215
                                   End of the catalytic domain↓
czp.1.I.10_EL          IIKNSWTTQWGEEGYIRIAKGSNQCLVKEEASSAVVGGPGPTPEPTTTTTTSAPGPSPSY
czp.1.I.17_YC6         IIKNSWTTQWGEEGYIRIAKGSNQCLVKEEASSAVVGGPGPTPEPTTTTTTSAPGPSPSY
czp.1.I.9_EL           IIKNSWTTQWGEEGYIRIAKGSNQCLVKEEASSAVVGGPGPTPEPTTTTTTSAPGPSPSY
czp.1.I.18_YC6         IIKNSWTTQWGEEGYIRIAKGSNQCLVKEEASSAVVGGPGPTPEPTTTTTTSAPGPSPSY
czp.1.I.3_EL           IIKNSWTTQWGEEGYIRIAKGSNQCLVKEEASSAVVGGPGPTPEPTTTTTTSAPGPSPSY
czp.1.I.2_YC6          IIKNSWTTQWGEDGYIRIAKGSNQCLVKEEASSAVVGGPGPTPEPTTTTTTSAPGPSPSY
czp.1.I.3_dm28c        VIKNSWTTQWGEDGYIRIAKGSNQCLVKEEASSAVVGGPGPTPEPTTTTTTSAPGPSPSY
czp.1.I.2_dm28c        VIKNSWTTQWGEDGYIRIAKGSNQCLVKEEASSAVVGGPGPTPEPTTTTTTSAPGPSPSY
czp.1.I.10_NEL         IIKNSWTAQWGEDGYIRIAKGSNQCLVKEEASSAVVGGPGPTPEPTTTTTTSAPGPSPSY
czp.1.I.8_NEL          IIKNSWTAQWGEDGYIRIAKGSNQCLVKEEASSAVVGGPGPTPEPTTTTTTSAPGPSPSY
czp.1.I.15_NEL         IIKNSWTAQWGEDGYIRIAKGSNQCLVKEEASSAVVGGPGPTPEPTTTTTTSAPGPSPSY
czp.1.I.17_NEL         IIKNSWTAQWGEDGYIRIAKGSNQCLVKEEASSAVVGGPGPTPEPTTTTTTSAPGPSPSY
czp.1.I.7_NEL          IIKNSWTAQWGEDGYIRIAKGSNQCLVKEEASSAVVGGPGPTPEPTTTTTTSAPGPSPSY
czp.1.III.3_YC6        IIKNSWTTQWGEEGYIRIAKGSNQCLVKEEASSAVVGGPGPTPEPTTTTTTSAPGPSPSY
czp.1.III.4_YC6        IIKNSWTTQWDEEGYIRIAKGSNQCLVKEEASSAVVGGPGPTPEPTTTTTTSAPGPSPSY
czp.1.III.7_YC6        IIKNSWTTQWGEEGYIRIAKGSNQCLVKEEASSAVVLRSRPTPEPTTTTTTSAPGPSPSY
czp.1.III.2_YC6        IIKNSWTTQWGEEGYIRIAKGSNQCLVKEEASSAVVGGPGPTPEPTTTTTTSAPGPSPSY
czp.2.II.3_EL          IIKNSWTTHWGEEGYIRIAKGSNQCLVKEEASSAVVLRSRPTPEPTTTTTTSAPGPSPSY
czp.2.II.13_YC6        IIKNSWTTHWGEEGYIRIAKGSNQCLVKEEASSAVVLRSRPTPEPTTTTTTSAPGPSPSY
czp.2.II.10_YC6        IIKNSWTTHWGEEGYIRIAKGSNQCLVKEEASSAVVLRSRPTPEPTTTTTTSAPGPSPSY
czp.2.II.12_YC6        IIKNSWTTHWGEEGYIRIAKGSNQCLVKEEASSAVVLRSRPTPEPTTTTTTSAPGPSPSY
czp.2.II.2_dm28c       IIKNSWTTHWGEGGYIRIAKGSNQCLVKEEVSSAVVGGPGTTPEPTTTTTTSAPGPSPSY
czp.3.II.4_EL          IIKNSWTTHWGEEGYIRIAKGSNQCLVKEEASSAVVLRSRPTPEPTTTTTTSAPGPSPSY
czp.3.II.14_YC6        IIKNSWTTHWGEEGYIRIAKGSNQCLVKEEASSAVVLRSRPTPEPTTTTTTSAPGPSPSY
czp.3.II.9_NEL         IIKNSWTTYWGEDGYIRIAKGSNQCLVKEEASSAVVGGPGATPEPTTTTTTSAPGPSPSY
czp.4.II.5_NEL         IIKNSWTTQWGEDGYIRIAKGSNQCLVKEGVSSAVVGGPGATPEPTTTTTTSAPGPSPSY
czp.4.II.7_NEL         IIKNSWTTHWGEDGYIRIAKGSNQCLVKEGVSSAVVGGPGATPEPTTTTTTSAPGPSPSY
czp.4.II.3_dm28c       IIKNSWTTHWGEGGYIRIAKGSNQCLVKEGVSSAVVGGPGTTPETTTTTTTSAPGPSPSY
                       :******: *.* **************** .*****  . .***.***************
```

Figure S3 - **Alignment of complete cruzipain sequences from Dm28c, CL Brener and YC6 *T. cruzi* strains**. Figure continues on the next page.

```
czp.1.I.10_EL         FVQMSCTDAACIVGCENVTLPTGQCLLTTSGVSAIVTCGAETLTEEVFLTSTHCSGPSVR
czp.1.I.17_YC6        FVQMSCTDAACIVGCENVTLPTGQCLLTTSGVSAIVTCGAETLTEEVFLTSTHCSGPSVR
czp.1.I.9_EL          FVQMSCTDAACIVGCENVTLPTGQCLLTTSGVSAIVTCGAETLTEEVFLTSTHCSGPSVR
czp.1.I.18_YC6        FVQMSCTDAACIVGCENVTLPTGQCLLTTSGVSAIVTCGAETLTEEVFLTSTHCSGPSVR
czp.1.I.3_EL          FVQMSCTDAACIVGCENVTLPTGQCLLTTSGVSAIVTCGAETLTEEVFLTSTHCSGPSVR
czp.1.I.2_YC6         FVQMSCTDAACIVGCENVTLPTGQCLLTTSGVSAIVTCGAETLTEEVFLTSTHCSGPSVR
czp.1.I.3_dm28c       FVQMSCTDAACIVGCENVTLPTGQCLLTTSGVSAIVTCGAETLTEEVFLTSTHCSGPSVR
czp.1.I.2_dm28c       FVQMSCTDAACIVGCENVTLPTGQCLLTTSGVSAIVTCGAETLTEEVFLTSTHCSGPSVR
czp.1.I.10_NEL        FVQMSCTDAACIVGCENVTLPTGQCLLTTSGVSAIVTCGAETLTEEVFFTSTHCSGPSVR
czp.1.I.8_NEL         FVQMSCTDAACIVGCENVTLPTGQCLLTTSGVSAIVTCGAETLTEEVFFTSTHCSGPSVR
czp.1.I.15_NEL        FVQMSCTDAACIVGCENVTLPTGQCLLTTSGVSAIVTCGAETLTEEVFFASTHCSGPSVR
czp.1.I.17_NEL        FVQMSCTEAACIVGCENVTLPTGQCLLTTSGVSAIVTCGAETLTEEVFFTSTHCSGPSVR
czp.1.I.7_NEL         FVQMSCTDAACIVGCENVTLPTGQCLLTTSGVSAIVTCGAETLTEEVFFTSTHCSGPSVR
czp.1.III.3_YC6       FVQMSCTDAACIVGCENVTLPTGQCLLTTSGVSAIVTCGAETLTEEVFLTSTHFSGPSVR
czp.1.III.4_YC6       FVQMSCTDAACIVGCENVTLPTGQCLLTTSGVSAIVTCGAETLTEEVFLTSTHFSGPSVR
czp.1.III.7_YC6       FVQMSCTDAACIVGCENVTLPTGQCLLTTSGVSAIVTCGAETLTEEVFLTSTHFSGPSVR
czp.1.III.2_YC6       FVQMSCTDAACIVGCENVTLPTGQCLLTTSGVSAIVTCGAETLTEEVFLTSTHCSGPSVR
czp.2.II.3_EL         FVQMSCTDAACIVGCENVTLPTGQCLLTTSGVSAIVTCGAETLTEEVFLTSTHCSGPSVR
czp.2.II.13_YC6       FVQMSCTDAACIVGCENVTLPTGQCLLTTSGVSAIVTCGAETLTKEVFLTSTHCSGPSVR
czp.2.II.10_YC6       FVQMSCTDAACIVGCENVTLPTGQCLLTTSGVSAIVTCGAETLTEEVFLTSTHCSGPSVR
czp.2.II.12_YC6       FVQMSCTDAACIVGCENVTLPTGQCLLTTSGVSAIVTCGAETLTEEVFLTSTHCSGPSVR
czp.2.II.2_dm28c      FVQMSCTDAACSVGCENVTLPTGQCLLTTSGVSAIVTCGAETLTEEVFLTSTHCSGPSVR
czp.3.II.4_EL         FVQMSCTDAACIVGCENVTLPTGQCLLTTSGVSAIVTCGAETLTEEVFLTSTHCSGPSVR
czp.3.II.14_YC6       FVQMSCTDAACIVGCENVTLPTGQCLLTTSGVSAIVTCGAETLTEEVFLTSTHCSGPSVR
czp.3.II.9_NEL        FVQMSCTDAACIVGCENVTLPTGQCLLTTSGASAIVTCGAETLTEEVFLTSTHCSGPSVR
czp.4.II.5_NEL        FVQMSCTDVACIVGCENVTLPTGQCLLTTSGASAIVTCGAETLTEEVFLTSTHCSGPSVR
czp.4.II.7_NEL        FVQMSCTDAACIVGCENVTLPTGQCLLTTSGASAIVTCGAETLTEEVFLTSTHCSGPSVR
czp.4.II.3_dm28c      FVQMSCTDAACSVGCENVTLPTGQCLLTTSGVSAIVTCGAETLTEEVFLTSTHCSGPSVR
                      *******:.** *****************.************:***::*** ******
```

```
                                                                  467
                                                                  ↓
czp.1.I.10_EL         SSVPLNKCNRLLRGSVEFFCGSSSSGRLADVDRQRRHQPYHSRHRRL-
czp.1.I.17_YC6        SSVPLNKCNRLLRGSVEFFCGSSSSGRLADVDRQRRHQPYHSRHRRL-
czp.1.I.9_EL          SSVPLNKCNRLLRGSVEFFCGSSSSGRLADVDRQRRHQPYHSRHRRL-
czp.1.I.18_YC6        SSVPLNKCNRLLRGSVEFFCGSSSSGRLADVDRQRRHQPYHSRHRRL-
czp.1.I.3_EL          SSVPLNKCNRLLRGSVEFFCGSSSSGRLADVDRQRRHQPYHSRHRRL-
czp.1.I.2_YC6         SSVPLNKCNRLLRGSVEFFCGSSSSGRLADVDRQRRHQPYHSRHRRL-
czp.1.I.3_dm28c       SSVPLNKCNRLLRGSVEFFCGSSSSGRLADVDRQRRYQPYHSRHRRL-
czp.1.I.2_dm28c       SSVPLNKCNRLLRGSVEFFCGSSSSGRLADVDRQRRYQPYHSRHRRL-
czp.1.I.10_NEL        SSVPLNQCNWLLRGSVEFFCGSSSSGRLADVDRQRRYQPYQSRHRRL-
czp.1.I.8_NEL         SSVPLNQCNWLLRGSVEFFCGSSSSGRLADVDRQRRYQPYQSRHRRL-
czp.1.I.15_NEL        SSVPLNQCNWLLRGSVEFFCGSSSSGRLADVDRQRRYQPYHSRHRRL-
czp.1.I.17_NEL        SSVPLNQCNWLLRGSVEFFCGSSSSGRLADVDRQRRYQPYHSRHRRL-
czp.1.I.7_NEL         SSVPLNQCNRLLRGSVEFFCGSSSSGRLADVDRQRRYQPYQSRHRRL-
czp.1.III.3_YC6       SSVPLNKCNRLLRGSVEFFCGSSSSGRLADVDRQRRHQPYHSRHRRL-
czp.1.III.4_YC6       SSVPLNKCNRLLRGSVEFFCGSSSSGRLADVDRQRRHQPYHSRHRRL-
czp.1.III.7_YC6       SSVPLNKCNRLLRGSVEFFCGSSSSGRLADVDRQRRHQPYHSRHRRL-
czp.1.III.2_YC6       SSVPLNKCNRLLRGSVEFFCGSSSSGRLADVDRQRRHQPYHSRHRRL-
czp.2.II.3_EL         SSVPLNKCNRLLRGSVEFFCGSSSSGRLADVDRQRRYQPYHSRHRRL-
czp.2.II.13_YC6       SSVPLNKCNRLLRGSVEFFCGSSSSGRLADVDRQRRYQPYHSRHRRL-
czp.2.II.10_YC6       SSVPLNKCNRLLRGSVEFFCGSSSSGRLADVDRQRRYQPYHSRHRRL-
czp.2.II.12_YC6       SSVPLNKCNRLLRGSVEFFCGSSSSGRLADVDRQRRYQPYHSRHRRL-
czp.2.II.2_dm28c      SSVPLNKCNRLIRGSVEFFRSSNSSGRLADVDRQRRYQPYHSRHRRL-
czp.3.II.4_EL         SSVPLNKCNRLLRGSVEFFCGSSSSGRLADVDRQRRYQPYHSRHRRL-
czp.3.II.14_YC6       SSVPLNKCNRLLRGSVEFFCGSSSSGRLADVDRQRRYQPYHSRHRRL-
czp.3.II.9_NEL        SSVPLNKCNRLLRGSVEFFRGSSSSGRLADVDRQRRYQPYHSRHRRL-
czp.4.II.5_NEL        SSVPLNKCNRLLRGSVEFFRGSSSSGRLADVDRQRRYQPYHSRHRRL-
czp.4.II.7_NEL        SSVPLNKCNRLLRGSVEFFRGSSSSGRLADVDRQRRYQPYHSRHRRL-
czp.4.II.3_dm28c      SSVPLNKCNRLIRGSVEFFRSSNSSGRLADVDRQRRYQPYHSRHRRL-
                      ******:** *:******* .*.*************:***:******
```

Figure S3. **Alignment of complete cruzipain sequences from Dm28c, CL Brener and YC6 *T. cruzi* strains**. Alignment performed with Clustal O (1.2.4). All cruzipains sequences were aligned and the duplicates removed to generate the figure. Sites with varying residues are colored according to the following scheme: in gray when they are equal to one present in the first sequence; otherwise, they are colored dark green if they belong to a czp1 sequence, light green for czp2, gold for czp3, and yellow for czp4.

A

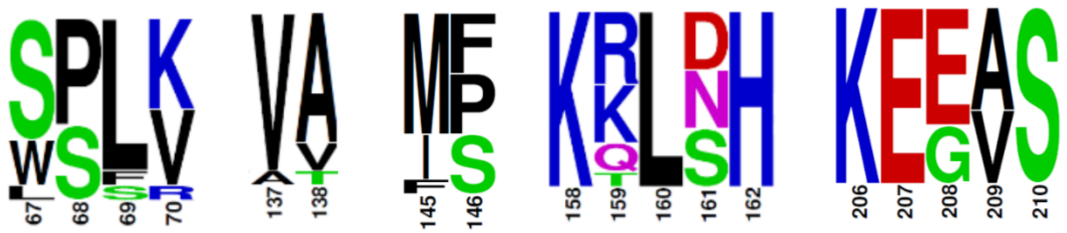

B

Cruzipain 2

CL Brener	…**SS**LK…	…VA…	…MS…	…KRLNH…	…KEEAS…
Dm28c	…**SS**LK…	…VA…	…FF…	…KRLSH…	…KEEVS…
YC6	…**SS**LK…	…VA…	…MS…	…KRLNH…	…KEEAS…

Cruzipain 3

CL Brener	…**WP**L[K/V]…	…[A/V][T/V]…	…IF…	…K[T/Q]LSH…	…KEEAS…
YC6	…**WP**LK…	…AT…	…IF…	…KTLSH…	…KEEAS…
TCC	…**WP**LV…	…VA…	…MT…	…KQLSH…	…KEEAS…

Cruzipain 4

CL Brener	…**SP**LV…	…VA…	…MP…	…KKLDH…	…KEGVS…
Dm28c	…**SP**FR…	…VA…	…MP…	…KKLDH…	…KEGVS…
TCC	…[**S/W**]PLV…	…VA…	…LT…	…KKLDH…	…KE[G/A]VS…

Figure S4**. Alignment of the regions selected for the definition of cruzipains sub-types.** A) Sequence logo generated with WebLogo using the multiple alignment of cruzipains sequences of cluster II. We aligned 12 cruzipain sequences from CL Brener, Dm28c, YC6 and TCC strains. Numbers indicate residue number (considering as residue 1 the first residue of the catalytic domain), and the letters, amino acids residues. The height of the letters indicates the relative frequency of each amino acid in that position. B) Alignment of each region used to define the sub-types showing for each strain we used in our studies the observed amino acids.

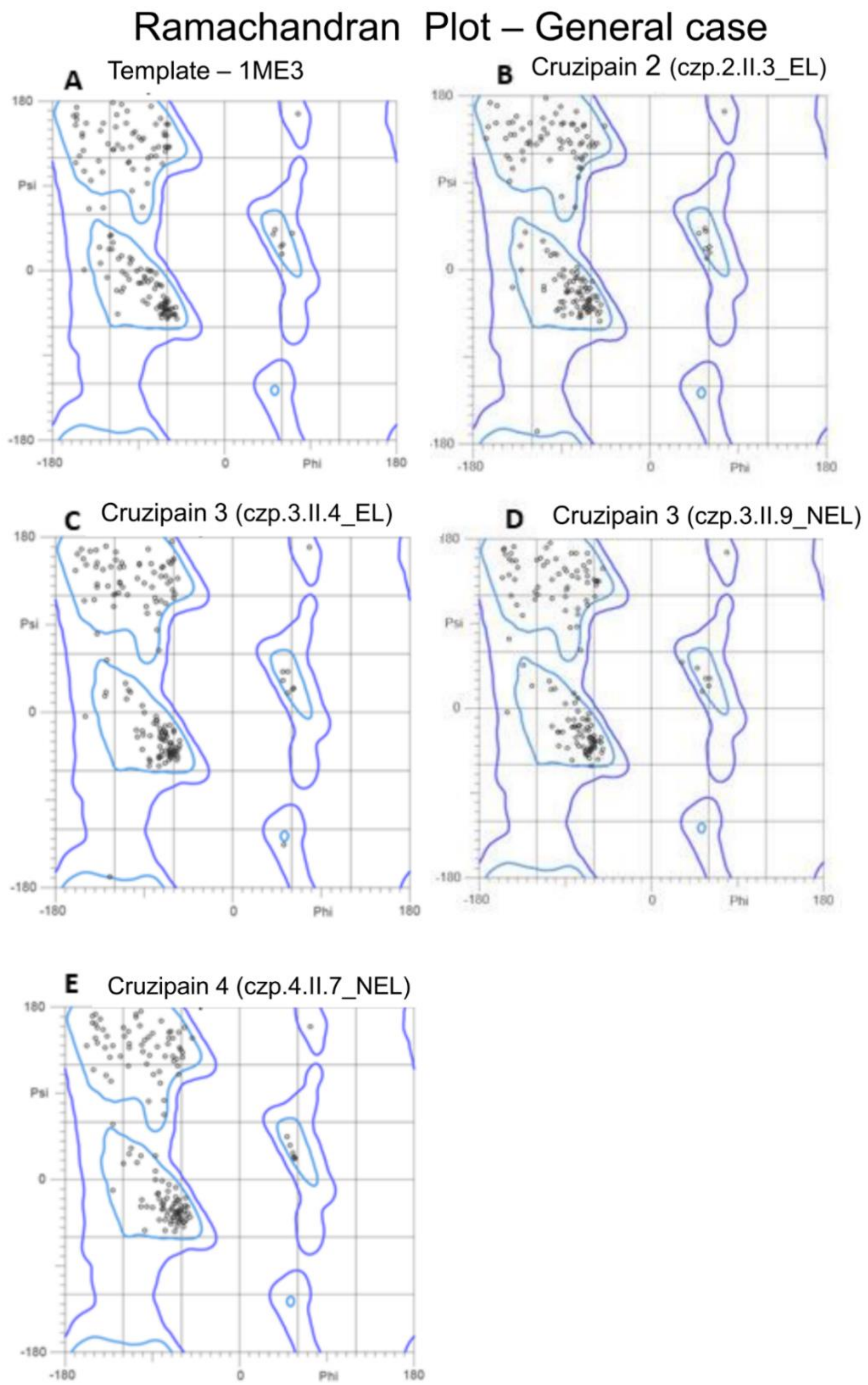

Figure S5. **Ramachandran plot revealed all amino acids residues in allowed regions for cruzipain models.** A- Crystal template of cruzain (from cruzipain 1 sub-type, PDB ID 1ME3). B – E Theoretical models for cruzipains from sub-type 2 (czp.2.II.3_EL), 3 (czp.3.II.4_EL and czp.3.II.9_NEL), and 4 (czp.4.II.7_NEL). The x-axis shows phi angles and y-axis, psi angles. Light blue lines delimit favored angle regions and dark blue lines, allowed regions. Graphs were obtained in the Molprobity server.

Table S6. **Summary of results obtained for validation of cruzipain models with Pymol, Molprobity, ERRAT and Swiss Model.**

Server or Software	Evaluation	Model Assessed				
		**Template PDB 1ME3**	**czp.2.II.3_ EL**	**czp.3.II.4_ EL**	**czp.3.II.9_ NEL**	**czp.4.II.7_ NEL**
Pymol	RMS C-alpha[a] to template (Å)	0	0.161	0.152	0.147	0.157
	Gln19 rotamer compatible with oxyanion hole?	Yes	Yes	Yes	Yes	Yes
	Cys25 and His162 in catalytic configuration?	Yes	Yes	Yes	Yes	Yes
Molprobity	Ramachandran Favored[b] (%)	97.18	96.73	95.79	94.84	96.71
	Ramachandran Outliers[c] (%)	0	0	0	0	0
	Rotamer outliers[d] (%)	1.14	4.68	6.43	5.85	4.09
	C-beta deviation[e] (%)	0	0.52	1.04	1.04	1.04
	Bad Angles[f] (%)	0.84	0.80	0.67	0.75	0.62
	Bad Bonds[g] (%)	0	0	0	0	0
ERRAT	Quality Factor ERRAT[h]	91.13	84.62	87.50	94.20	81.07
Q-MEAN	Q-MEAN Score[i]	0.45	-0.67	-0.59	-0.55	-0.01

[a] RMS C-alpha values close to zero indicate high structural similarity to the template structure. Molprobity cutoffs (%): [b] Good: Favored > 95, Caution: 98 > Favored ≥ 95, Warning: Favored < 95 [c] Good: Outlier < 0.05, Caution: 0.05 < Outlier ≤ 0.05, Warning: Outlier >0.05. [d] Good: Outlier ≤ 0.3, Caution: 0.3 < Outliers ≤ 1.5, Warning: Outliers > 1.5 . [e] Good: Outlier = 0; Caution: 0 < Outliers < 5; Warning: Outliers ≥ 5. [f] Good: Outlier < 0.1%; Caution: 0.1% ≤ Outlier angles < 0.5%; Warning: Outlier angles ≥ 0.5%; [g] Good: Outlier bonds < 0.01%; Caution: 0.01% ≤ Outlier bonds < 0.2%; Warning: Outlier bonds ≥ 0.2%; [h] Values range from 0 to 100. Higher values indicate a lower chance of errors in the structure. [i] The maximum value is 1, and values closer to 1 indicate more reliable structures.

Table S7. **Intermolecular interactions among cruzain and inhibitors from crystallography complexes.**

**Residue**	**Type of interaction**[a]	**Interaction frequency**[b] **(%)**
Gln19	Hydrogen bond	20.8
Cys25	Hydrophobic	70.8
Trp26	*Total*	*12.5*
	Hydrophobic	8.3
	Aromatic stacking	4.2
Ser29	Hydrogen bond	4.2
Ser64	Hydrogen bond (water)	4.2
Gly66	Hydrogen bond (N)	58.3
	Hydrogen bond (O)	50.0
Leu67	Hydrophobic	79.2
Met68	Hydrophobic	70.8
Ala138	Hydrophobic	83.3
Ala141	Hydrophobic	25.0
Met145	Hydrophobic	29.2
Gln159	Hydrophobic	4.2
Leu160	*Total*	*70.8*
	Hydrophobic	58.3
	Hydrogen bond (water)	12.5
	Hydrogen bond	4.2
Asp161	*Total*	*50.0*
	Hydrogen bond	37.5
	Hydrophobic	12.5
	Repulsive	4.2
	Hydrogen bond (water)	4.2
	Attractive	4.2
His162	*Total*	*50.0*
	Hydrophobic	33.3
	Aromatic stacking	20.8
	Attractive	8.3
	Repulsive	4.2
	Hydrogen bond	4.2
Trp184	*Total*	*50.0*
	Hydrophobic	33.3
	Hydrogen bond	33.3
	Aromatic stacking	29.2
	Hydrogen bond (water)	8.3
Glu208	*Total*	*41.7*
	Hydrophobic	29.2
	Attractive	12.5
	Hydrogen bond	4.2

[a] For residues that perform multiple types of interactions with ligands, we report the frequency of each type of interaction and the overall interaction frequency considering all types of interactions (total) [b] frequency determined based on 24 cruzain complexes available in the Protein Data Bank, as identified by the software nAPOLI (http://bioinfo.dcc.ufmg.br/napoli/).
